# Climate, ecological dynamics, and the seasonal distribution of birds in mountains

**DOI:** 10.1126/sciadv.adz5547

**Published:** 2026-02-06

**Authors:** Marius Somveille, Benjamin G. Freeman, Frank A. La Sorte, Mao-Ning Tuanmu

**Affiliations:** ^1^ School of Environmental Sciences, University of East Anglia, Norwich, UK.; ^2^ Centre for Biodiversity and Environment Research, Department of Genetics, Evolution and Environment, University College London, London, UK.; ^3^ School of Biological Sciences, Georgia Institute of Technology, Atlanta, GA, USA.; ^4^ Department of Ecology and Evolutionary Biology, Yale University, New Haven, CT, USA.; ^5^ Center for Biodiversity and Global Change, Yale University, New Haven, CT, USA.; ^6^ Biodiversity Research Center, Academia Sinica, Taipei, Taiwan.

## Abstract

Biodiversity is unevenly distributed along elevational gradients. The predominant hypothesis is that macroevolutionary dynamics and climatic niche conservatism explain today’s elevational patterns of biodiversity, but the alternative energy efficiency hypothesis emphasizes modern ecological interactions related to energy budgets. We test these competing hypotheses by examining seasonal elevational ranges for 10,998 bird populations in 34 mountain regions. Multiple lines of evidence support the energy efficiency hypothesis, including that many mountain birds do not seasonally track their thermal niche with high fidelity while simulation models based on optimal energy balancing under current environmental conditions yield predictions that tightly match empirical data. Our results reveal that altitudinal migration, which is widespread yet considerably understudied, is a behavioral mechanism fulfilling the same ecological function as long-distance latitudinal migration. Overall, this work provides a better understanding and predictive capacity for mountain birds under global change.

## Teaser

Ecological dynamics related to energy use and competition drives the seasonal distribution of birds in mountains across the world.

## INTRODUCTION

Elevational gradients across mountainous regions vary in climate and productivity and thus provide excellent natural replicates for investigating the processes driving biodiversity patterns. Most taxa exhibit one of three patterns: a decrease in species richness with increasing elevation, a high-diversity plateau at low elevation followed by a monotonic decrease in diversity with increasing elevation, or a mid-elevation peak in diversity (*1*–*5*). The predominant explanation for these patterns focuses on macroevolutionary dynamics and climatic history—hereafter the “macroevolutionary niche conservatism” hypothesis. This hypothesis posits that climatic zones along an elevational gradient accumulate species largely independently and at different rates (*5*–*9*). Climate niche conservatism, i.e., descendant species having similar climate niche to ancestor species, and increasing adaptation to climatic zones over time together lead to relatively narrow fundamental niches and species being largely confined to their climate zone because they are poorly adapted to alternative climate (*7*, *8*).

An alternative explanation is that species diversity patterns along mountain slopes are primarily driven by the availability of energy at present (*3*). According to the maximum power principle (*10*), natural selection should favor organisms that have the most advantageous balance between the energy expenditure of their lifestyle and the benefits in terms of access to available energy to fuel metabolism, as they can invest more energy into reproduction and growth (*11*). Applying this principle to a mountain slope, which acts as an ecological arena where species compete for resources, the “energy efficiency” hypothesis predicts that species should distribute in a way that minimizes energetic costs while maximizing energy acquisition by targeting areas and elevations with better access to energy supply, as a function of what competitors are doing (*12*). This hypothesis posits that species have relatively broad fundamental niches, which could particularly apply to endotherms as they can survive temperatures far from optimal by paying an energetic cost for thermoregulation (*13*), and it emphasizes the dynamic response to current biotic and abiotic conditions in shaping species’ elevational distributions.

Here, we test predictions of the macroevolutionary niche conservatism and energy efficiency hypotheses by examining global patterns of avian distribution along elevational gradients. We follow a tradition of using different mountain regions as replicates for investigating the processes driving biodiversity patterns (*14*), and we use seasonality as a natural experiment, reasoning that either explanation for the distribution of diversity along elevational gradients must be able to explain how these patterns change with the seasons. A potentially large number of mobile species modify their elevational range throughout the year via altitudinal migration, which typically involves annual, return movements between breeding and nonbreeding grounds that are at different elevations (*15*). Hence, these movements are analogous to long-distance seasonal migrations, albeit at the smaller scale of elevational gradients in mountainous areas. Here, we focus on birds, which are perhaps the most mobile and well-studied group of animals. While roughly 10% of avian species worldwide are suggested to be altitudinal migrants (*16*), available data on bird altitudinal migration are sparse and geographically biased (*15*, *16*).

Much like long-distance seasonal migration across latitudes, altitudinal migration has been proposed to be an adaptation that birds use to cope with seasonality (*15*, *17*). Thus, during periods of global cooling and increasing seasonality, in particular during the Miocene (*18*), birds might have evolved altitudinal migration to persist in seasonal mountain areas by escaping the harsh conditions at high elevation during the nonbreeding season, in a similar way to how they might have evolved long-distance migration (*17*–*19*). In this scenario, seasonal climate tracking along elevational gradients, achieved through altitudinal migration, is a mechanism that allows species to stay within the climatic niche to which they are adapted. Thus, according to the macroevolutionary niche conservatism hypothesis, altitudinal migration is caused by seasonal climate tracking. In contrast, the energy efficiency hypothesis predicts that species optimize the way they distribute seasonally along elevational gradients based on current environmental conditions and ecological interactions, with altitudinal migration being a behavioral strategy for species to further optimize energy balancing. According to this hypothesis, the variation in seasonal climate tracking across altitudinal migrant species is largely a geographical consequence rather than a direct driver of species’ elevational distributions.

**Fig. 1.**
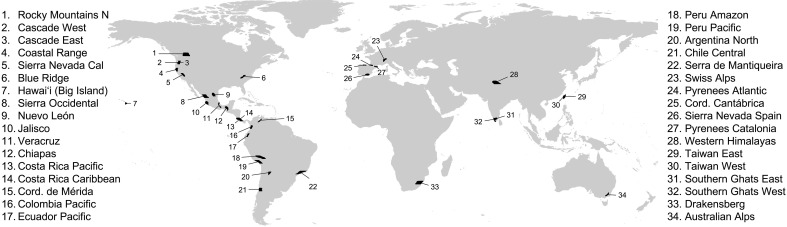
The global distribution of mountain slopes used in this study. Black polygons on the map indicate the location of the 34 mountain slopes used in the analysis.

We tested these different hypotheses by using participatory science data to characterize seasonal elevational ranges for 10,998 populations, belonging to 2684 different species, across 34 mountain slopes distributed worldwide (Fig. 1; see Materials and Methods). To test the prediction from the macroevolutionary niche conservatism hypothesis that altitudinal migration is driven by seasonal climate tracking, we quantified the degree of seasonal thermal tracking for the species in our dataset and how it relates to species’ migratory movements. We expect that altitudinal migration should be largely absent from regions with little-to-no temperature seasonality and that altitudinal migrants should seasonally track thermal conditions better than if they did not move. To test predictions from the energy efficiency hypothesis, we simulated patterns of seasonal avian diversity along elevational gradients using a modified version of the Seasonally Explicit Distributions Simulator [SEDS; (*12*)], a model originally developed to explain the global seasonal distribution of birds across latitudes based on the energy efficiency hypothesis, and we then contrasted the model predictions with empirical patterns. The SEDS model is a mechanistic model of the geographical distribution of bird species throughout the year, built from first ecological and energetics principles (see details of the model in Materials and Methods). It is a cost-benefit model that relates the energy supply available in the environment with the energetic requirements of bird populations, with the latter including mainly the costs for thermoregulation and reproduction. We assumed that the energetic costs for altitudinal movement were negligible in populations’ annual energy budgets given the high mobility of birds and the short distances involved.

## RESULTS AND DISCUSSION

**Fig. 2.**
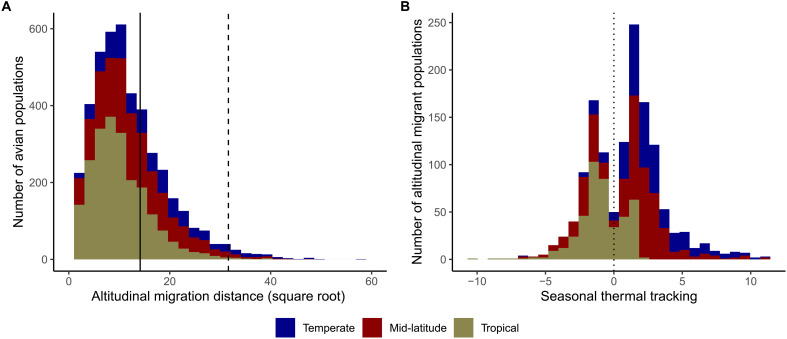
Altitudinal migration and the seasonal tracking of thermal conditions. (**A**) Distribution of altitudinal migration distances across all avian populations living year-round on the mountain slopes in our dataset. The vertical solid line indicates 200 m, i.e., the threshold above which we considered populations to be altitudinal migrants, and the vertical dashed line indicates 1000 m. (**B**) Distribution of seasonal thermal tracking across all altitudinal migrant populations on the mountain slopes in our dataset. Seasonal thermal tracking was calculated for each population as the mean seasonal thermal difference of sedentary populations in the mountain slope minus the seasonal thermal difference of the altitudinal migrant population; a negative value indicates that the population is tracking temperature worse than the local sedentary populations. The three colors indicate how the number of populations is decomposed into those occurring in tropical (between 15°S and 15°N), mid-latitude (between 15°S and 35°S or between 15°N and 35°N), and temperate (>35°S or >35°N) regions.

Overall, we found that altitudinal migration is common, providing the raw material to test our hypotheses: 31.1% of avian populations living year-round on the mountain slopes in our dataset are altitudinal migrants, which is defined as having mean seasonal altitudes separated by more than 200 m. This result confirms that avian altitudinal migration is a notable phenomenon globally. Among altitudinal migrants, few populations (96) radically shift their distribution along the elevational gradient (i.e., more than 1000 m on average; Fig. 2A), mirroring the pattern observed for latitudinal migration where few avian species migrate very long distances [e.g., trans-equatorial migrants; (*20*)]. We also found that the proportion of altitudinal migrants in a mountain region increases with latitude (Pearson’s correlation, *r* = 0.44, *P* = 0.009), supporting the hypothesis that altitudinal migration is an adaptation to seasonality.

Our results were largely inconsistent with the seasonal climate tracking prediction derived from the macroevolutionary niche conservatism hypothesis. We found altitudinal migration to be widespread within the equatorial tropics despite minimal temperature seasonality in these regions: 339 of 1852 populations (18.3%) located between 10°S and 10°N are altitudinal migrants. More generally, 36.5% of altitudinal migrant populations in our global dataset are not tracking temperature seasonally as much as if they had simply stayed sedentary (Fig. 2B; similarly high proportions were obtained with more stringent thresholds for determining altitudinal migrants, as shown in table S1; for context, fig. S1 shows how temperature seasonality varies with latitude and elevation in our dataset). For instance, more than one in four altitudinal migrant populations (27.4%) experiencing marked temperature seasonality at mid-latitudes (i.e., defined here as mountain slopes located between 15°S and 35°S or 15°N and 35°N) actively expands its thermal niche across seasons (i.e., would have tracked thermal conditions better if sedentary). This is mainly due to these populations engaging in upslope migration during the colder season, which our analysis reveals as being a relatively widespread phenomenon despite previously receiving very little attention [but see (*21*)]. These results suggest that seasonal thermal niche tracking per se is not the major cause of altitudinal migration in birds.

**Fig. 3.**
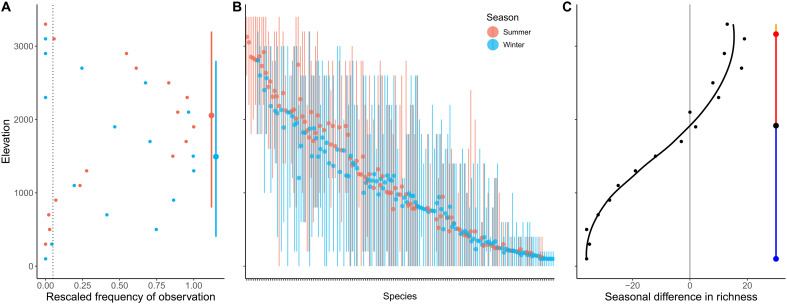
Method for estimating the seasonal difference in richness along elevational gradients. (**A**) Rescaled frequency of observation for the Taiwan Yuhina, *Yuhina brunneiceps*, at each season for each 200-m elevational bin across the Taiwan East mountain slope. The dotted vertical line indicates the threshold of 0.05 (other thresholds were also explored in a sensitivity analysis), which was used to define the lower and upper boundaries of the species’ seasonal elevational ranges (as the lowest and highest elevational bins in which the species observation frequency was above the threshold). The red and blue vertical segments indicate the resulting seasonal elevational ranges, with the points in the segments indicating the weighted mean of the species distribution across the elevational gradient for each season. (**B**) Seasonal elevational ranges of all avian species in Taiwan East, with each vertical line indicating the elevational range of a given species, during the boreal summer (in red) or boreal winter (in blue), and with points indicating the seasonal mean elevations. (**C**) Pattern of seasonal difference in richness for Taiwan East, calculated as avian richness during the boreal summer minus avian richness during the boreal winter for each 200-m elevational bin. The black line indicates the smooth spline fitted to the data points along the elevational gradient. Blue point, boreal winter peak in richness; red point, boreal summer peak in richness; black point, transition between elevations that are net receivers of migratory species during the boreal winter (blue line) and net receivers of migratory species during the boreal summer (red line).

**Fig. 4.**
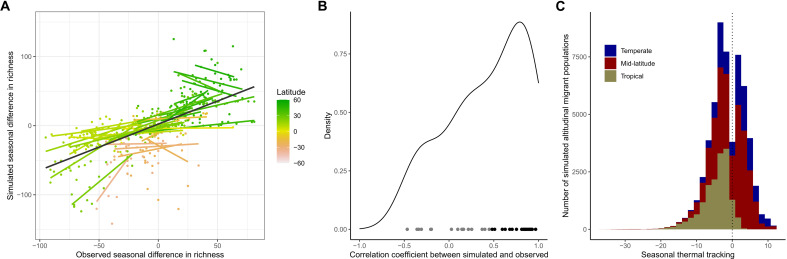
Empirical patterns associated with avian altitudinal migration are predicted by the energy efficiency hypothesis. (**A**) Relationship between the seasonal difference in species richness simulated by the model testing the energy efficiency hypothesis and estimated empirically. Each slope corresponds to a line of best fit for a mountain slope, and each point corresponds to a 200-m elevational bin, both color-coded by latitude. The black line indicates the line of best fit for the whole dataset. (**B**) Density plot of the Pearson’s correlation coefficients between model predictions and observation for the seasonal difference in species richness, for all the mountain slopes in our dataset. Gray and black points indicate correlation coefficients that are nonsignificant and significant, respectively. (**C**) Distribution of seasonal thermal tracking of simulated altitudinal migrant populations across all mountain slopes. Seasonal thermal tracking was calculated for each simulated population as the mean seasonal thermal difference of simulated sedentary populations in the mountain slope minus the seasonal thermal difference of the simulated altitudinal migrant population; a negative value indicates that the simulated population is tracking temperature worse than the local simulated sedentary populations. Colors on the histogram indicate how the number of populations is decomposed into populations occurring in tropical (between 15°S and 15°N), mid-latitude (between 15°S and 35°S or between 15°N and 35°N), and temperate (>35°S or >35°N) regions.

**Fig. 5.**
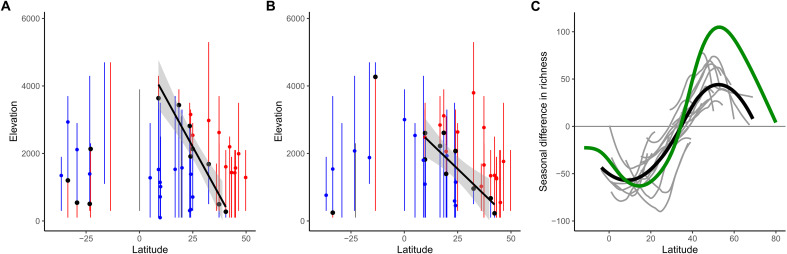
The seasonal distribution of birds in mountains is a compressed version of their global latitudinal seasonal distribution. The first two show the features of the patterns of seasonal difference in species richness for all the mountain slopes in our dataset and across latitude, derived from (**A**) empirical data and (**B**) model simulation. Blue points, boreal winter peaks in richness; red points, boreal summer peaks in richness; black points, transitions between elevations that have a net surplus of species during the boreal winter (blue line) and a net surplus of species during the boreal summer (red line). See Fig. 3 for details of how this was estimated. The black lines indicate the lines of best fit between latitude and the elevation of the transition points in the Northern Hemisphere. We focused here on the Northern Hemisphere because only two mountain slopes in the Southern Hemisphere exhibited a simulated transition point. (**C**) Projected patterns of seasonal difference in species richness along the latitudinal gradient for the mountain slopes of the Northern Hemisphere (gray lines; minus Hawaiʻi as it shows very little seasonal variation in richness), which was projected using the following formula derived from the black line in (A): mean latitude of the mountain slope + (elevation / 115) − adj, with adj = 9.6 used to align the transition points of the mean pattern across the mountain slopes (black line) and the global latitudinal pattern estimated in (*34*) (green line).

**Fig. 6.**
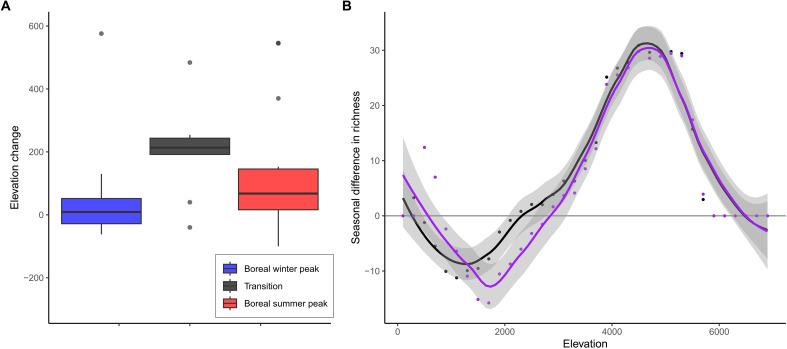
Predicted effect of climate warming on the seasonal distribution of birds in mountains. (**A**) Change in the elevation of boreal winter peaks (in blue), boreal summer peaks (in red), and transition points between elevations that have a net surplus of species during the boreal winter and a net surplus of species during the boreal summer (in black), between model predictions under current and future temperature conditions. Boxplots indicate the median, upper and lower quartiles, and 95% confidence intervals, for values of elevational change across all mountain slopes, based on the ensemble mean of five climate models. (**B**) Predicted pattern of the seasonal difference in richness for the Eastern Himalayas mountain slope (see fig. S6), under current (black) and future (purple) temperature conditions. The latter is based on future temperature projection using climate model MPI-ESM1-2-HR.

In contrast, for most mountain slopes in our dataset, predictions from our model testing the energy efficiency hypothesis matched well empirical patterns. Along elevational gradients, we quantified the seasonal difference in richness, calculated as avian richness during the boreal summer minus avian richness during the boreal winter (Fig. 3): This diversity pattern is particularly well suited for capturing the seasonal migratory movement of the avifauna (*12*, *20*). Model predictions are significantly correlated with empirical patterns (linear mixed-effect model using all the mountain slopes in our dataset: fixed-effect slope = 0.84, *P* < 0.001, marginal *R*^2^ = 0.328, conditional *R*^2^ = 0.933). Across all the mountain slopes in our dataset, the median correlation between the simulated and observed patterns of seasonal difference in richness was 0.50, with 28 of 34 mountain slopes (82.4%) having a positive correlation (Fig. 4B). The SEDS model also performed substantially better than a null model randomizing the seasonal distribution of bird populations along elevational gradients (null-model linear mixed-effect model: fixed-effect slope = −0.01, *P* = 0.87; observed-simulated correlation distribution for SEDS is significantly skewed toward higher values than the null model: two-sample K-S test: *D* = 0.529, *P* < 0.001; fig. S2). In addition, the SEDS model captured qualitative features of the empirical patterns very well. Specifically, the model broadly predicts how the following three key features of the seasonal difference in richness pattern vary with latitude (Fig. 5): (i) boreal summer richness peak (i.e., peak in positive values of the seasonal difference in richness along the elevational gradient; Fig. 3), (ii) boreal winter richness peak (i.e., peak in negative values of the seasonal difference in richness along the elevational gradient; Fig. 3), and (iii) the transition point between elevations that have a net richness surplus during the boreal winter and a net richness surplus during the boreal summer (Fig. 3). Last, the model predicts a mix of populations tracking their thermal niches across seasons better and worse than if they stayed sedentary (Fig. 4C), with a variation that appears more realistic than the prediction from the macroevolutionary niche conservatism hypothesis (i.e., nearly all altitudinal migrants should seasonally track thermal conditions better than if they did not move; see Fig. 2B for the empirical distribution). Overall, these results suggest that the seasonal distribution of birds in mountains is largely shaped by species minimizing energetic costs while maximizing energy acquisition given what competitors are doing and that altitudinal migration is a behavioral mechanism allowing birds to optimize their energy budgets in the face of seasonality and competition. This provides an explanation for the many populations that appear to engage in upslope migration during the colder season, as a distributional strategy that is energetically efficient for some species given the dynamics of competition for access to resources. Furthermore, the predictive capacity of this model allows to predict the seasonal difference in richness along elevational gradients for which we do not have sufficient data (such as Eastern Himalayas shown in Fig. 6B).

Six mountain slopes had a negative correlation between simulated and observed patterns, all of them nonsignificant (Fig. 4B). For five of these mountain slopes, four located in temperate regions (Blue Ridge, Cascade East and West, and Rocky Mountains N) and one located in the equatorial tropics (Colombia Pacific), the model accurately predicts a seasonal difference in richness that peaks in the local summer and winter, respectively, but with a mismatch in the specific elevational location of these seasonal peaks (fig. S3). The other mountain slope with a negative correlation between simulated and observed patterns is Serra de Mantiqueira. This mountain slope and the big island of Hawaiʻi (which also has a relatively limited correlation of 0.15 and is located at a similar distance to the equator) are atypical in our dataset because they both receive noticeably few long-distance migrants, being both away from major bird migration flyways—in Hawaiʻi, 89.6% of species in our dataset are resident compared with only 44.9% in Jalisco (nearest mountain slope at similar latitude); and in Serra de Mantiqueira, 84.6% of species in our dataset are resident compared with only 54.2% in Argentina North (nearest mountain slope at similar latitude). Long-distance migrants are therefore not coming as much to these mountain slopes to exploit seasonal surpluses of energy supply. In turn, this could affect the seasonal distribution and altitudinal migration behavior of residents, thus generating patterns of seasonal difference in richness that depart from model predictions. As eBird is expanding and the quantity of checklists is rapidly increasing, this analysis could be extended to include more mountain ranges in the future, particularly covering global gaps like East Africa, Western Asia, and Southeast Asia, and to include intraspecific variation in abundance along elevational gradients, thus allowing further examination into why some mountain slopes are not well explained by the model based on the energy efficiency hypothesis.

It has long been debated whether environmental conditions and biodiversity change with elevation in the same way as they change with increasing latitude (*14*, *22*, *23*). We found that energy efficiency appears to drive both the seasonal distribution of birds across latitudes (*12*) and along mountain slopes (Figs. 4 and 5). This suggests that elevational gradients in avian distributions might be a condensed version of corresponding latitudinal gradients. It also suggests that altitudinal migration plays the same role as long-distance latitudinal migration in allowing birds to optimally exploit the energy supply along environmental gradients. The similarity between patterns along latitude and elevation allows us to directly compare them. We found a significant negative relationship between latitude and the elevation of the transition point between net richness surpluses during the boreal winter and summer across the mountain slopes in the Northern Hemisphere (${\mathrm{tr}}_{e}$), with the formula: ${\mathrm{tr}}_{e}=5070-115\text{lat}$ (*P* < 0.001, *R*^2^ = 0.908; Fig. 5A). This indicates that moving 115 m along the elevational gradient corresponds to moving 1° along the latitudinal gradient in terms of the seasonal difference in avian richness. This estimate is also obtained by contrasting the mean distance between the transition and seasonal peaks in richness (i.e., a spatial rate of change in the pattern) along elevation versus latitude (for the boreal winter peak: 144.6 m, for the boreal summer peak: 79.2 m, mean = 111.9 m of elevation per degree of latitude). Using this formula, it is possible to project the patterns of seasonal difference in richness along elevation onto the latitudinal gradient, which shows that the seasonal distribution of birds in mountains are condensed versions of the latitudinal pattern (Fig. 5C).

By evaluating how model predictions change when the cost of thermoregulation varies, it is possible to better understand the role of physiological adaptation to thermal conditions in shaping the diversity patterns of birds in mountains. We found evidence for broad physiological tolerance of birds to thermal conditions, with model parameters associated with little physiological adaptation to low temperatures (i.e., high energetic cost when temperature is low) performing substantially worse than intermediate and pronounced levels of adaptation in capturing the empirical patterns (fig. S4). Our results thus support the hypothesis that birds tend to have broad fundamental niches with relatively wide physiological tolerance to climatic conditions, and what is primarily shaping species’ realized niches is ecological interactions related to energy consumption and competition. When projecting the model predictions under the worst-case scenario for climate change for the end of the century (2071 to 2100), only accounting for change in temperature and assuming that the other environmental and energetic factors remain the same, we found that the patterns of seasonal difference in richness remain very similar to those predicted under current climate even under the most extreme climate change scenario (fig. S5). This result highlights the limited direct effect of temperature on the seasonal distribution of birds in mountains, with climate warming not expected to have a major direct effect on how birds collectively distribute along elevational gradients. However, while the patterns of seasonal difference in richness are not predicted to change much under a direct effect of temperature change, we nonetheless found a small but significant tendency for these patterns to shift upslope under future climate (Fig. 6), with boreal summer peaks shifting on average 139.4 m, boreal winter peaks shifting on average 56.9 m, the transition point between elevations that have a net richness surplus during the boreal winter and a net richness surplus during the boreal summer shifting on average 202.8 m (Fig. 6A), and with an average shift of all these features combined together of 129.5 m (one-sample *t* test: *t* = 4.283, *P* < 0.001). Patterns of species upslope shifts are already being widely reported (*24*–*27*), with Chen *et al.* (*24*) estimating a median shift of species distributions to higher elevations of 11 m per decade, which is similar to the estimate found in (*26*). This estimate would produce a median upslope shift of 83.6 m between 2009 and 2085 (i.e., the mean years of the current and future climate projections that we used in our analysis), which is in line with our estimates albeit smaller potentially because we used a worst-case scenario for projected climate change. These results indicate that species’ upslope shifts, which are often assumed to be a consequence of species tracking their climate niche, are predicted by the energy efficiency hypothesis under warmer temperatures as a result of changing energy balance and optimal distributions due to lower thermoregulation costs at higher elevation. More research is now needed to investigate how different factors, including precipitation and indirect effects of temperature, drive the energy supply available to birds and its change through time, as energy supply is the ecological component that is most likely to substantially affect the distribution of birds along elevational gradients.

In this study, we used the natural replication of elevational gradients across mountain regions, with seasonality as a natural experiment, to improve our understanding of the processes driving the distribution of avian biodiversity along environmental gradients. We show that the role of climate is not as straightforward as species simply tracking narrow climatic niches. Instead, climate is one of the factors affecting the energy balance of species, directly via thermoregulation but also potentially indirectly by affecting resource supply. Birds then distribute along elevational gradients in a way that optimizes energy balancing, which leads to some populations engaging in altitudinal migration. In this way, altitudinal migration is a behavioral mechanism that fulfills the same ecological function as long-distance latitudinal migration. Our results thus support the hypothesis that ecological dynamics and current environmental conditions are the predominant forces shaping how birds distribute in mountains. In line with (*3*, *28*), our findings are consistent with mountain avian species having relatively broad fundamental niches, with their realized niches largely shaped by resource availability and competition. Macroevolutionary dynamics and climate history still play an important role in determining the species pool of a mountain range (*14*), but our results suggest that most mountain slopes are currently near equilibrium in terms of species accumulation and now act as “ecological arenas” with specific species distributions predominantly determined by ecological dynamics. Change in the distribution of energy supply, which tends to be heterogeneous with pronounced habitat loss due to human activity at lower elevation and more protected habitat in less accessible ecosystems at higher elevation, is likely to substantially reshape how birds distribute in mountains in the Anthropocene.

## MATERIALS AND METHODS

### Mountain range data

The geographic boundaries of 219 mountain ranges were obtained from Natural Earth 1:10m Physical Labels dataset v. 5.0.0. To avoid redundancy between overlapping mountain ranges, we applied the following set of filters:

1) We kept the polygon “ATLAS MOUNTAINS” and removed the following small polygons that were contained in it: “Anti Atlas,” “ATLAS SAHARIEN,” “Atlas Tellien,” “Er Rif,” “HAUT ATLAS,” “Moyen Atlas.”

2) We removed the polygon “ROCKY MOUNTAINS” as it was very large and encompassed many other mountain range polygons.

3) We removed the polygon “Transylvanian Alps” as it was encompassed by the polygon “CARPATHIAN MOUNTAINS.”

4) We removed the polygon “Central Highlands” as it was encompassed by the polygon “CHAINE ANNAMITIQUE.”

5) We removed the polygon “Klamath Mts.” as it was encompassed by the polygon “COAST RANGES.”

6) We removed the polygons “Maoke Mts.” and “Owen Stanley Ra.” as they were both encompassed by the polygon “NEW GUINEA HIGHLANDS.”

7) We removed the polygon “Ruwenzori Range” as it was encompassed by the polygon “MITUMBA MTS.”

8) We split the large polygon “HIMALAYAS” at longitude 85°E, and we renamed the polygon to the east as “EASTERN HIMALAYAS” and the polygon to the west as “WESTERN HIMALAYAS.”

9) We split the polygon “ANDES” at latitude 32°S, and we renamed the polygon to the south as “SOUTHERN ANDES” and removed the polygon to the north as it was very large and encompassed many other mountain range polygons.

We additionally included three mountain ranges from other sources, which are globally important and particularly rich in bird data: Hawaiʻi (using the polygon for the big island of Hawaiʻi), Taiwan (using the polygon for the main island of Taiwan), and the Cordillera Centroamericana Sur located between Costa Rica and Panama. The geographic boundaries for the Cordillera Centroamericana Sur were acquired from the Global Mountain Biodiversity Assessment’s Mountain Inventory v. 1.2 (*29*), which we cropped west of 80°W longitude and south of 11°N latitude.

For each mountain range, elevation data at 3–arc sec resolution were extracted from NASA Shuttle Radar Topography Mission V003. These data were aggregated to a resolution of ~2 km to better match the resolution of checklist observations and environmental variables (see details below). This elevation dataset is available between 56°S and 60°N latitude, so mountains outside this latitudinal range were excluded from the analysis. To ensure a sufficiently wide and homogeneous elevational gradient, we removed mountain ranges with no elevation data below 600 m and/or no elevation data above 2000 m and with less than 10% of area above 1000 m. After applying these filters, 87 mountain ranges remained for analysis (fig. S7).

### Delineating mountain slopes

Mountains affect local climate, particularly as they block prevailing winds and create rain shadows, which affects the distribution of resources along mountain slopes. To account for this, as this might affect patterns of altitudinal migration of birds, we developed a methodology for delineating mountain slopes by splitting mountain ranges depending on the location of the mountain crest. Some mountain range polygons only encompass one slope of the mountain range, and therefore, these were not split further.

The method for delineating mountain slopes is illustrated in fig. S8 and used the following steps:

1) Step 1: Separate the mountain range into 0.25° bins along the direction of elongation (e.g., latitudinal bins for an east-west split of a mountain range latitudinally elongated).

2) Step 2: For each bin, plot elevation against the perpendicular coordinates (e.g., longitude for a latitudinal bin) and fit a smoothing spline with a smoothing parameter equals to 0.5.

3) Step 3: For each bin, compute the coordinates of the mountain crest as (i) the mean coordinate for the bin along the direction of elongation (e.g., mean latitude for latitudinal bins) and (ii) the mode of the smoothing spline for the perpendicular coordinates (e.g., longitude for latitudinal bins)

4) Step 4: Generate a spatial line connecting the mountain crest of every bin along the direction of elongation of the mountain range and split the polygon using that spatial line.

To maximize environmental homogeneity and standardize mountain slopes, we cropped each of them to be 2° wide. The specific location of where a mountain slope was cropped from a mountain range was chosen to optimize the balance between broadest topographic extent and largest availability of bird data (see section below). Figures S9 to S34 show the resulting mountain slopes within their respective mountain ranges.

### Bird data

Data on the distribution of birds were obtained from the participatory science program eBird (accessed April 2024). We extracted eBird checklists conducted between 2002 and 2024 and located within the polygons of mountain ranges. Following eBird best practices (*30*), we filtered the eBird dataset for each mountain slope by including only complete checklists collected using protocol P21, P22, or P23, which were (i) either stationary, traveling 2 km or less or with an effort area of <100 ha (to reduce the uncertainty related to the altitude of the checklist); (ii) with a duration <5 hours; and (iii) done by 10 observers or less. Checklists were then categorized as boreal summer or boreal winter checklists or neither. Boreal summer checklists were obtained between 1 June and 15 August, and boreal winter checklists were obtained between 1 December and 15 February. These temporal ranges were used as a compromise between maximizing the number of checklists and minimizing the risk of including birds that are on migration.

Using species trait data from the AVONET dataset (*31*), we removed species associated with the aquatic environment from the eBird checklists, as these species are typically associated with lowland habitats and their resource supply is substantially different from the other species and might not be easily estimated using the same proxies. Specifically, we removed species with (i) habitat: coastal, marine, riverine, and wetland; (ii) primary lifestyle: aquatic; or (iii) trophic niches: aquatic predator and herbivore aquatic. We also removed species with habitat category human modified, as these species might not respond to seasonal change in natural habitat in the same way as other species.

Each eBird checklist is provided with longitude and latitude coordinates, and we used these coordinates to extract elevation using the same elevation raster layer we used to filter mountain ranges (see section above). To ensure sufficient data availability, and over a wide elevational gradient, we retained mountain ranges with at least 1000 checklists during both seasons and a difference between the lowest and highest checklists of at least 1500 m during both seasons. We retained mountain slopes (i.e., 2° crops of mountain ranges) with at least seven 200-m elevation bins containing at least 10 checklists at each season. We then calculated sampling completeness for each 200-m elevation bin within each mountain slope as (observed species richness)/(Chao2) following (*32*). A metric of seasonal sampling completeness was then calculated as the median sampling completeness for elevational bins within each mountain slope at each season (i.e., boreal summer and winter). Mountain slopes ranged in this metric of seasonal sampling completeness from 0.61 to 0.95. We removed from the dataset mountain slopes with at least one seasonal sampling completeness smaller than 0.70, to ensure that species’ elevational ranges could be adequately defined using available eBird data. After applying these filters, 34 mountain slopes remained for analysis (Fig. 1).

### Seasonal elevational ranges of populations

To estimate population-level seasonal elevational ranges, we adapted the methodology from (*28*), which uses a frequency approach that takes into account variation in sampling intensity along mountain slopes during each season. For a given species along a given mountain slope (hereafter referred to as a population) during a given season, we first calculated the fraction of checklists in which the species was observed for each 200-m elevational bin. To account for checklist-level survey effort for this calculation, the contribution of each checklist was weighted by its associated number of observers and duration, with the checklist weight equals to the number of observers multiplied by the observation duration (i.e., the total observation time across all observers). The maximum detection frequency (i.e., elevational bin with the highest fraction of checklists in which the species was observed) was rescaled to 1. We then defined the lower and upper boundaries of the seasonal elevational range as the lowest and highest elevational bins in which the species observation frequency was higher than 0.05 (Fig. 3). This threshold value for delineating range limits was taken from (*28*), but we also ran a sensitivity analysis using two alternative values for this threshold (0.01 and 0.1) which led to very similar results (fig. S35). We only estimated seasonal elevational ranges for species observed in at least 20 checklists over the entire mountain slope, and we only used elevational bins with at least 10 checklists to minimize the effect of detection stochasticity. This procedure resulted in a dataset of 10,998 population-level seasonal elevational ranges across our mountain slopes (figs. S36 to S69). We also calculated population’s seasonal mean elevation as the mean of elevational bins weighted by the corresponding observation frequencies, and altitudinal migration distance was calculated as the absolute difference between seasonal mean elevations for species occurring year-round in a mountain slope.

### Seasonal temperature difference

To estimate population-level seasonal tracking of thermal conditions, we first extracted seasonal temperature for each 200-m elevational bin. Mean monthly temperature (in degrees Celsius) data were obtained from CHELSA version 2.1 at ~1-km resolution (*33*) between 2000 and 2018. Seasonal temperature was obtained by averaging the data for each 200-m elevational bin over the boreal summer (April to September) and boreal winter (October to March) and over the 19 years of climate data. We divided the annual cycle into boreal summer and boreal winter because it corresponds to the global timing of seasonality due to the way Earth rotates around the Sun, i.e., yearly orbit with a tilted axis of rotation. Temperature and precipitation, which dominate seasonality patterns in temperate and tropical regions, respectively, both follow similar seasonal cycles that can be broadly divided into times of year corresponding to the boreal summer and the boreal winter.

We averaged climate over a multiyear period to best capture the predictable seasonal variation in climate that birds are susceptible to track. For each population (i.e., a given species along a given mountain slope), at each season, we calculated the mean temperature across the elevational bins weighted by the rescaled species observation frequencies (see the “Seasonal elevational ranges of populations” section). Seasonal temperature tracking was then calculated as the absolute difference between the weighted mean temperatures during the boreal summer and winter.

### Empirical seasonal diversity patterns along elevational gradients

For each mountain slope and at each season, species richness was calculated for each 200-m elevational bin as the number of species whose seasonal elevational range overlapped the elevational bin. We then quantified for each mountain slope the pattern of seasonal difference in richness, by calculating for each elevational bin: richness during the boreal summer minus richness during boreal winter (Fig. 3). We fitted a smoothing spline with a smoothing parameter equals to 0.5 and extracted for each mountain slope the elevations of the boreal summer mode and boreal winter mode, if they existed (Fig. 3). We also extracted the elevation of the transition point between elevations that are net receivers of migratory species during the boreal winter and net receivers of migratory species during the boreal summer, if such transition point was present (Fig. 3).

### Global seasonal diversity patterns along latitudinal gradients

We quantified the global pattern of seasonal difference in avian richness using the methodology from (*34*). Briefly, we used a global dataset of spatial polygons representing the global distribution of 9783 nonmarine bird species (*35*), to quantify avian richness during the boreal summer and boreal winter across a global grid of equal-area, equal-shape hexagons [~23,322 km^2^; (*36*)]. The data and their treatment are described in detail in (*34*). We then estimated the qualitative features of the pattern, i.e., boreal winter peak, boreal summer peak, and the transition between elevations that are net receivers of migratory species during the boreal winter and net receivers of migratory species during the boreal summer, following the same method as for the patterns along elevational gradients (see the “Empirical seasonal diversity patterns along elevational gradients” section), for each of the global flyways delineated as follows: Americas Flyway (>30°W longitude), Europe-Africa Flyway (30°W to 60°E longitude), and the East Asia–Australasia Flyway (>60°E longitude).

### SEDS: Model overview

To simulate the seasonal distributions of birds along a mountain slope, we adapted the SEDS model, which was originally used to model the global seasonal distribution of birds (*12*). Here, the model is applied to a bipartite network whose nodes represent elevational bins along the mountain slope during two seasons (boreal summer and boreal winter), with also one additional node per season to accommodate long-distance migrants going in and out of the mountain slope (fig. S70). The SEDS model is a mechanistic model based on first ecological and energetics principles, relating the energy supply in the environment with the energy requirements of bird species. This model is agnostic to species differences in terms of morphology, physiology, or biology, effectively assuming ecological, demographic, and energetic equivalence between species. This simplifying assumption is not intended to correspond to reality but to test how far a model can go without the need to integrate interspecific variation in flight ability, thermoregulation, or reproductive strategies.

The model is applied through a sequence of simulation steps whereby a virtual mountain slope is progressively filled with virtual bird populations until it becomes saturated. A model simulation starts with a virtual empty mountain slope with the same geography and seasonality as the real one (i.e., similar distribution of land area in the elevational bins and similar seasonal climate and primary productivity). For each simulation step, a local avian population, which occupies only one node per season, is selected among all options (i.e., all combinations of seasonal nodes) as being the most energy efficient (i.e., resulting in the most favorable cost-benefit balance). Once the selected bird population is added to the virtual mountain slope, the energy supply in the elevational bins seasonally occupied by that population is depleted according to the energy consumed by that population. The model is not an evolutionary model of competition, taking into account neither the potential mortality associated with the processes nor the potential increases in reproductive output under favorable conditions. Instead, the model essentially assumes species’ populations to be at demographic equilibrium, and it aims to simulate the energetically optimal distribution of avian populations along mountain slopes under equilibrium conditions.

### Energetic costs

The energetic requirement of each bird population is composed of the basal energy use for existence, which is set to be 1 (arbitrary) unit, and additional costs associated with reproduction, Cr, thermoregulation, Ct, and migration, Cm, which were converted into the arbitrary units of energy use.

The energetic cost associated with reproduction during the boreal summer, ${\mathrm{Cr}}_{s}$, and during the boreal winter, ${\mathrm{Cr}}_{w}$, was estimated as

$$\left\{\begin{array}{c} {\mathrm{Cr}}_{s}=r\cdot P_{r}\\ {\mathrm{Cr}}_{w}=r\cdot \left(1-P_{r}\right) \end{array}\right.$$

where r is the energetic cost of reproduction relative to the basal metabolic rate, which was set to r=0.41 following the best-fit estimation from (*12*); and $P_{r}$ is the probability of breeding during the boreal summer, which was set to $P_{r}=1$ for all mountain slopes at latitude > 15°N latitude, $P_{r}=0.75$ for mountain slopes between 0° and 15°N latitude, $P_{r}=0.25$ for mountain slopes between 0° and 15°S latitude, and $P_{r}=0$ for all mountain slopes at > 15°S latitude.

The energetic cost associated with thermoregulation, Ct, corresponds to the additional energetic cost of maintaining a relatively constant internal body temperature for endotherms like birds, and it was estimated for elevational bin i at a given season following (*12*) as

$$\left\{ \begin{array}{cc} {\mathrm{Ct}}_{i}=\frac{40-T_{a}-\beta }{\beta } & ,\text{if}\;T_{a}<40-\beta \\ {\mathrm{Ct}}_{i}=0 & ,\text{if}\;T_{a}\geq 40-\beta \end{array}\right.$$

where $T_{a}$ is the mean ambient temperature across elevational bin i at that season, and β is a parameter that dictates the lower critical temperature of the thermoneutral zone of an average bird (i.e., 40 − β), which was set to β = 23.6 based on the best-fit estimation from (*12*). To evaluate the sensitivity of the model predictions to change in the cost of thermoregulation, we ran the SEDS model with values for β∈{5,10,…,45}.

Last, the energetic cost associated with seasonal local movement between elevational bins was set to Cm=0, assuming no energetic cost of local altitudinal migration because of the short distances involved. We ran a sensitivity analysis adding a small energetic cost for altitudinal migration, using the estimate from (*12*): $\mathrm{Cm}=6.45\times 10^{-5}d$, with d the migration distance that we estimated to be 5 km to move between adjacent 200-m elevational bins along the elevational gradient of a mountain slope. The results we obtained by including an additional cost of altitudinal migration, which was very small compared with the other costs, were identical to the results obtained assuming no energetic cost of altitudinal migration.

### Energy supply

In each elevational bin along a mountain slope, the energy supply indicates the total amount of resources that is used to fuel bird population metabolism during a particular season. We modeled the energy supply as being proportional to the normalized difference vegetation index (NDVI), a remotely sensed measure of greenness that correlates well with primary productivity and has been often used in studies examining the effect of energy and resources on the distribution of birds (*12*, *37*–*39*). NDVI data, ranging between 0 and 1, were obtained per 10-day periods from Copernicus Land Monitoring Service at a spatial resolution of 1 km and averaged over the period of 1999 to 2017. Energy supply in elevational bin i during the boreal summer was estimated as ${\mathrm{Es}}_{i}=\text{NDVI}s_{i}\cdot {\text{area}}_{i}$ and during the boreal winter as ${\mathrm{Ew}}_{i}=\text{NDVI}w_{i}\cdot {\text{area}}_{i}$, with $\text{NDVI}s_{i}$ and $\text{NDVI}w_{i}$ indicating the mean NDVI values across elevational bin i between April to September and October to March, respectively, and with ${\text{area}}_{i}$ indicating the number of elevation pixels within elevational bin i.

### Long-distance latitudinal migrants

Long-distance migrants, migrating away from a mountain slope during part of the year and affecting the difference in total species richness between the boreal summer and boreal winter in a mountain slope, were accommodated in the model using an additional seasonal node. In a given mountain slope, the extra node was added to the season with the lowest number of long-distance migratory species, the latter being defined as species only present during one season and estimated using the empirical seasonal elevational ranges (see description above). The energy supply allocated to this extra node to accommodate long-distance migrants, $E_{m}$, was estimated for a given mountain slope as

{Em=Ss−SwSw∑i=1nEwi,if Ss>SwEm=Ss−SwSs∑i=1nEsi,if Sw>Ss

where $S_{s}$ and $S_{w}$ are the total number of species during the boreal summer and boreal winter, respectively, and n is the number of elevational bins in the mountain slope.

The latitude where long-distance migrants spend the season during which they are away from the mountain slope was estimated as the latitude of the peak in seasonal difference in richness along the corresponding global flyway, which was taken as the boreal summer peak if the mountain slope was the net receiver of long-distance migrants during the boreal winter or taken as the boreal winter peak if the mountain slope was the net receiver of long-distance migrants during the boreal summer (see the “Global seasonal diversity patterns along latitudinal gradients” section for more information on how these global seasonal peaks were estimated). Latitudinal migration distance (in kilometers) was calculated as $d=111\times |{\text{Lat}}_{\text{slope}}-{\text{Lat}}_{\text{peak}}|$, where 111 is the length of the shortest distance (in kilometers) between locations separated by 1° of latitude, ${\text{Lat}}_{\text{slope}}$is the mean latitude of the mountain slope, and ${\text{Lat}}_{\text{peak}}$ is the latitude where long-distance migrants spend the season during which they are away from the mountain slope. The energetic cost of long-distance migration was then obtained as $\mathrm{Cm}=6.45e^{-5}d$, which was estimated on the basis of flight physiology following (*12*). The temperature experienced by long-distance migrants when away was estimated as the mean seasonal temperature of pixels within 1° latitude of the latitude where they spend the season during which they are away from the mountain slope. In the Southern Hemisphere, as it is difficult to distinguish whether long-distance migrants come from the Northern Hemisphere or within the Southern Hemisphere, we assigned an arbitrary distance of *d* = 3000 and an arbitrary temperature of $T_{a}=20$ for the away node. A sensitivity analysis for these values for mountain slopes in the Southern Hemisphere shows that the model outputs are robust to the values chosen for these parameters (fig. S71).

### Simulation steps

Each simulation step consisted of calculating an energy efficiency score for all possible population distributions (i.e., pairs of summer-winter nodes), which include long-distance migration away from the mountain slope to the external seasonal node (see the “Long-distance latitudinal migrants” section above). The energy efficiency score for a given population distribution was defined as

$$E_{\text{score}}=\frac{\mathrm{Es}}{\mathrm{Cs}}+\frac{\mathrm{Ew}}{\mathrm{Cw}}$$

where Cs is the energetic cost during the boreal summer and is equal to $1+{\mathrm{Cr}}_{s}+{\mathrm{Ct}}_{s}+\mathrm{Cm}$, and Cw is the energetic cost during the boreal winter and is equal to $1+{\mathrm{Cr}}_{w}+{\mathrm{Ct}}_{w}+\mathrm{Cm}$. The population distribution selected is the one with the highest $E_{\text{score}}$ (i.e., the most energy efficient). The newly simulated population, which is defined by two elevational bins (one occupied during the boreal winter and one occupied during the boreal summer), was added to the virtual mountain slope and consumed energy in the nodes where it occurred seasonally, thus reducing the energy supply in the boreal summer node and boreal winter node by Cs and Cw, respectively. New virtual populations were added until the virtual mountain slope was nearly saturated (i.e., 95% of the energy supply is used).

### Simulated seasonal species richness patterns

Once a virtual mountain slope was simulated and was saturated with virtual bird populations, seasonal species richness S was estimated as a function of the number of simulated populations N following a species-abundance distribution logseries (*40*)

$$S=\theta \cdot \text{log}\left(1+\frac{N}{\theta }\right)$$

with θ a free parameter that was set to 100. We also assessed model results for θ = 50 and θ = 200, with both showing very similar results to θ = 100 (figs. S72 to S75). For each 200-m elevational bin, the simulated seasonal difference in richness was calculated as the number of simulated species during the boreal summer minus the number of simulated species during the boreal winter. Qualitative features of this pattern, i.e., boreal winter peak, boreal summer peak, and the transition between elevations that were net receivers of migratory species during the boreal winter and net receivers of migratory species during the boreal summer, were extracted in the same way as for the empirical patterns (Fig. 3).

### Correspondence between observed and simulated patterns

To evaluate the model, we compared the predictions for the pattern of seasonal difference in richness along elevational gradients with our empirically estimated patterns. To do so, we first used a linear mixed-effect model, with the observed seasonal difference in richness as the response variable and the simulated seasonal difference in richness and mountain slope as explanatory variables. We also calculated the Pearson’s correlation coefficient between observed and simulated seasonal difference in richness for every mountain slope separately. Last, we evaluated the relationships between the simulated and empirical qualitative features of the patterns of seasonal difference in richness (i.e., boreal summer peak, boreal winter peak, and transition) for mountain slopes in the Northern Hemisphere.

### Null model

To test whether the SEDS model performed better than random, we developed a null model that randomized the seasonal distribution of bird populations along elevational gradients. For a given mountain slope, the null model simulated the seasonal distribution of N populations, with N corresponding to the number of populations simulated by the SEDS model for this mountain slope. For each population, we randomly sampled one elevational bin during the boreal summer and one elevational bin during the boreal winter to obtain its seasonal elevational distribution along the elevational gradient. We then estimated the pattern of seasonal difference in richness in the same way as for the output of the SEDS model.

### Predicting seasonal difference in richness in Eastern Himalayas

The mountain slope was cropped longitudinally from the Eastern Himalayas mountain range between 88.2°W and 90.2°W longitude (fig. S6). As the amount of eBird occurrence data in this region is relatively high, despite being just below our completeness threshold to be included in the analysis (see the “Bird data” section), we estimated energy supply allocated to the extra node to accommodate long-distance migrants in the same way as for the other mountain slopes (see the “Long-distance latitudinal migrants” section). We then ran the adapted SEDS model and extracted a simulated pattern of seasonal difference in richness, following the same steps as for the other mountain slopes.

### Future predictions

Predictions for the seasonal difference in richness patterns along the elevational gradients of mountain slopes were obtained by using the SEDS model with the same parameter values and seasonal distribution of energy supply as described above, but with different values for seasonal temperatures in each elevational bin to reflect a scenario of future climate change. These new temperature values were obtained from CHELSA version 2.1 at ~1-km resolution (*33*) for the period of 2071 to 2100 under the CMIP6 scenario, modeled using the shared socioeconomic pathway 585 (worst-case scenario). We ran model simulations using five different climate models—GFDL-ESM4, IPSL-CM6A-LR, MPI-ESM1-2-HR, MRI-ESM2-0, and UKESM1-0-LL—and calculated the ensemble mean of the seasonal difference in richness patterns predicted by these models for each elevational bin.
