# Supplementary material for: Climate, ecological dynamics, and the seasonal distribution of birds in mountains
Source: Sci Adv. 2026 Feb 6;12(6):eadz5547. doi: 10.1126/sciadv.adz5547 (PMC12880528; doi:10.1126/sciadv.adz5547)
Supplement: Supplementary file 1 — Figs. S1 to S75 Table S1 [file sciadv.adz5547_sm.pdf]

Science Advances
AAAS

Supplementary Materials for

Climate, ecological dynamics, and the seasonal distribution of birds in mountains

Marius Somveille *et al.*

Corresponding author: Marius Somveille, marius@somveille.com

Sci. Adv. **12**, eadz5547 (2026)
DOI: 10.1126/sciadv.adz5547

This PDF file includes:

Figs. S1 to S75
Table S1

Supplementary Figures

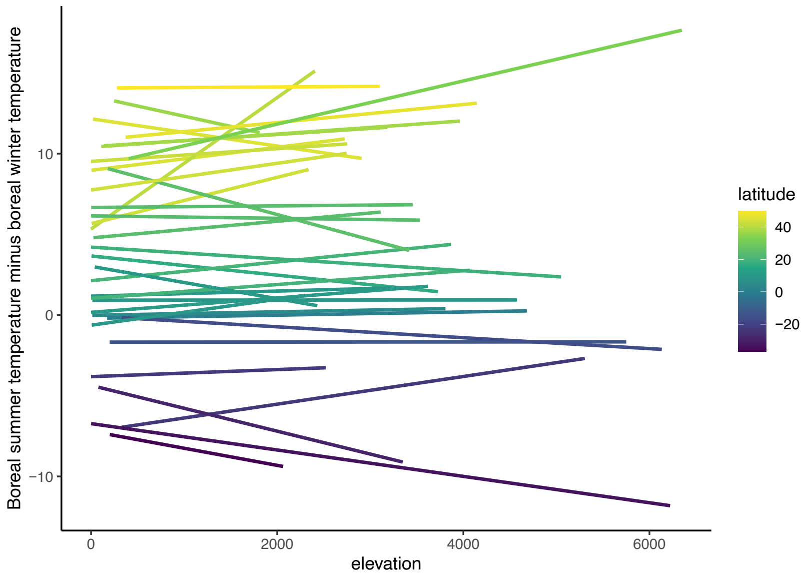

Fig. S1. Variation in temperature seasonality across elevation and latitude in our dataset. Each line indicates the line of best fit from a linear model for a mountain slope. Temperature seasonality was calculated as temperature during the boreal summer minus temperature during the boreal winter. The figure indicates that, while temperature seasonality increases with increasing latitude, it changes very little with elevation for most mountain slopes.

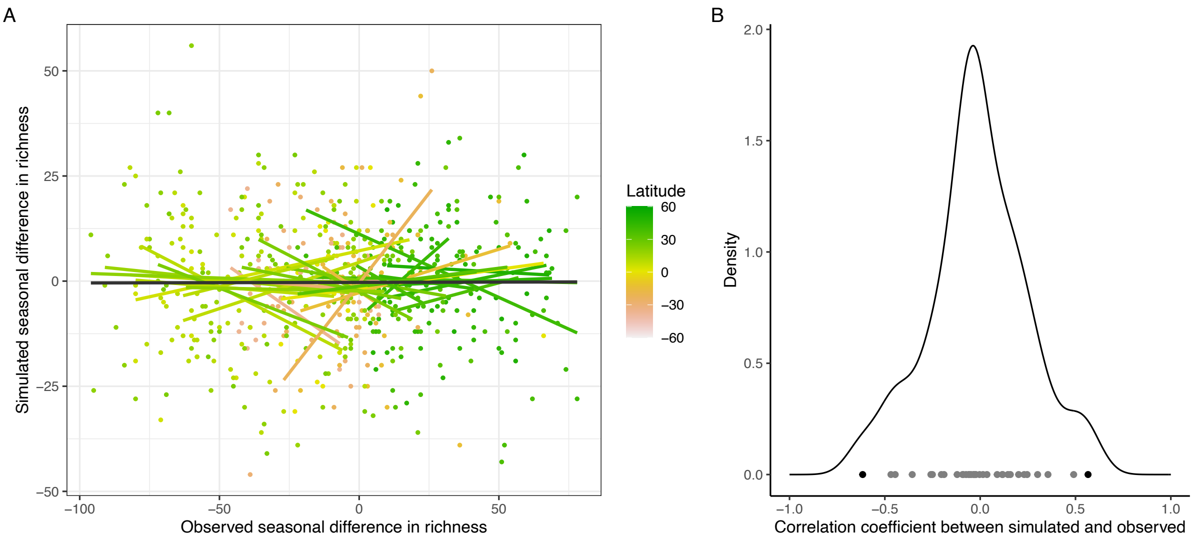

Fig. S2. Results for the null model randomising the seasonal distribution of bird populations along elevational gradients. (A) Relationship between the seasonal difference in richness simulated by the null model and estimated empirically. Each slope corresponds to a line of best fit for a mountain slope, and each point correspond to a 200m elevational bin, both colour-coded by latitude. The black line indicates the line of best fit for the whole dataset. (B) Density plot of the Pearson's correlation coefficients between null model predictions and observation for the seasonal difference in richness, for all the mountain slopes

in our dataset. Grey and black points indicate correlation coefficients that are non-significant and significant, respectively, based on correlation statistical tests.

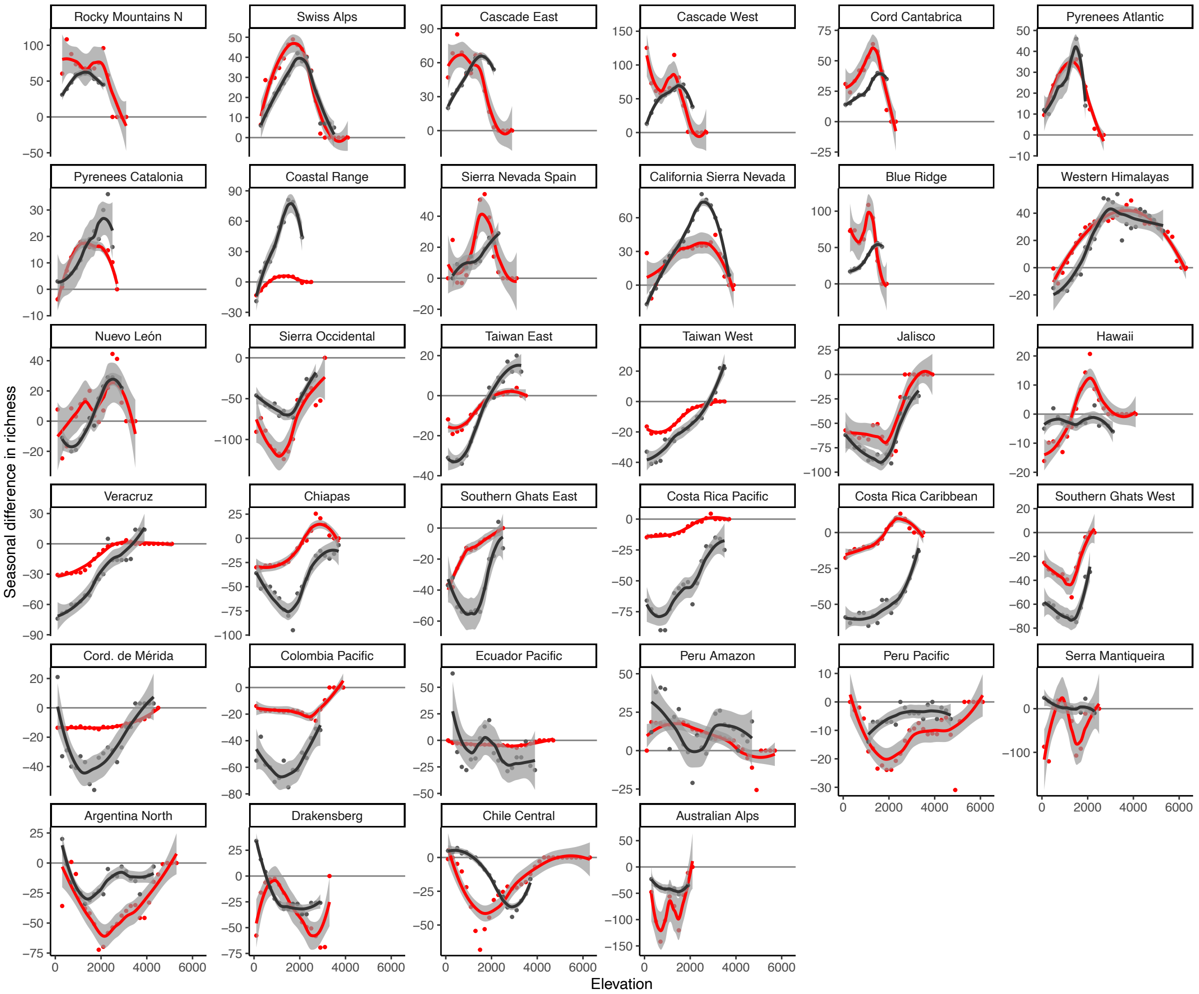

Fig. S3. Patterns of seasonal difference in richness across mountain slopes. Each panel represent a mountain slope, ordered by decreasing latitude from top to bottom. The black lines indicate smooth curves (with standard error) fitted to the empirical estimates along the elevational gradients, and red lines indicate smooth curves (with standard error) fitted to the simulated estimates along the elevational gradients.

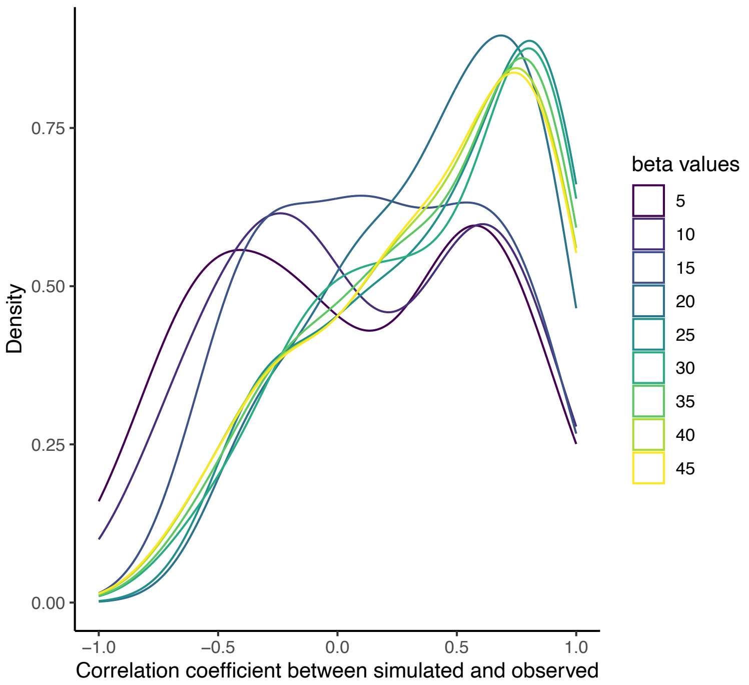

Fig. S4. Results of the sensitivity analysis for the cost of thermoregulation. Density plot of the Pearson's correlation coefficients between model predictions and observation for the seasonal difference in richness, for all the mountain slopes in our dataset. The colour scheme indicates the different values for β that were investigated. Low β values indicate little physiological adaptation to low temperatures while high β values indicate pronounced physiological adaptation to low temperatures.

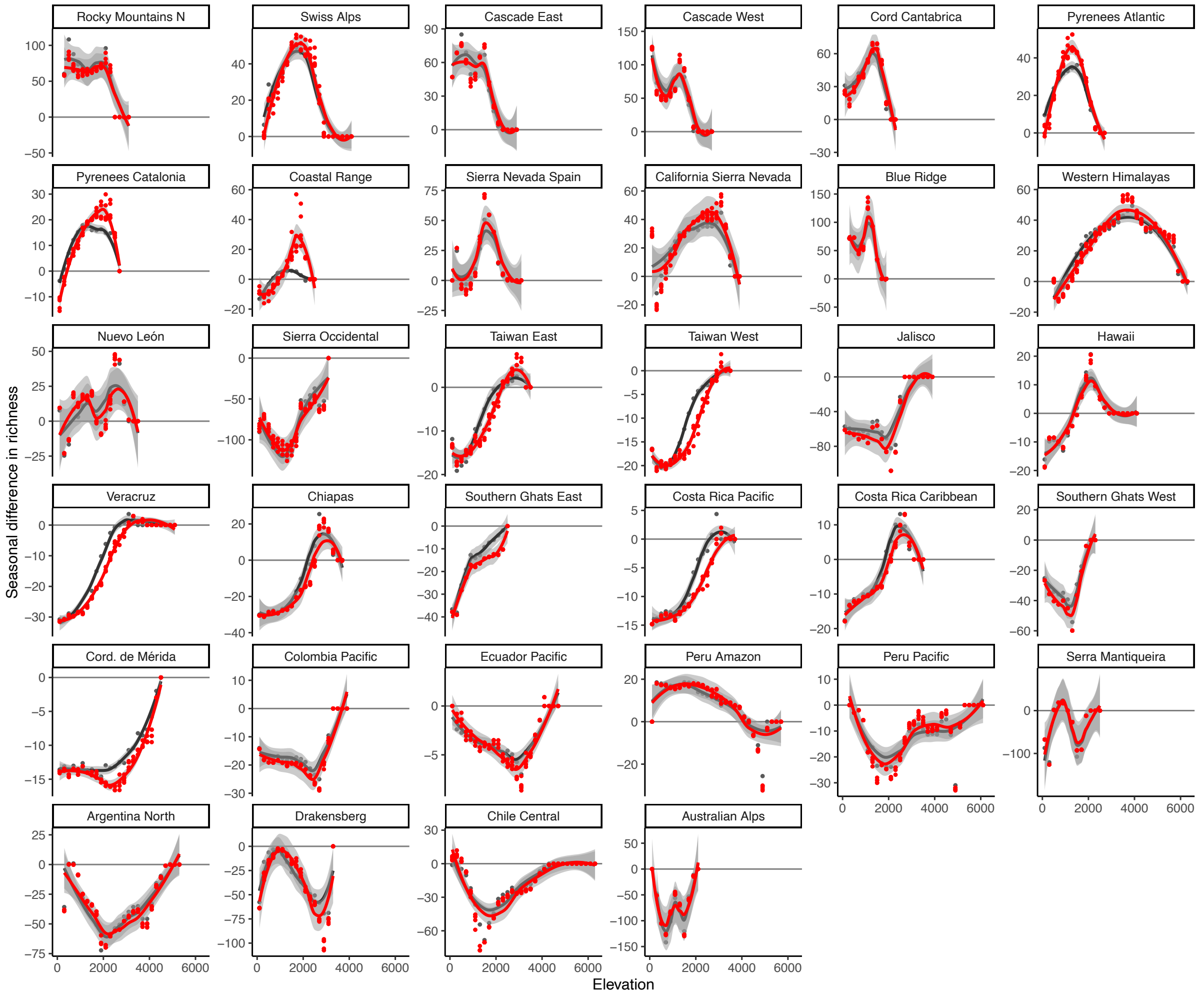

Fig. S5. Predicted patterns of seasonal difference in richness across mountain slopes under both current and future climate. Each panel represent a mountain slope, ordered by decreasing latitude from top to bottom. The black lines indicate smooth curves (with standard error) fitted to estimates simulated along the elevational gradients under current climate, and red lines indicate smooth curves (with standard error) fitted to estimates simulated along the elevational gradients under future climate (2071–2100) taken as the ensemble mean of model predictions ran for 5 different climate models. Red points indicate the model predictions based on the different climate models.

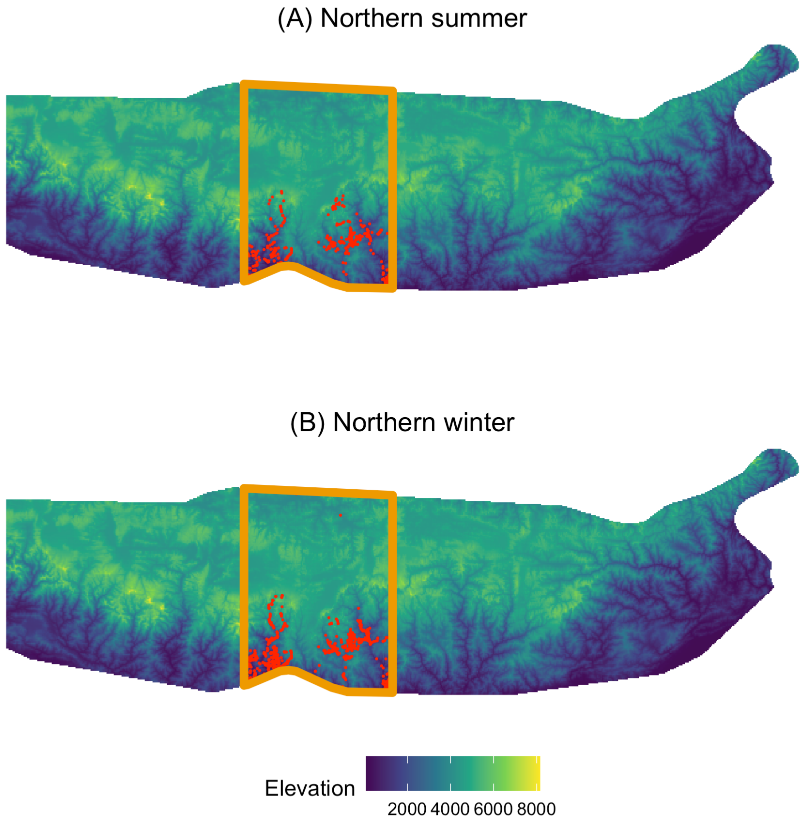

Fig. S6. Location of the *Eastern Himalayas* mountain slope within the Eastern Himalayas mountain range. The orange line delineates the mountain slope polygon. Red points indicate the location of eBird checklists during the boreal summer (A) and boreal winter (B).

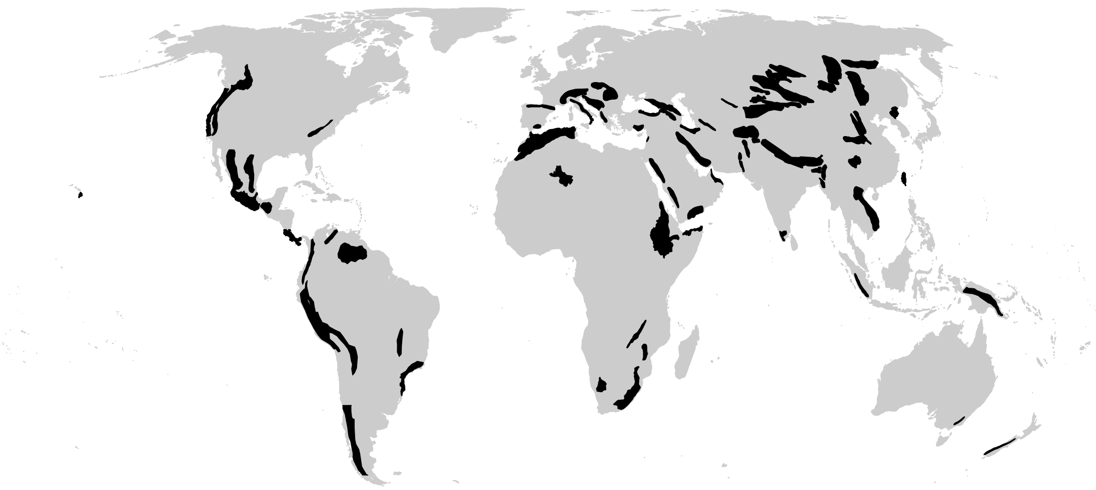

Fig. S7. The global distribution of mountain ranges used in this study. Black polygons on the map indicate the location of the 87 mountain ranges selected after the filtering steps described in the *mountain ranges data* section in the Supplementary Materials.

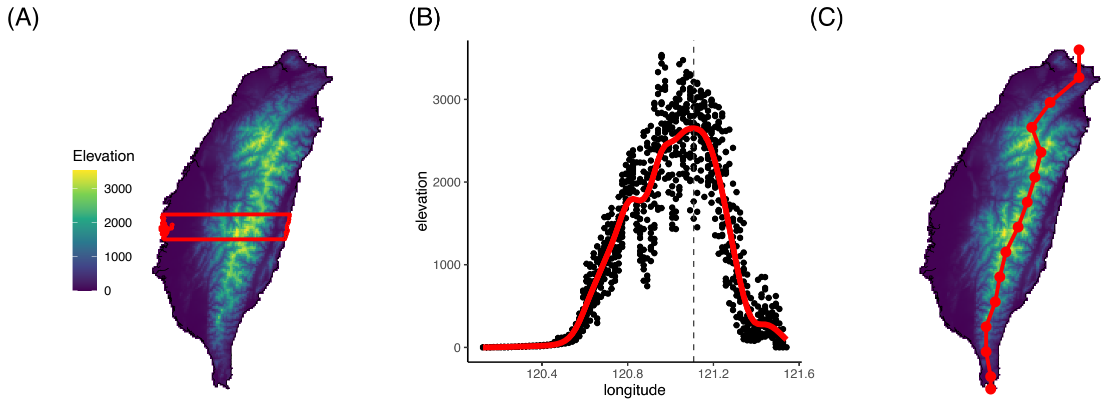

Fig. S8. Method for delineating mountain slopes. Taiwan is used to illustrate the methodology. (A) The mountain range is separated into 0.25 degrees bins along the direction of elongation. As Taiwan is elongated latitudinally, it is split into latitudinal bins. The red polygon indicates an example of a latitudinal bin. (B) The pattern of elevation is quantified along perpendicular coordinates (i.e., longitude here for Taiwan) by fitting a smoothing spline with a smoothing parameter equals to 0.5 (red line). The mountain crest for each bin is computed as the mode of the smoothing spline (dashed vertical line). (C) For each bin, mountain crests are positioned using the mean coordinate for the bin along the direction of elongation (e.g., mean latitude for each bin across Taiwan). The mountain range is split along a spatial line connecting the mountain crest of every bin. Red points indicate the mountain crest for each latitudinal bin, and the red line indicate the spatial line connecting the mountain crests which is used to split the mountain range.

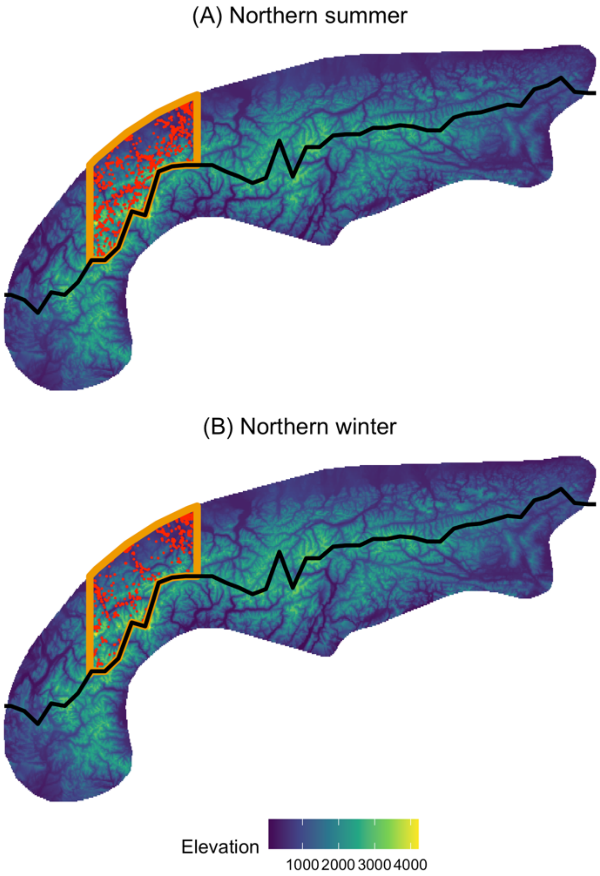

Fig. S9. Location of the *Swiss Alps* mountain slope within the European Alps mountain range. The black line indicates the spatial line splitting the mountain range, and the orange line delineates the mountain slope polygon. Red points indicate the location of eBird checklists during the boreal summer (A) and boreal winter (B). The *Swiss Alps* mountain slope was delineated to capture the area of the European Alps with the greatest elevational range, which also has good numbers of checklists during both summer and winter.

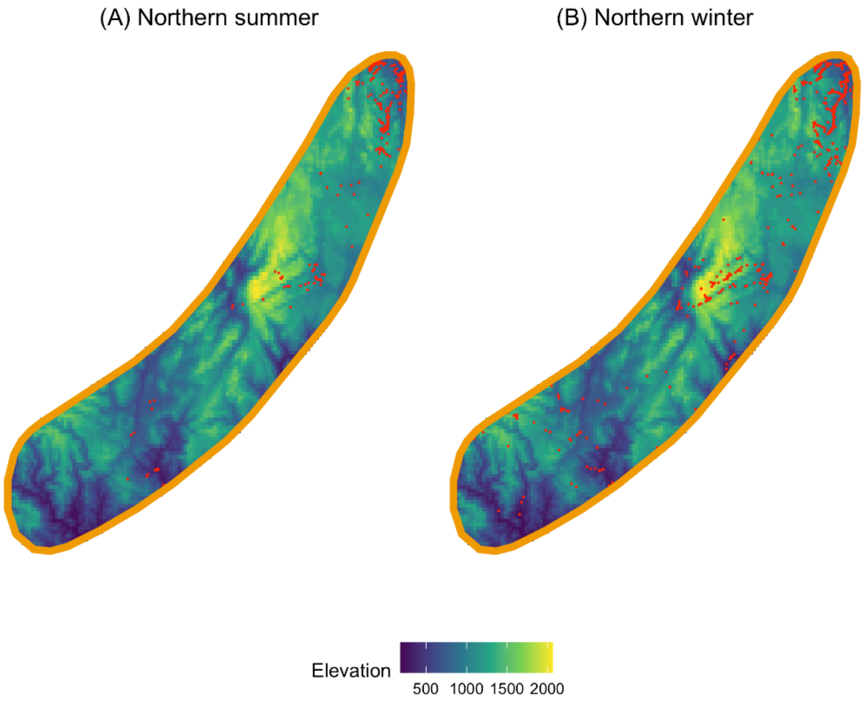

Fig. S10. ***Australian Alps*** **mountain slope.** The orange line delineates the mountain slope polygon. Red points indicate the location of eBird checklists during the boreal summer (A) and boreal winter (B).

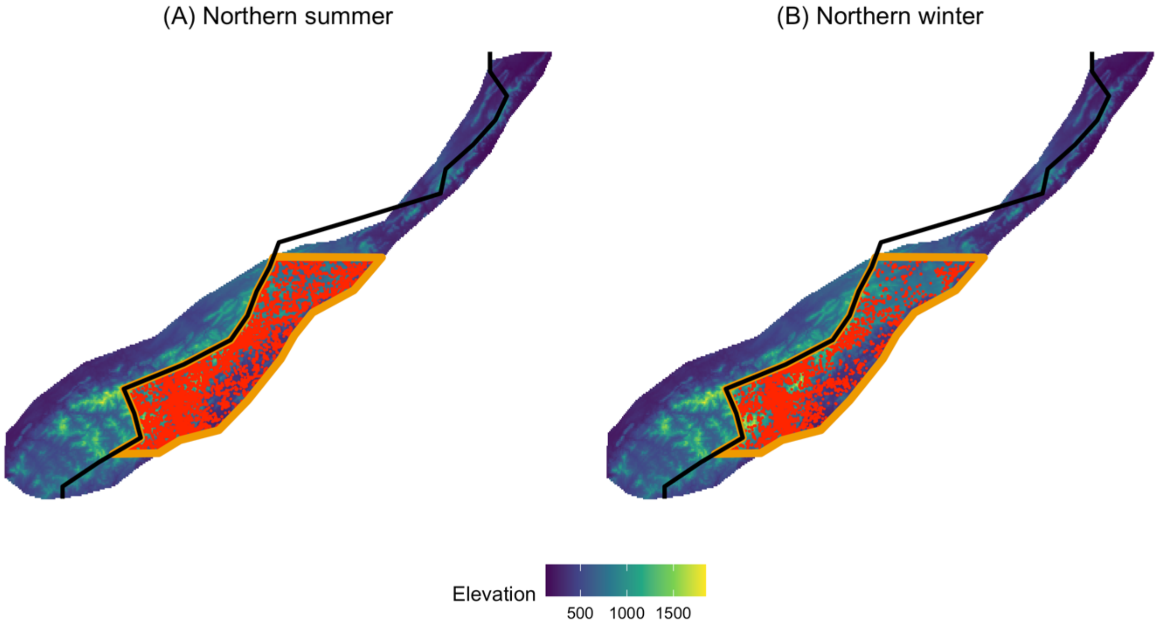

Fig. S11. Location of the ***Blue Ridge*** **mountain slope within the Appalachia mountain range.** The black line indicates the spatial line splitting the mountain range, and the orange line delineates the mountain slope polygon. Red points indicate the location of eBird checklists during the boreal summer (A) and boreal winter (B). As this mountain range does not create significantly different climatic conditions on either sides of the crests, due to its relatively low elevation, we only selected one slope based on data availability.

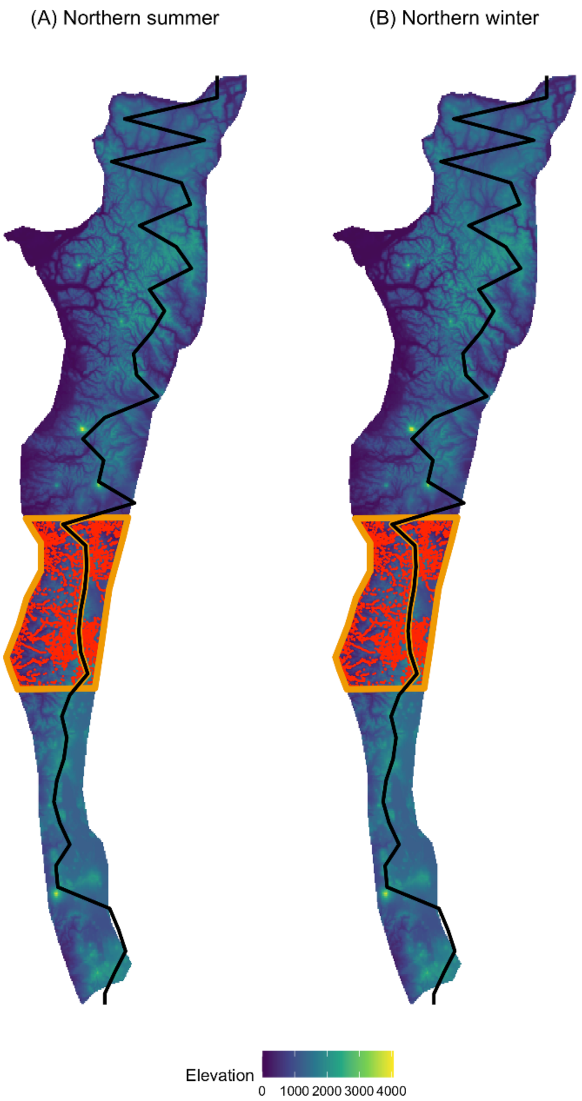

Fig. S12. Location of the *Cascade West and Cascade East* mountain slopes within the Cascades mountain range. The black line indicates the spatial line splitting the mountain range, and the orange lines delineate the mountain slope polygons. Red points indicate the location of eBird checklists during the boreal summer (A) and boreal winter (B).

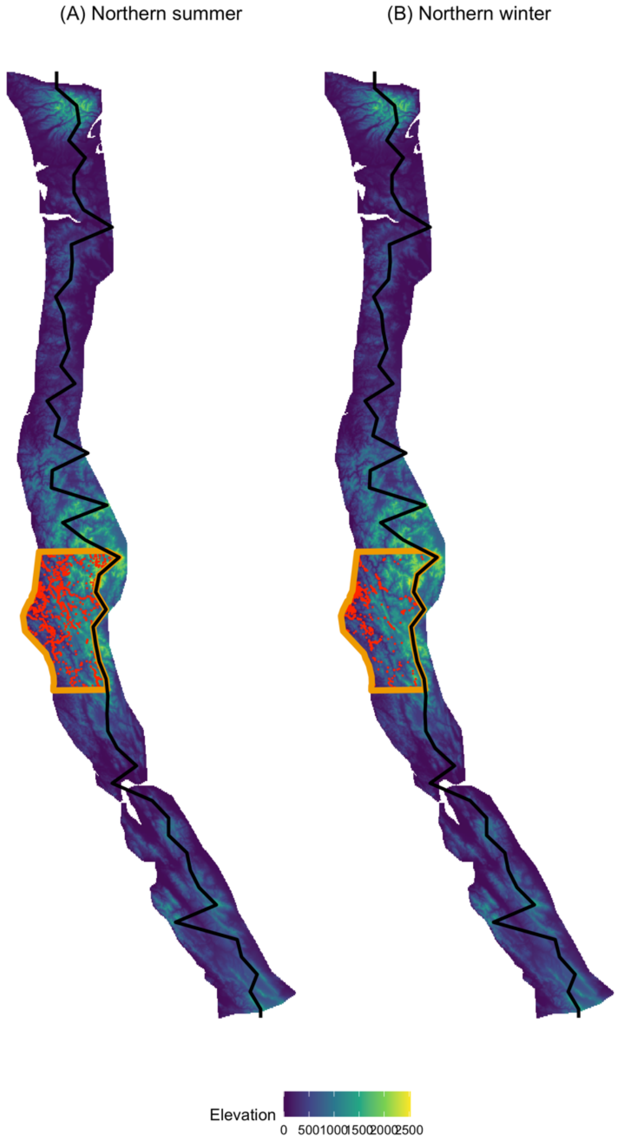

Fig. S13. Location of the *Coastal Range* mountain slope within the Pacific Coast Ranges. The black line indicates the spatial line splitting the mountain range, and the orange line delineates the mountain slope polygon. Red points indicate the location of eBird checklists during the boreal summer (A) and boreal winter (B).

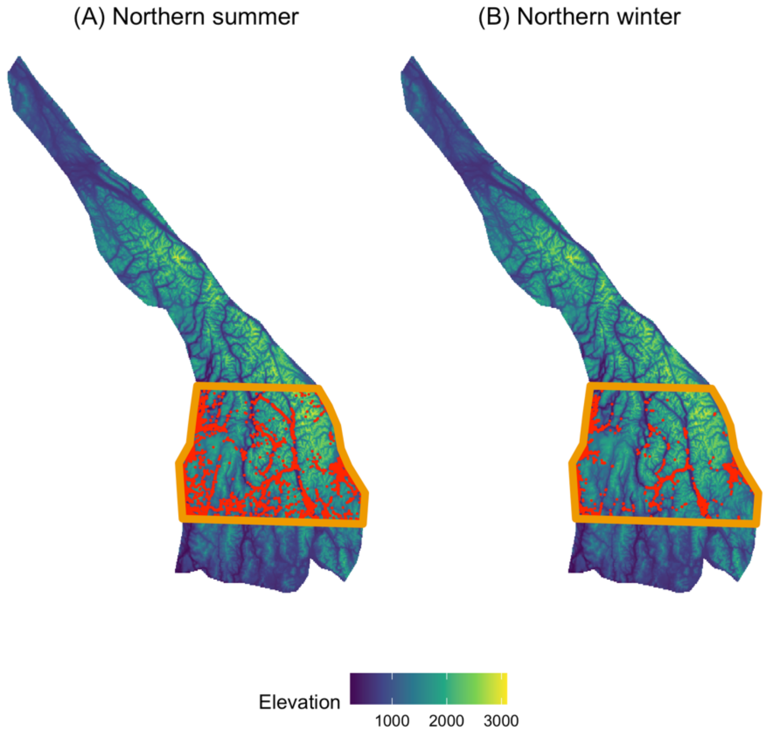

Fig. S14. Location of the *Rocky Mountains N* mountain slope within the Rocky Mountains range. The orange line delineates the mountain slope polygon. Red points indicate the location of eBird checklists during the boreal summer (A) and boreal winter (B).

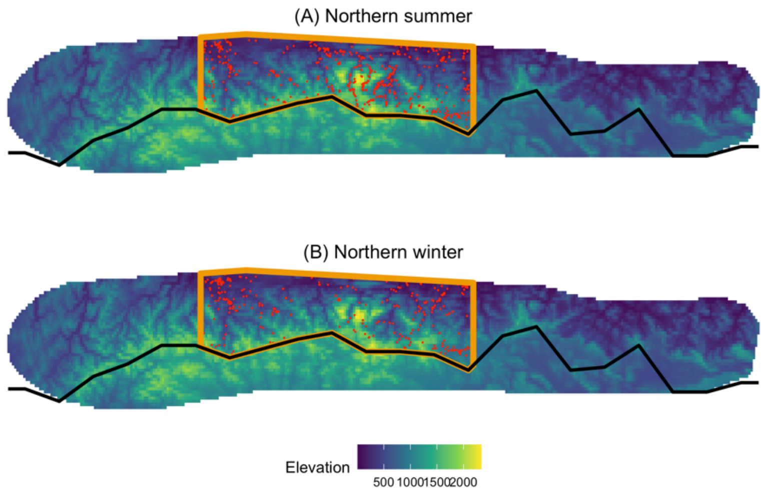

Fig. S15. Location of the *Cord. Cantábrica* mountain slope within the Cantabrian Mountains range. The black line indicates the spatial line splitting the mountain range, and the orange line delineates the mountain slope polygon. Red points indicate the location of eBird checklists during the boreal summer (A) and boreal winter (B).

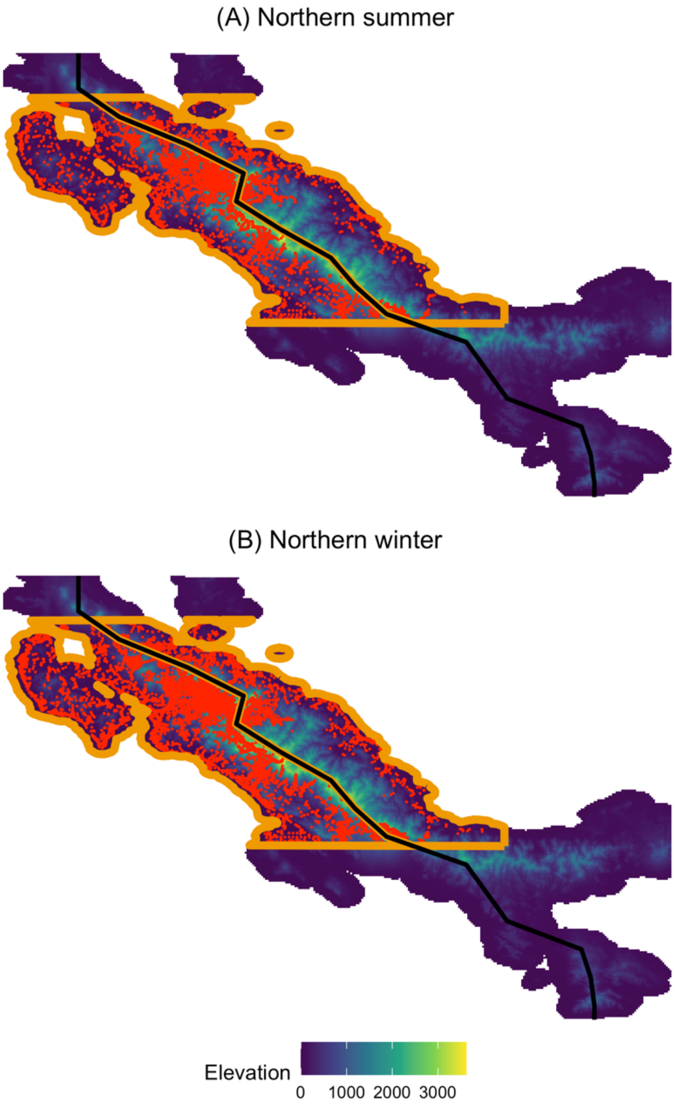

Fig. S16. Location of the *Costa Rica Pacific* and *Costa Rica Caribbean* mountain slopes within the Cordillera Centroamericana mountain range. The black line indicates the spatial line splitting the mountain range, and the orange lines delineate the mountain slope polygons. Red points indicate the location of eBird checklists during the boreal summer (A) and boreal winter (B).

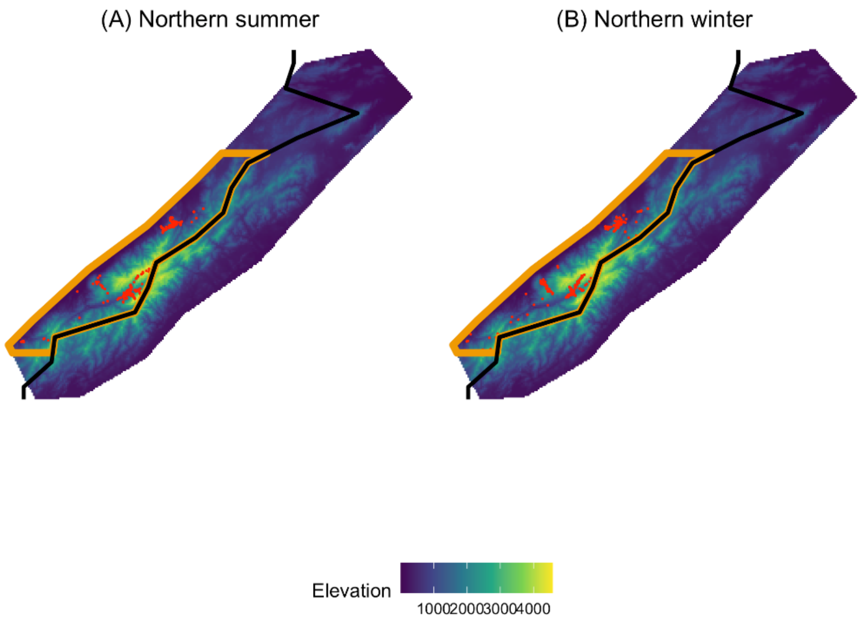

Fig. S17. Location of the *Cord. de Mérida* mountain slope within the Cordillera de Mérida mountain range. The black line indicates the spatial line splitting the mountain range, and the orange line delineates the mountain slope polygon. Red points indicate the location of eBird checklists during the boreal summer (A) and boreal winter (B).

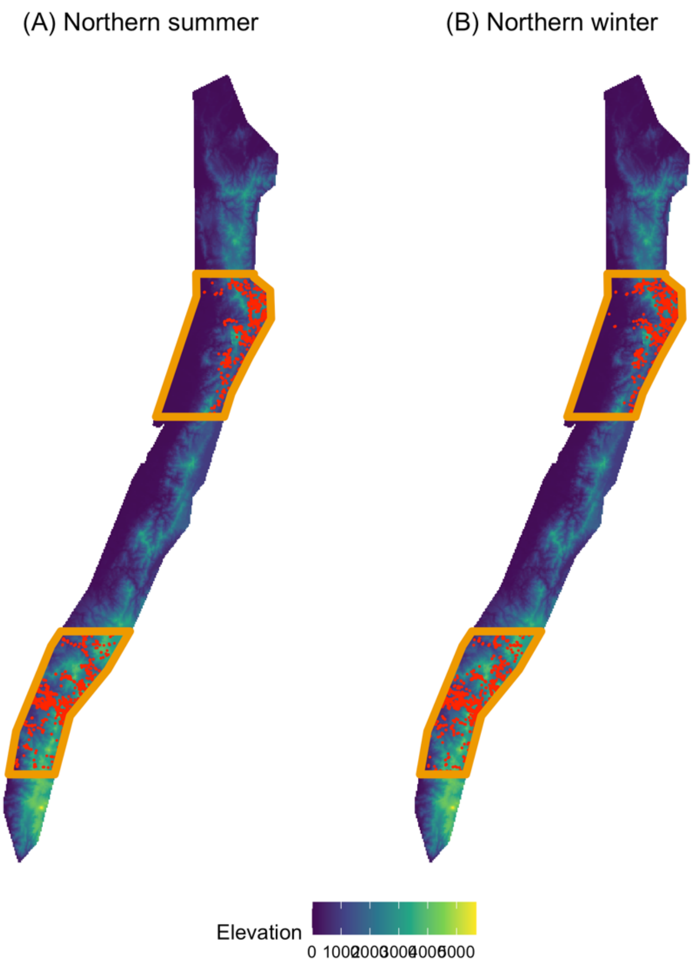

Fig. S18. Location of the *Colombia Pacific* and *Ecuador Pacific* mountain slope within the Cordillera Occidental mountain range in the northern Andes. The orange lines delineate the mountain slope polygons. Red points indicate the location of eBird checklists during the boreal summer (A) and boreal winter (B).

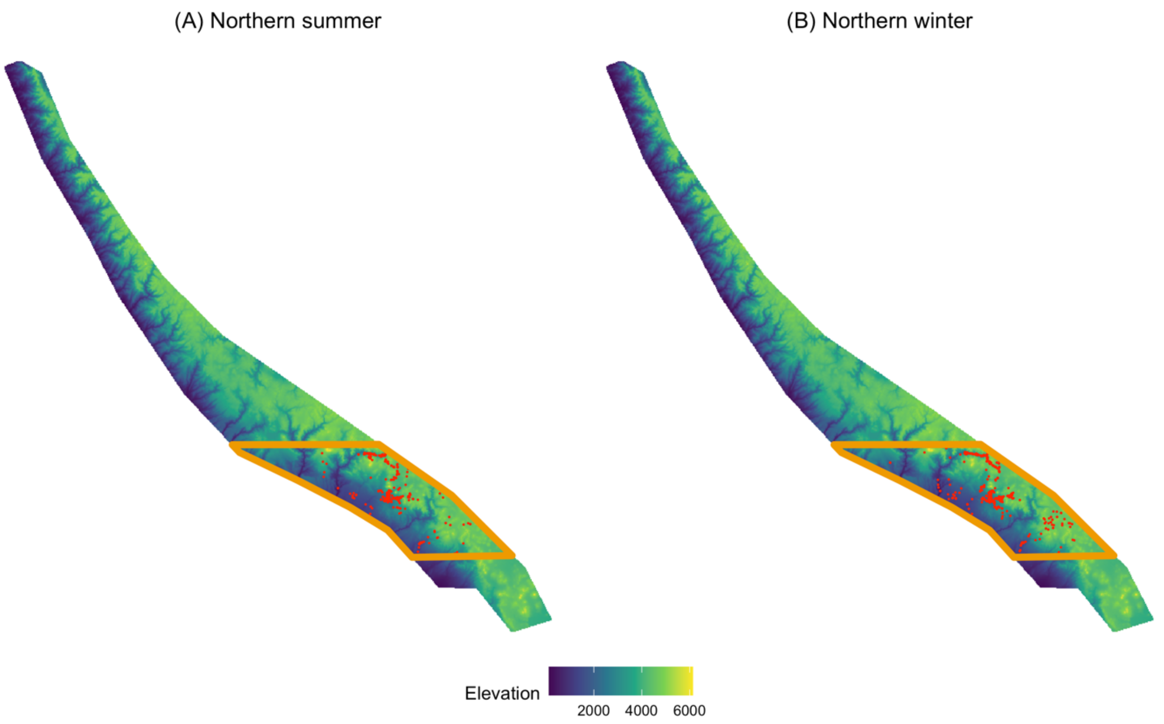

Fig. S19. Location of the *Peru Pacific* mountain slope within the Cordillera Occidental mountain range in the central Andes. The orange line delineates the mountain slope polygon. Red points indicate the location of eBird checklists during the boreal summer (A) and boreal winter (B).

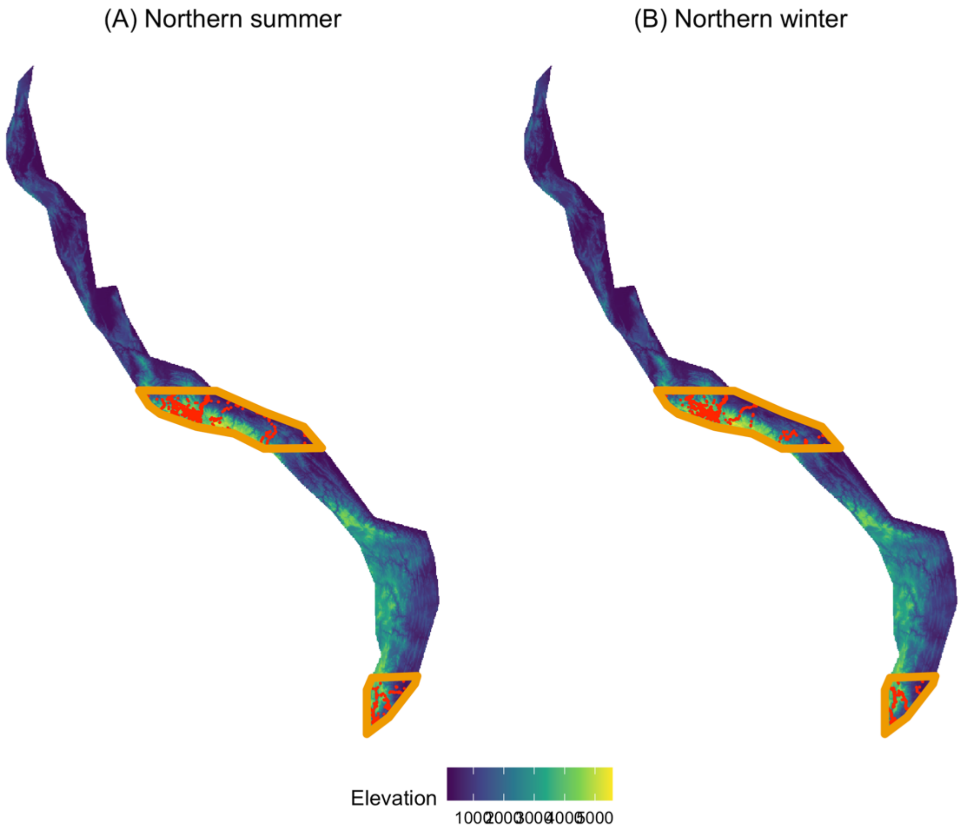

Fig. S20. Location of the *Peru Amazon* and *Argentina North* mountain slopes within the Cordillera Oriental mountain range in the central Andes. The orange lines delineate the mountain slope polygons. Red points indicate the location of eBird checklists during the boreal summer (A) and boreal winter (B).

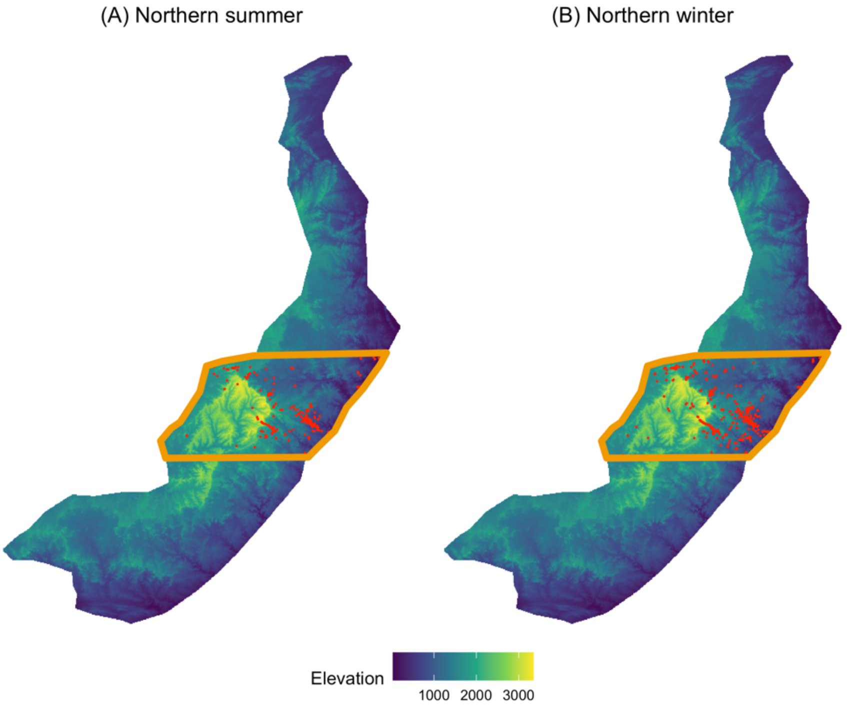

Fig. S21. Location of the *Drakensberg* mountain slope within the Drakensberg escarpment. The orange line delineated the mountain slope polygon. Red points indicate the location of eBird checklists during the boreal summer (A) and boreal winter (B).

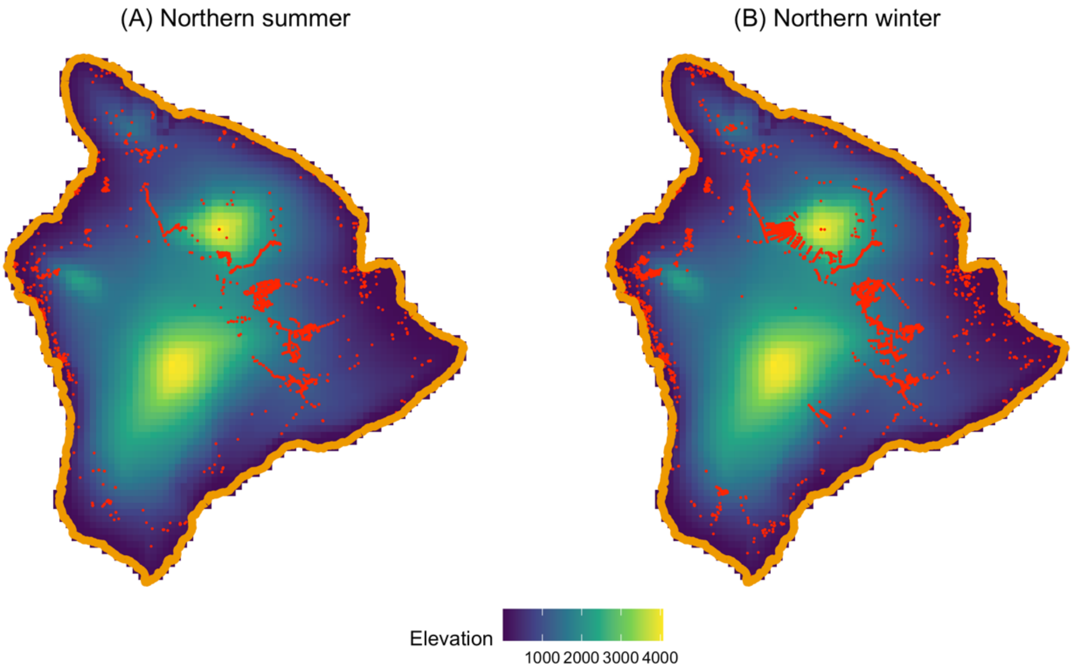

Fig. S22. ***Hawaii (Big Island)*** **mountain slope.** The orange line delineated the mountain slope polygon. Red points indicate the location of eBird checklists during the boreal summer (A) and boreal winter (B).

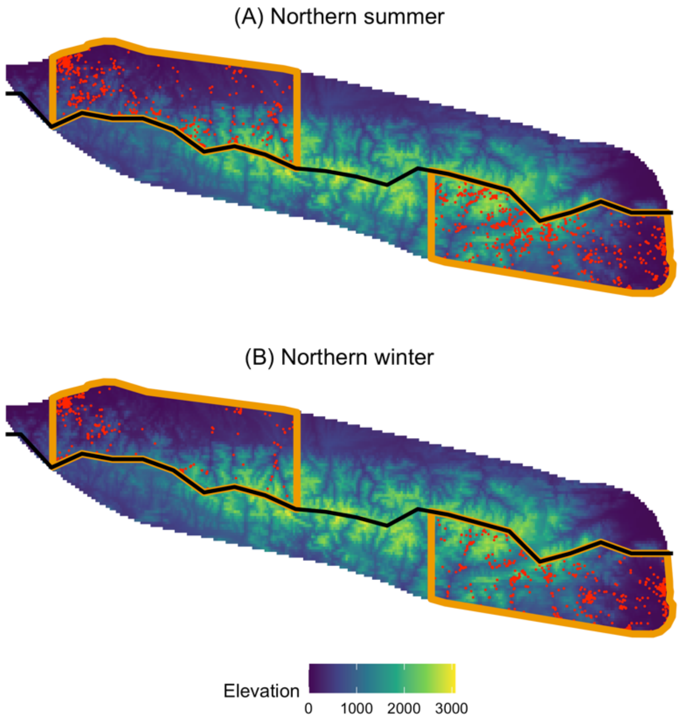

Fig. S23. Location of the *Pyrenees Atlantic* and *Pyrenees Catalonia* mountain slopes within the Pyrenees mountain range. The black line indicates the spatial line splitting the mountain range, and the orange lines delineate the mountain slope polygons. Red points indicate the location of eBird checklists during the boreal summer (A) and boreal winter (B).

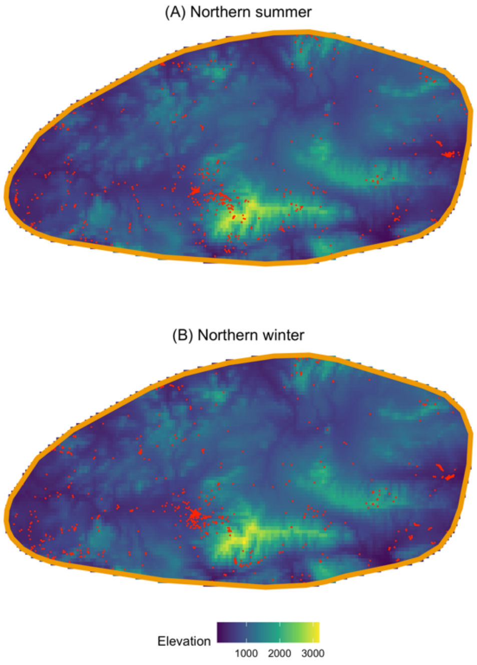

Fig. S24. ***Sierra Nevada Spain*** **mountain slope.** The orange line delineates the mountain slope polygon. Red points indicate the location of eBird checklists during the boreal summer (A) and boreal winter (B).

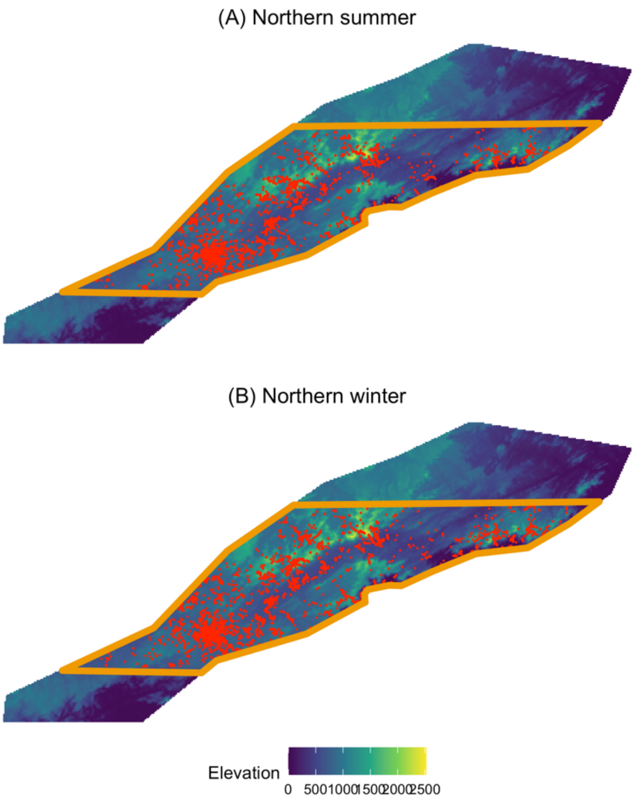

Fig. S25. Location of the ***Serra de Mantiqueira*** **mountain slope within the Serra de Mantiqueira mountain range.** The orange line delineates the mountain slope polygons. Red points indicate the location of eBird checklists during the boreal summer (A) and boreal winter (B).

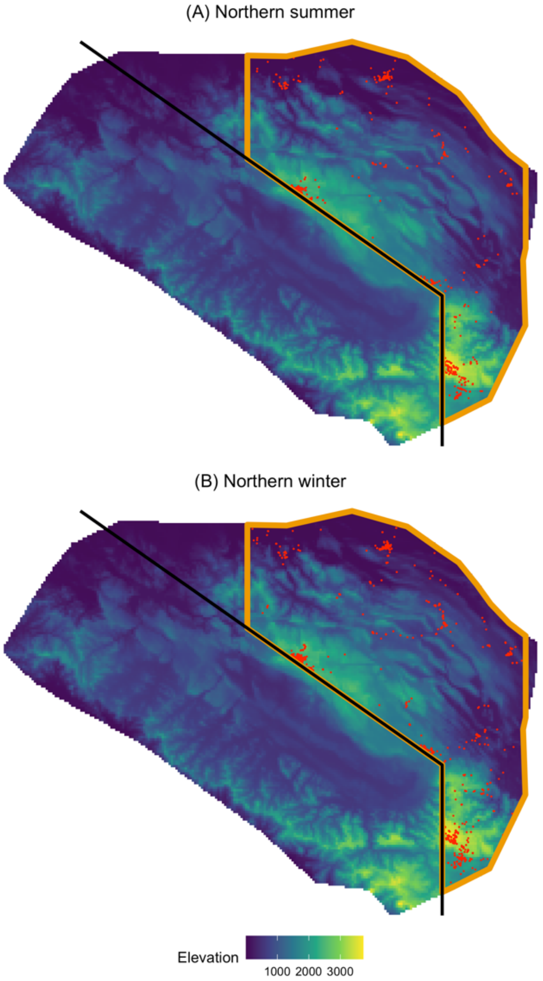

Fig. S26. Location of the *Chiapas* mountain slope within the Sierra Madre de Chiapas mountain range. The black line indicates the spatial line splitting the mountain range, and the orange line delineates the mountain slope polygon. Red points indicate the location of eBird checklists during the boreal summer (A) and boreal winter (B).

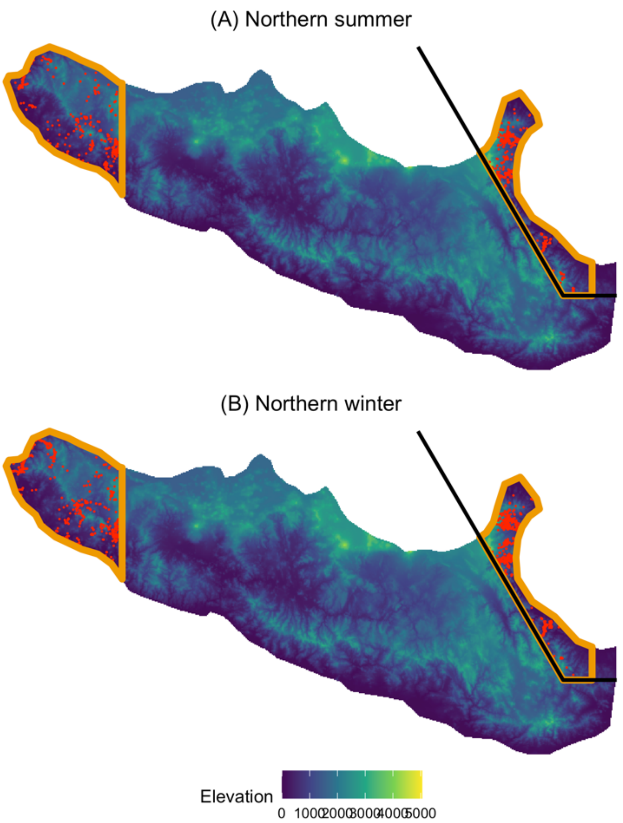

Fig. S27. Location of the *Jalisco* and *Veracruz* mountain slopes within the Sierra Madre del Sur mountain range. The black line indicates the spatial line splitting the mountain range, and the orange lines delineate the mountain slope polygons. Red points indicate the location of eBird checklists during the boreal summer (A) and boreal winter (B).

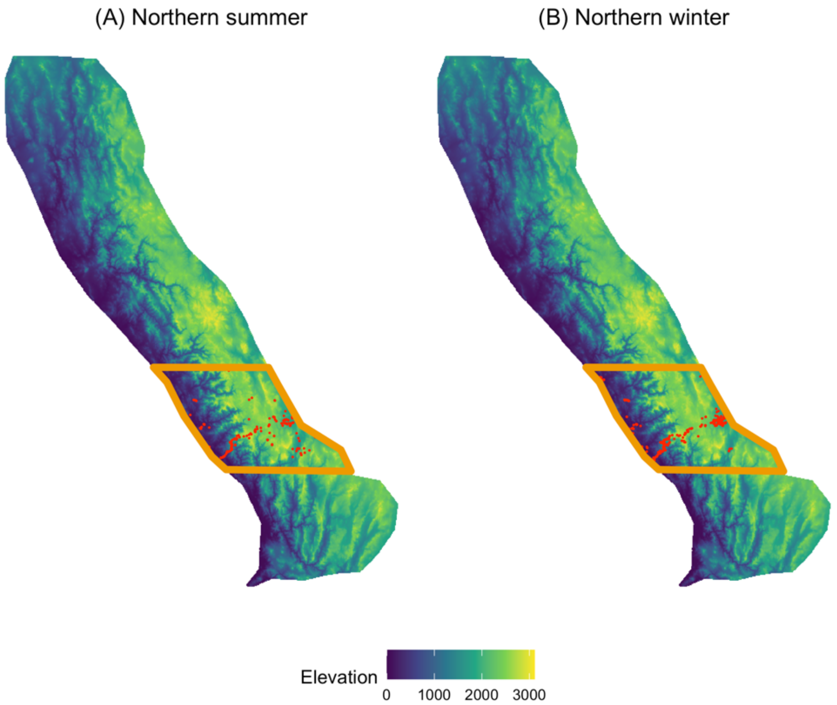

Fig. S28. Location of the *Sierra Occidental* mountain slope within the Sierra Madre Occidental mountain range. The orange line delineates the mountain slope polygon. Red points indicate the location of eBird checklists during the boreal summer (A) and boreal winter (B).

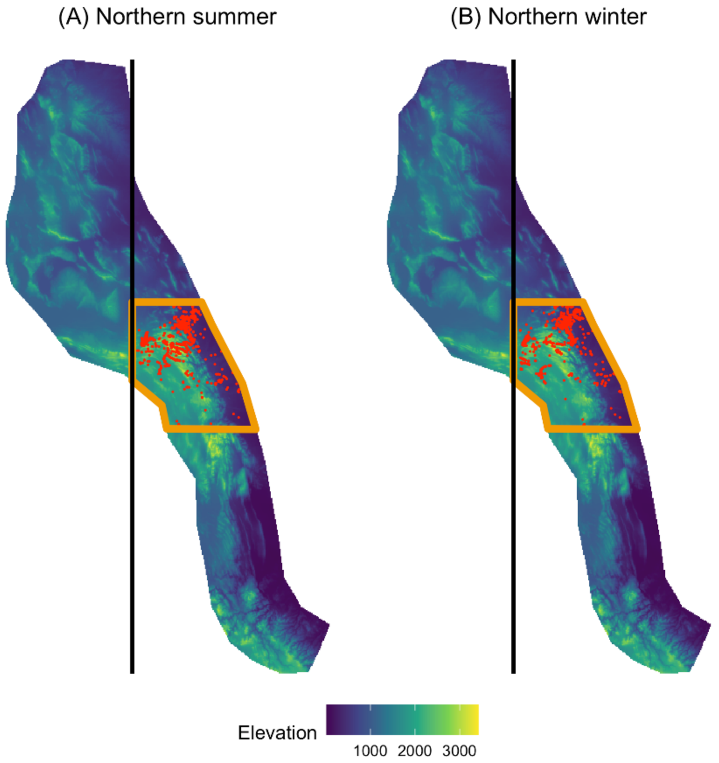

Fig. S29. Location of the *Nuevo Leon* mountain slope within the Sierra Madre Oriental mountain range. The black line indicates the spatial line splitting the mountain range, and the orange line delineates the mountain slope polygon. Red points indicate the location of eBird checklists during the boreal summer (A) and boreal winter (B).

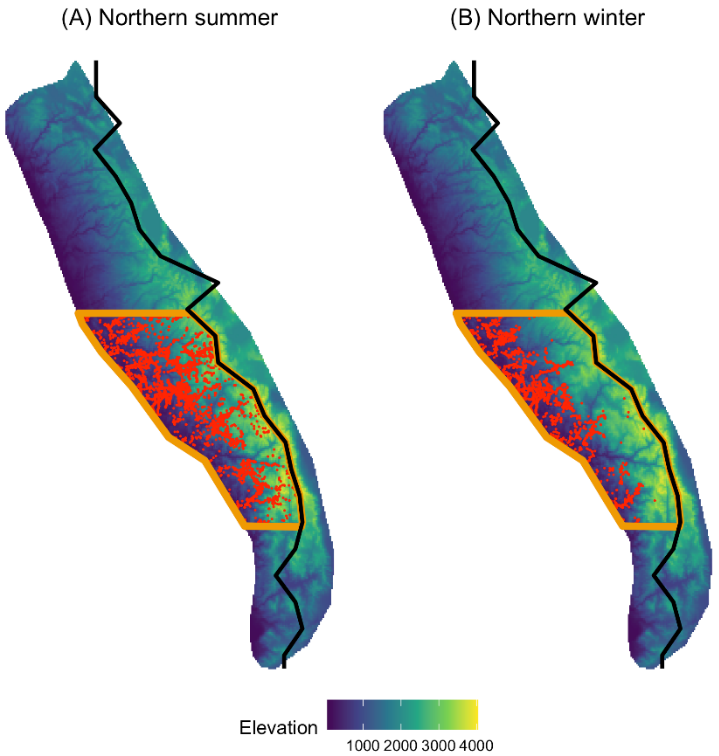

Fig. S30. Location of the *Sierra Nevada Cal* mountain slope within the Sierra Nevada mountain range in the western USA. The black line indicates the spatial line splitting the mountain range, and the orange line delineates the mountain slope polygon. Red points indicate the location of eBird checklists during the boreal summer (A) and boreal winter (B).

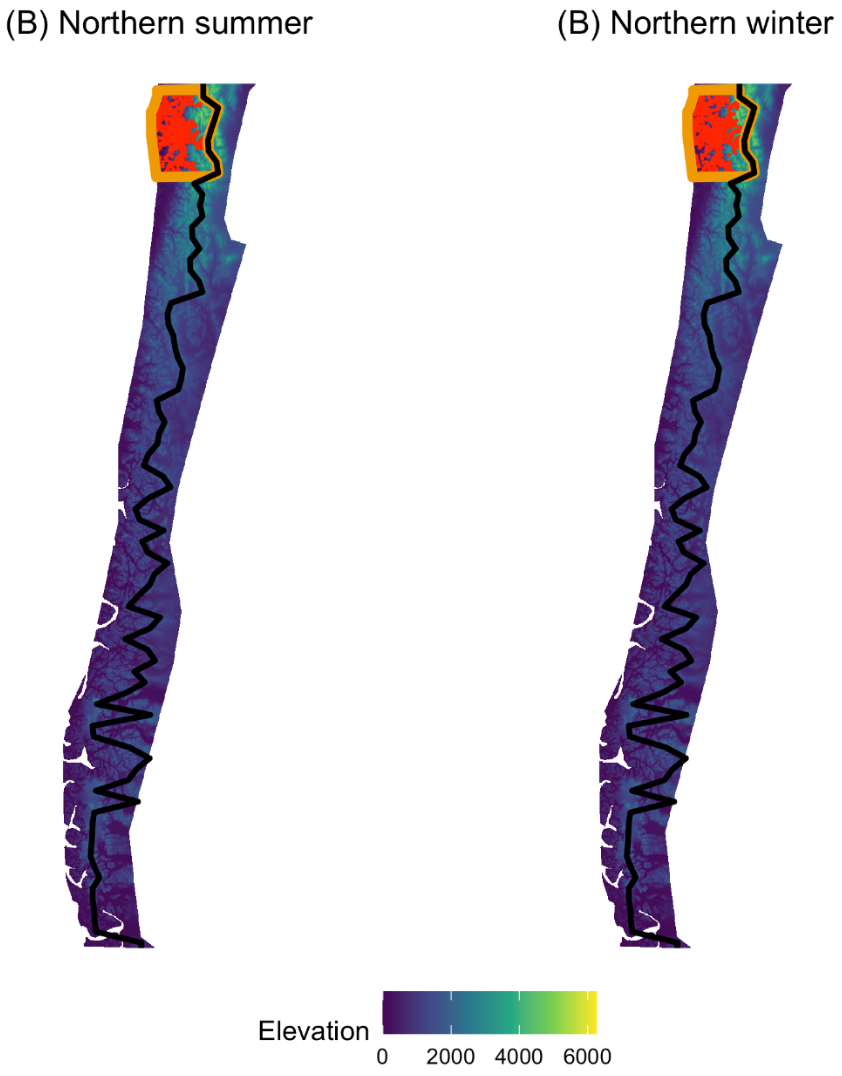

Fig. S31. Location of the *Chile Central* mountain slope within the Southern Andes mountain range. The black line indicates the spatial line splitting the mountain range, and the orange line delineates the mountain slope polygon. Red points indicate the location of eBird checklists during the boreal summer (A) and boreal winter (B).

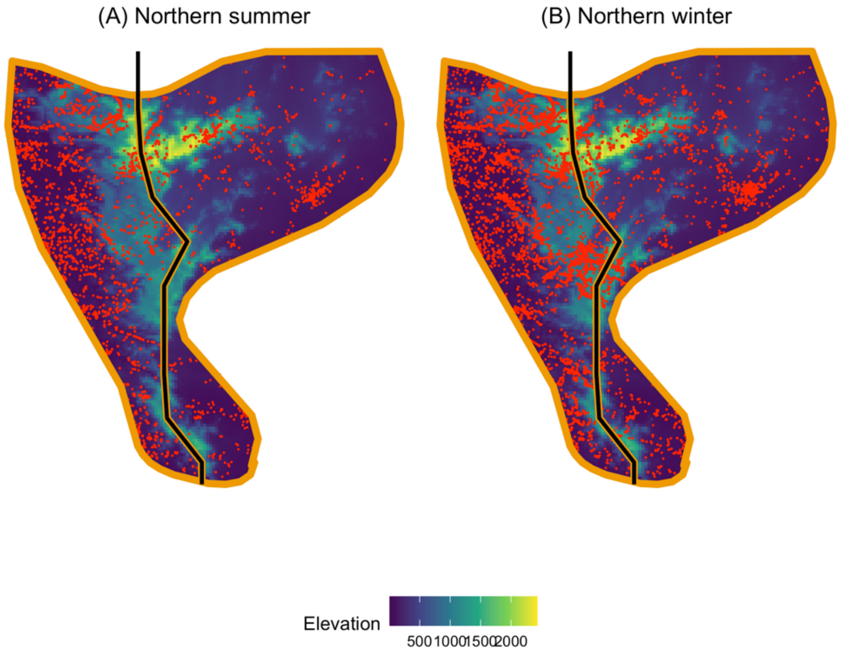

Fig. S32. Location of the *Southern Ghats West* and *Southern Ghats East* mountain slopes within the Southern Ghats mountain range. The black line indicates the spatial line splitting the mountain range, and the orange lines delineate the mountain slope polygons. Red points indicate the location of eBird checklists during the boreal summer (A) and boreal winter (B).

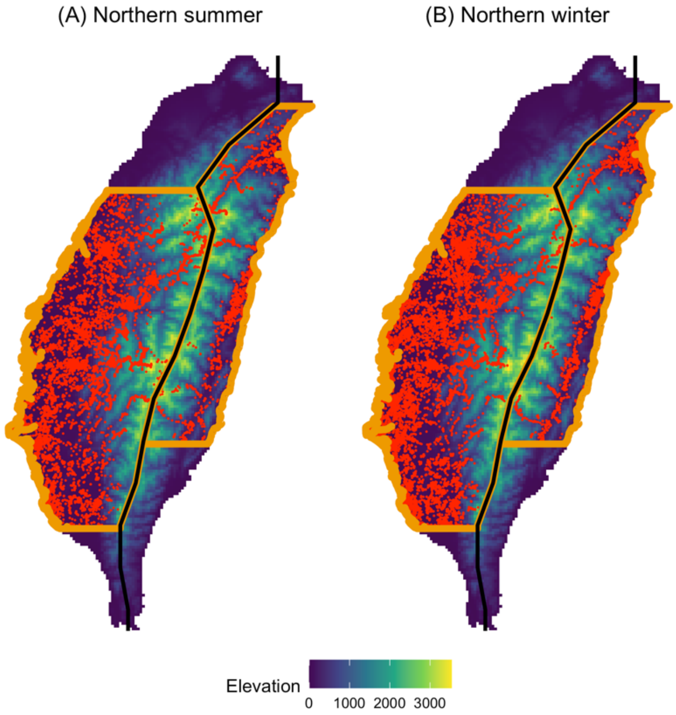

Fig. S33. Location of the *Taiwan West* and *Taiwan East* mountain slopes within the main island of Taiwan. The black line indicates the spatial line splitting the mountain range, and the orange lines delineate the mountain slope polygons. Red points indicate the location of eBird checklists during the boreal summer (A) and boreal winter (B).

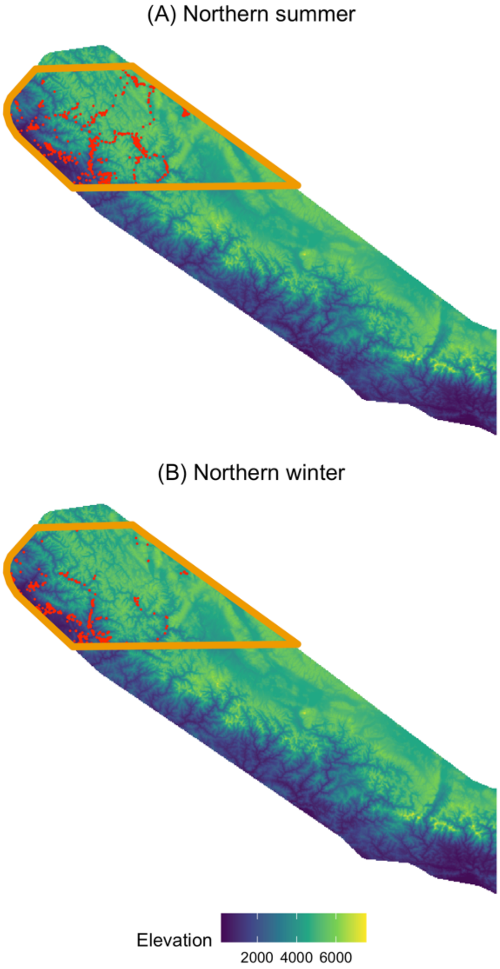

Fig. S34. Location of the *Western Himalayas* mountain slopes within the Western Himalayas mountain range. The orange line delineates the mountain slope polygon. Red points indicate the location of eBird checklists during the boreal summer (A) and boreal winter (B).

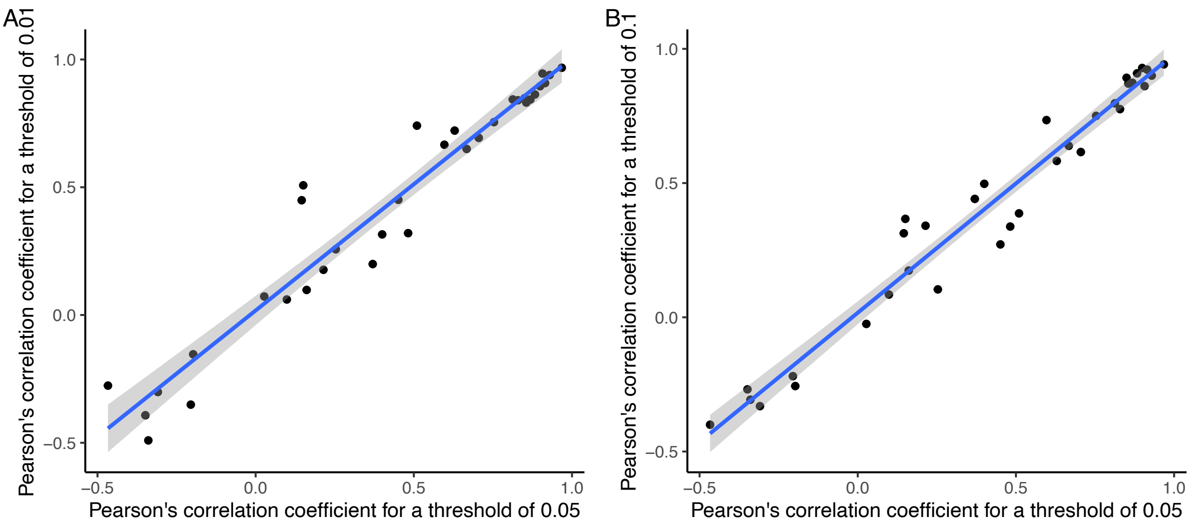

Fig. S35. Results of the sensitivity analysis for the threshold for delineating range limits. The Pearson's correlation coefficients between model predictions using a threshold for delineating range limits of 0.05 and observation for the seasonal difference in species richness across all the mountain slopes in our dataset, were highly correlated with the Pearson's correlation coefficients obtained with a threshold for delineating range limits of 0.01 (A) and 0.1 (B).

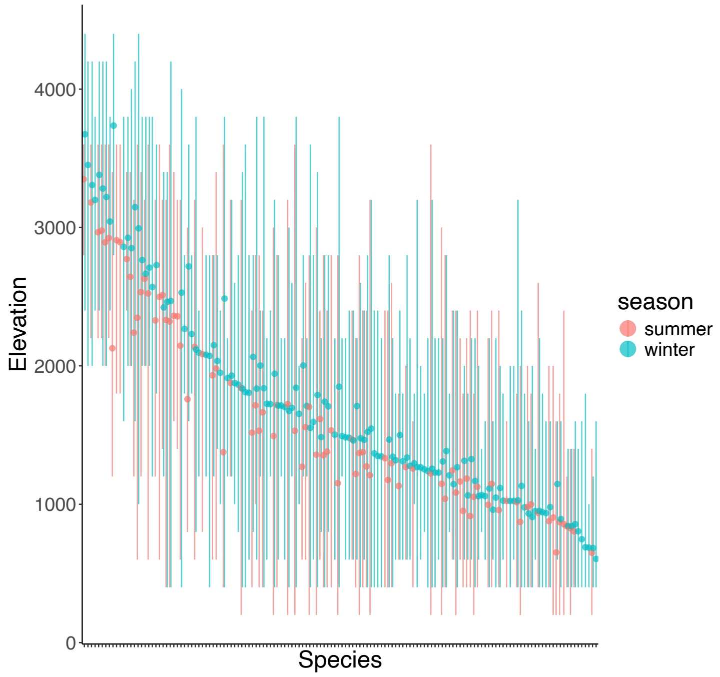

Fig. S36. Seasonal elevational ranges of avian species in *Argentina North*. Each vertical line indicates the elevational range of a given species, during the boreal summer (in red) or boreal winter (in blue). Points indicates seasonal mean elevations.

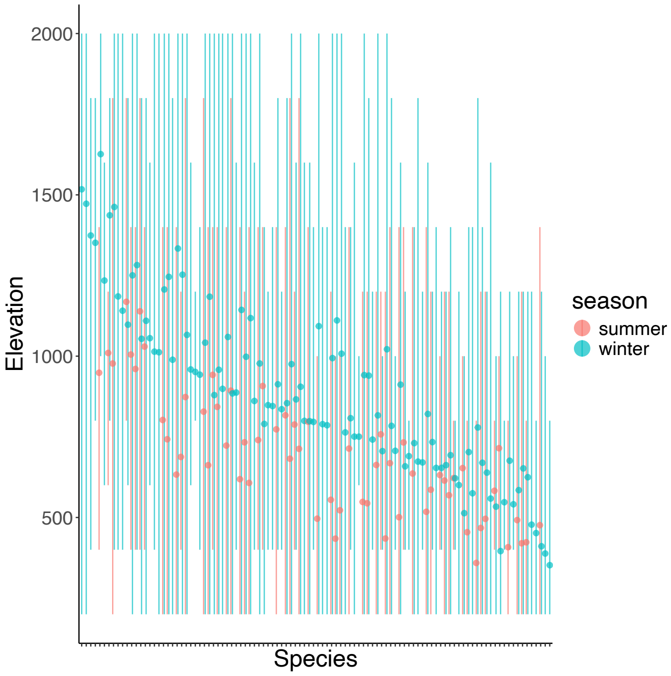

Fig. S37. Seasonal elevational ranges of avian species in *Australian Alps*. Each vertical line indicates the elevational range of a given species, during the boreal summer (in red) or boreal winter (in blue). Points indicates seasonal mean elevations.

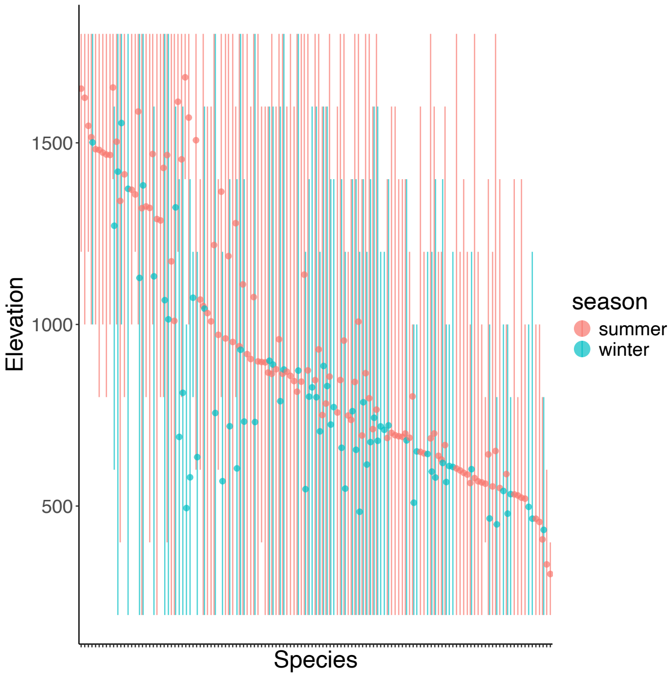

Fig. S38. Seasonal elevational ranges of avian species in *Blue Ridge*. Each vertical line indicates the elevational range of a given species, during the boreal summer (in red) or boreal winter (in blue). Points indicates seasonal mean elevations.

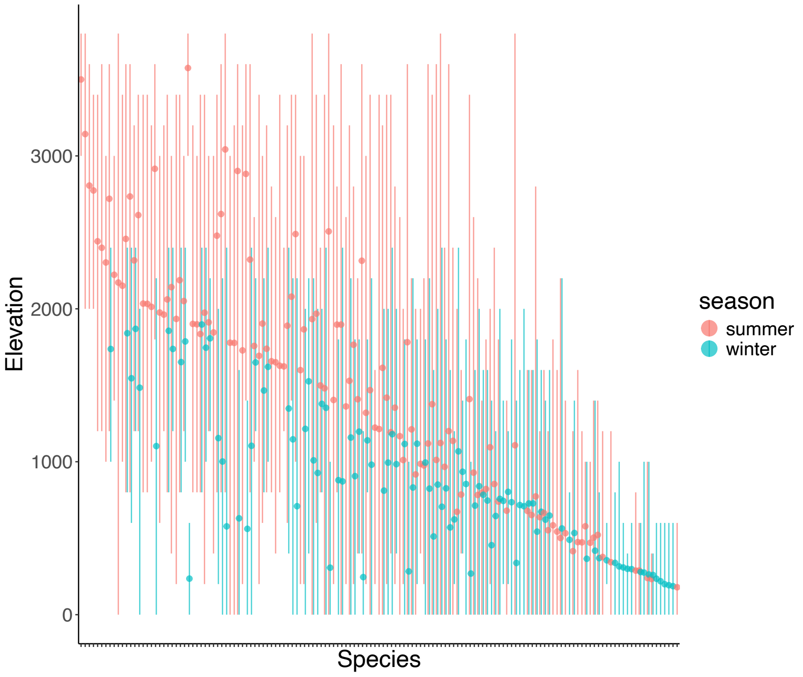

Fig. S39. Seasonal elevational ranges of avian species in *Sierra Nevada Cal*. Each vertical line indicates the elevational range of a given species, during the boreal summer (in red) or boreal winter (in blue). Points indicates seasonal mean elevations.

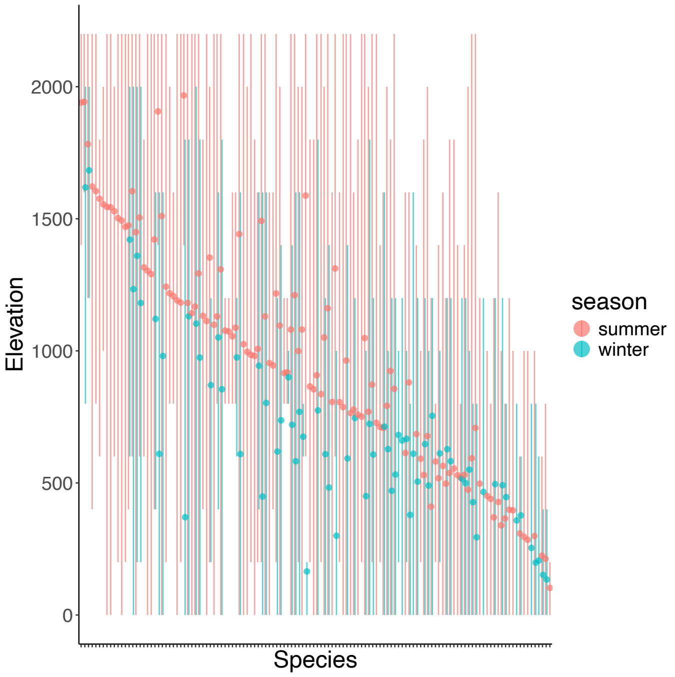

Fig. S40. Seasonal elevational ranges of avian species in *Cascade East*. Each vertical line indicates the elevational range of a given species, during the boreal summer (in red) or boreal winter (in blue). Points indicates seasonal mean elevations.

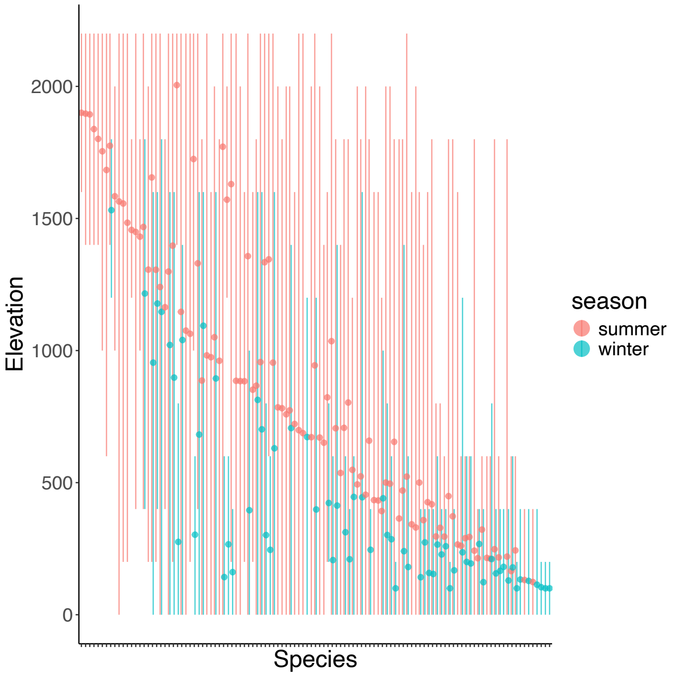

Fig. S41. Seasonal elevational ranges of avian species in *Cascade West*. Each vertical line indicates the elevational range of a given species, during the boreal summer (in red) or boreal winter (in blue). Points indicates seasonal mean elevations.

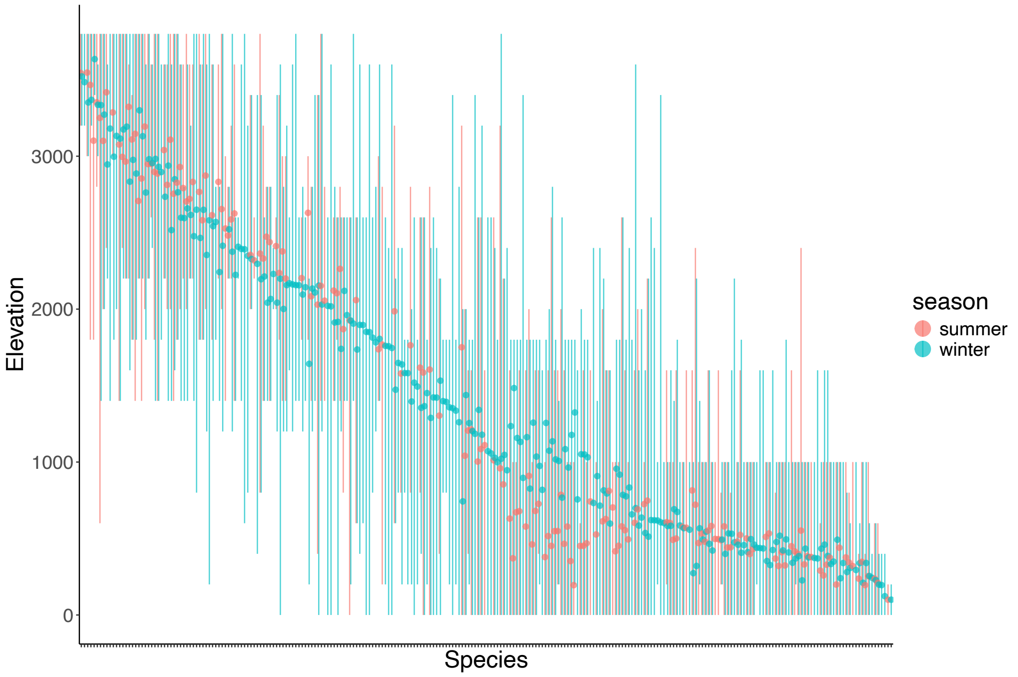

Fig. S42. Seasonal elevational ranges of avian species in *Chiapas East*. Each vertical line indicates the elevational range of a given species, during the boreal summer (in red) or boreal winter (in blue). Points indicates seasonal mean elevations.

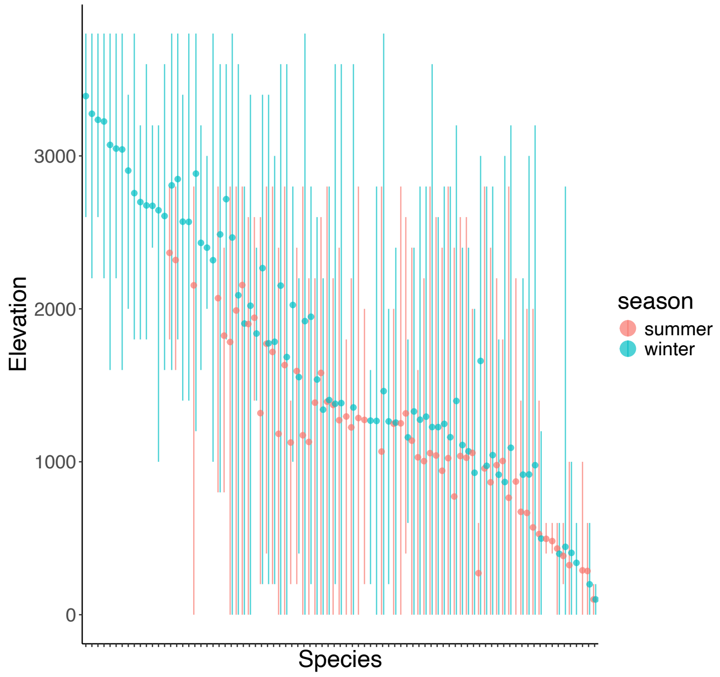

Fig. S43. Seasonal elevational ranges of avian species in *Chile Central*. Each vertical line indicates the elevational range of a given species, during the boreal summer (in red) or boreal winter (in blue). Points indicates seasonal mean elevations.

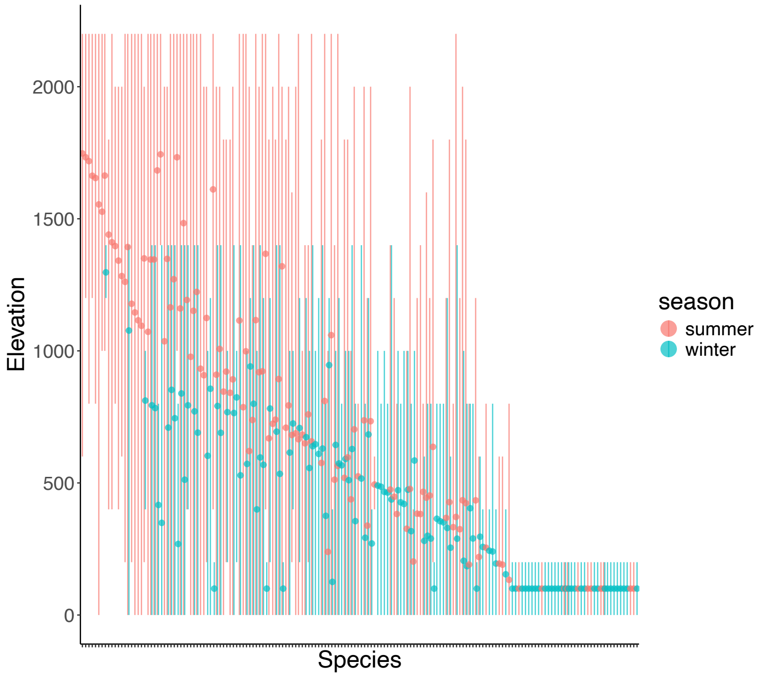

Fig. S44. Seasonal elevational ranges of avian species in *Coastal Range*. Each vertical line indicates the elevational range of a given species, during the boreal summer (in red) or boreal winter (in blue). Points indicates seasonal mean elevations.

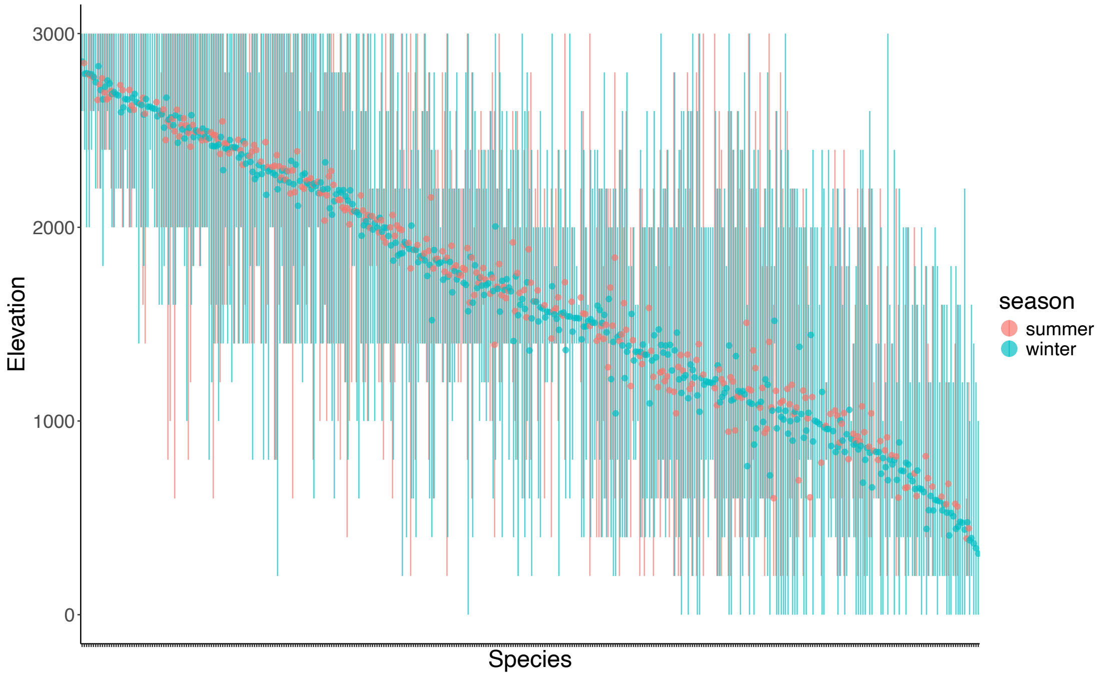

Fig. S45. Seasonal elevational ranges of avian species in *Colombia Pacific*. Each vertical line indicates the elevational range of a given species, during the boreal summer (in red) or boreal winter (in blue). Points indicates seasonal mean elevations.

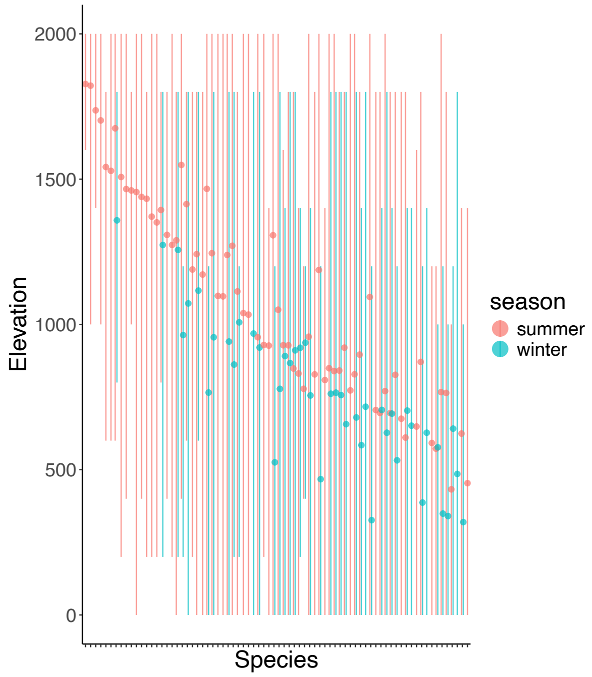

Fig. S46. Seasonal elevational ranges of avian species in *Cordillera Cantabrica*. Each vertical line indicates the elevational range of a species, during the boreal summer (red) or winter (blue). Points indicates seasonal mean elevations.

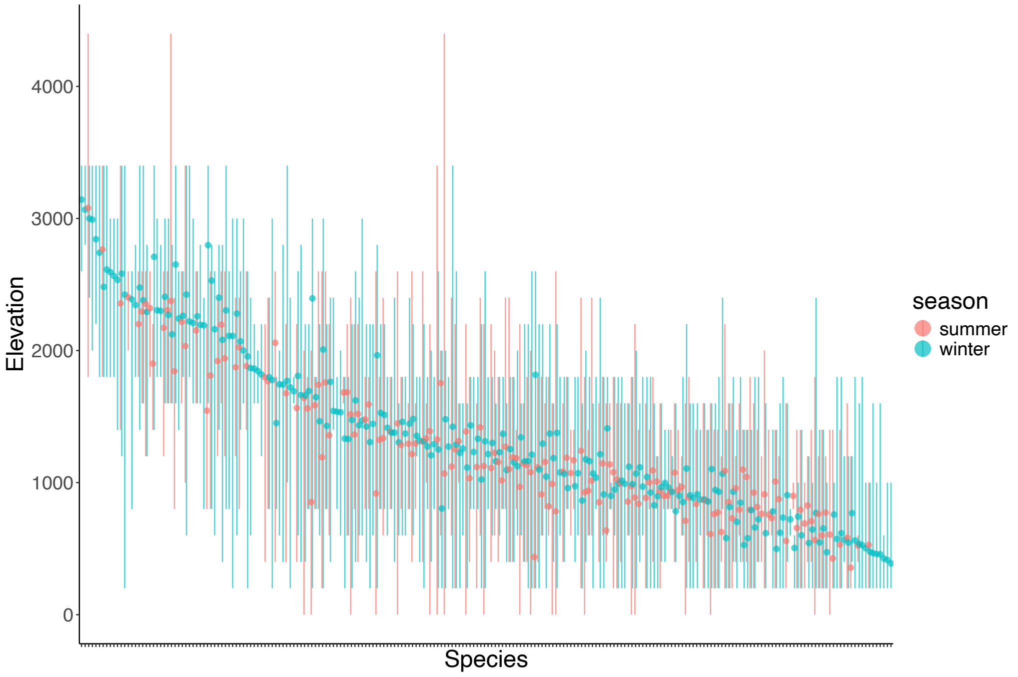

Fig. S47. Seasonal elevational ranges of avian species in *Cordillera de Merida*. Each vertical line indicates the elevational range of a species, during the boreal summer (red) or winter (blue). Points indicates seasonal mean elevations.

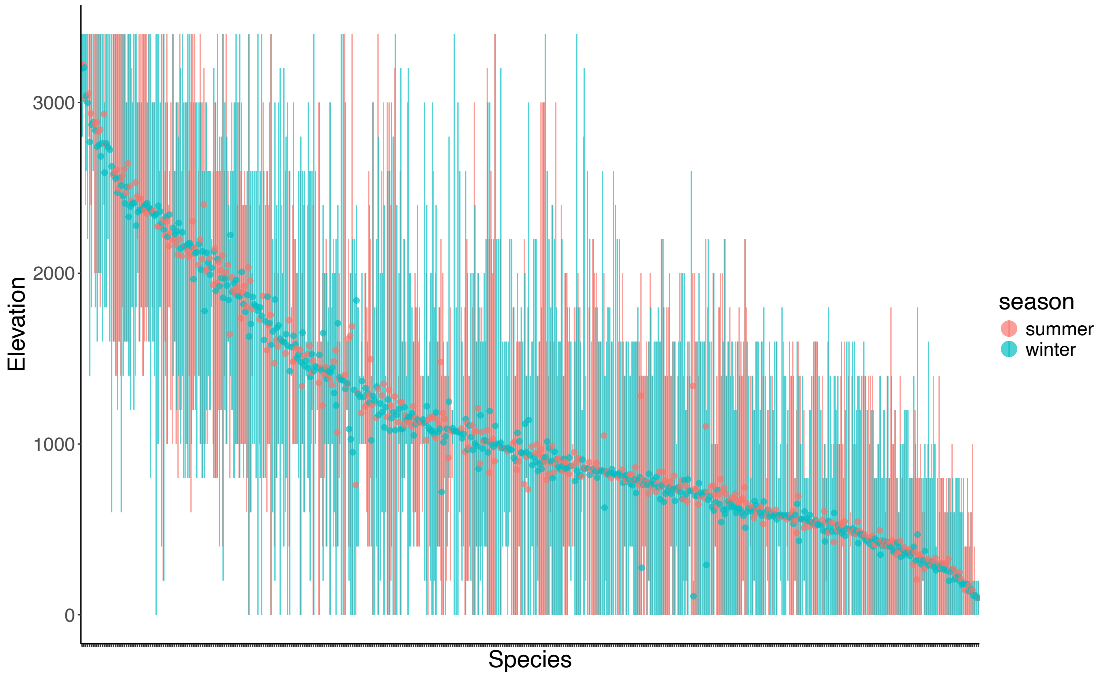

Fig. S48. Seasonal elevational ranges of avian species in *Costa Rica Caribbean*. Each vertical line indicates the elevational range of a species, during the boreal summer (red) or winter (blue). Points indicates seasonal mean elevations.

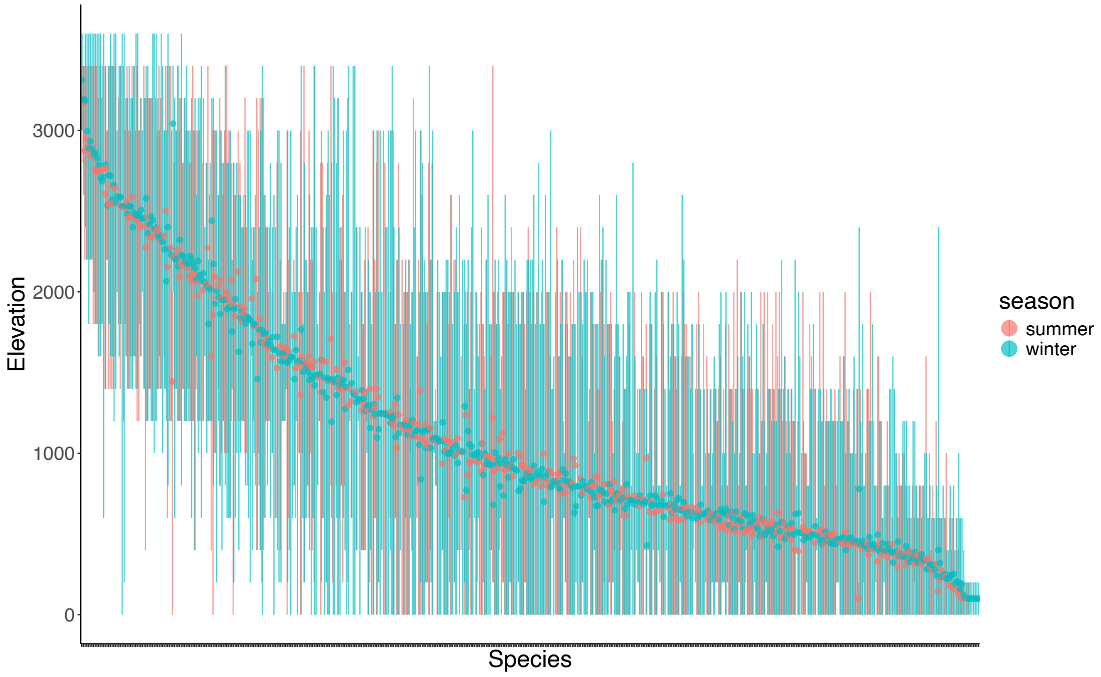

Fig. S49. Seasonal elevational ranges of avian species in *Costa Rica Pacific*. Each vertical line indicates the elevational range of a species, during the boreal summer (red) or winter (blue). Points indicates seasonal mean elevations.

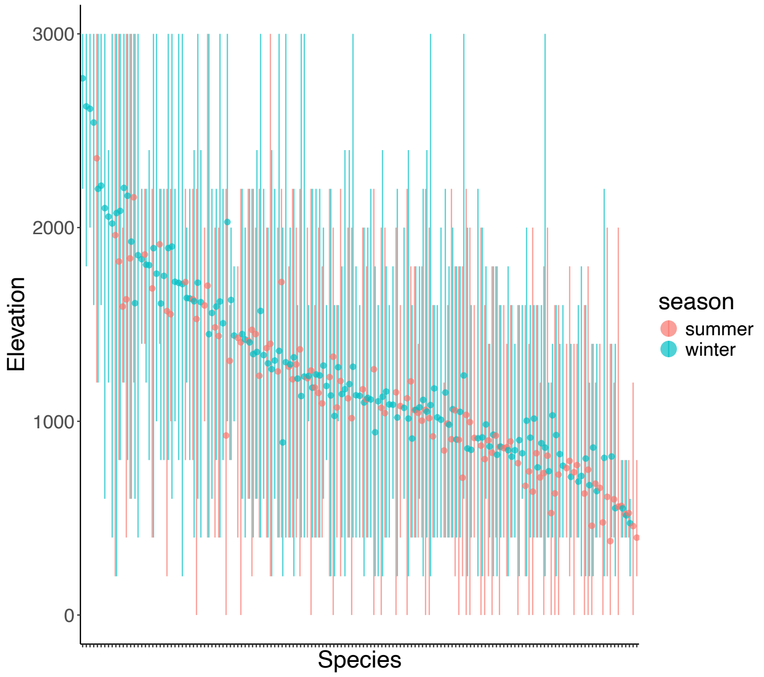

Fig. S50. Seasonal elevational ranges of avian species in *Drakensberg*. Each vertical line indicates the elevational range of a species, during the boreal summer (red) or winter (blue). Points indicates seasonal mean elevations.

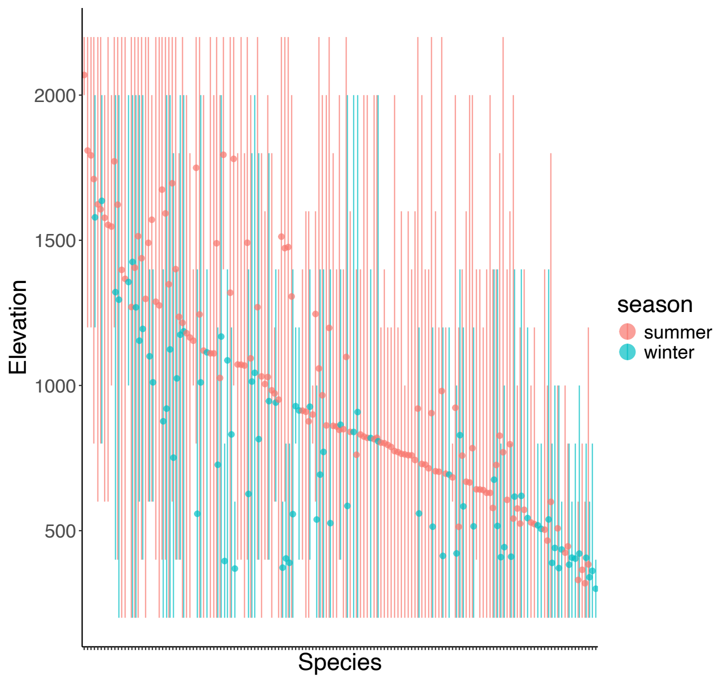

Fig. S51. Seasonal elevational ranges of avian species in *Rocky Mountains N*. Each vertical line indicates the elevational range of a species, during the boreal summer (red) or winter (blue). Points indicates seasonal mean elevations.

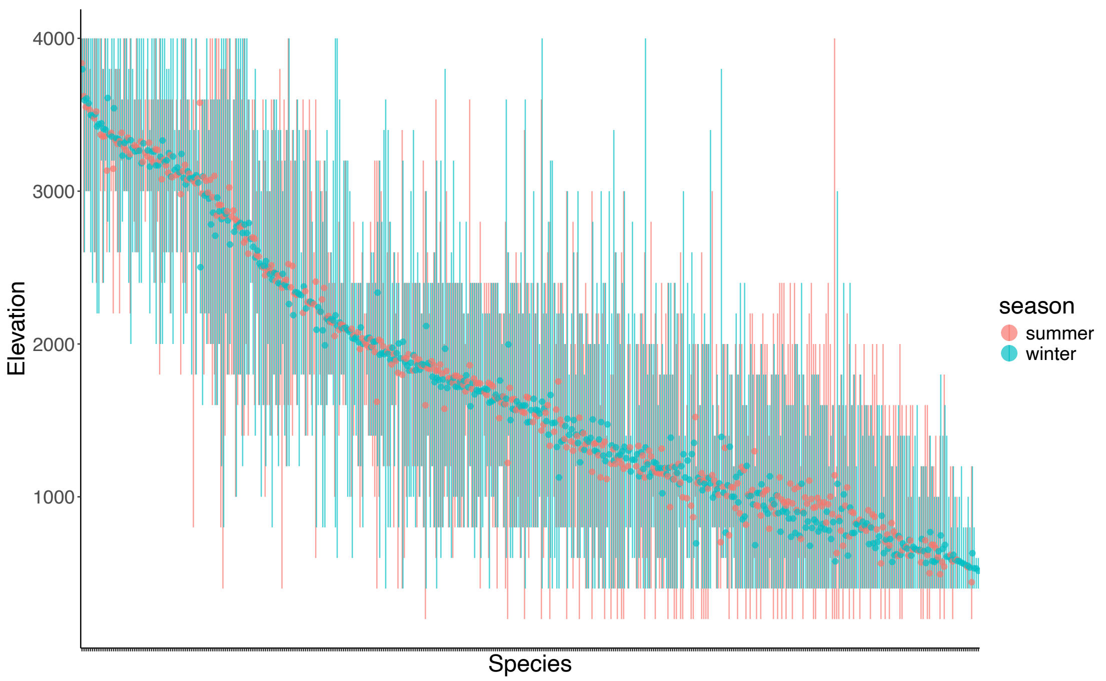

Fig. S52. Seasonal elevational ranges of avian species in *Ecuador Pacific*. Each vertical line indicates the elevational range of a species, during the boreal summer (red) or winter (blue). Points indicates seasonal mean elevations.

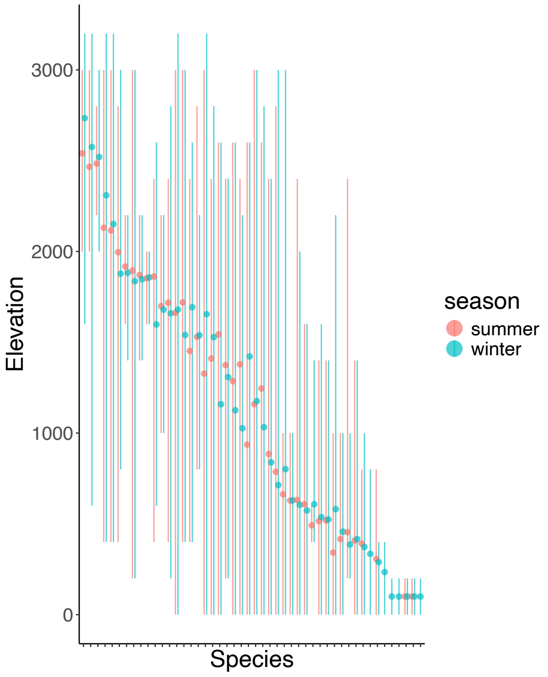

Fig. S53. Seasonal elevational ranges of avian species in *Hawaii (Big Island)*. Each vertical line indicates the elevational range of a species, during the boreal summer (red) or winter (blue). Points indicates seasonal mean elevations.

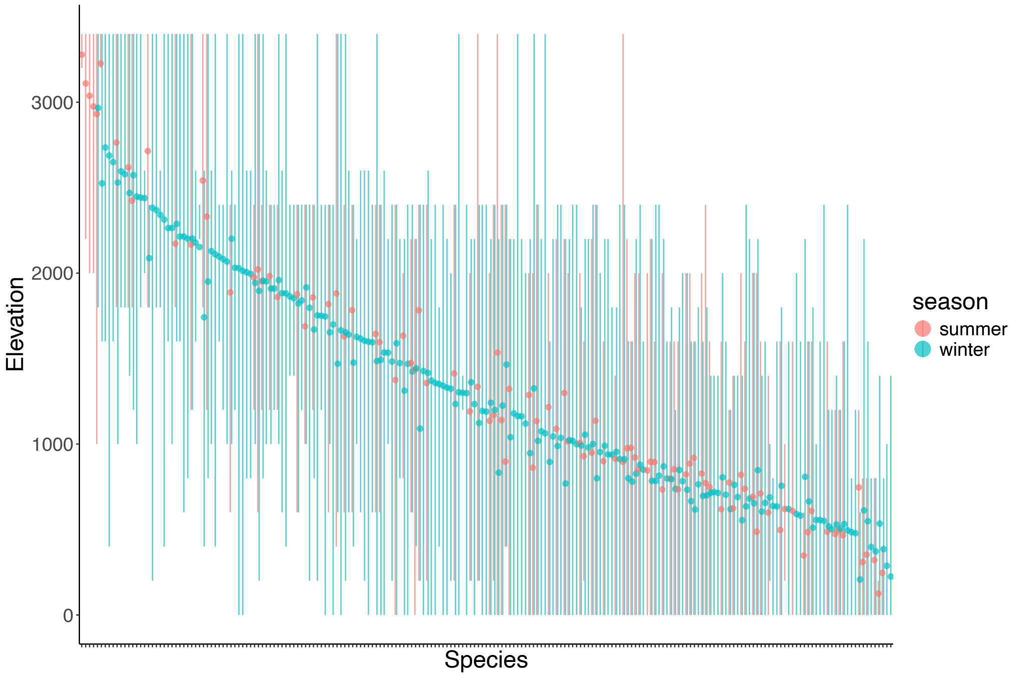

Fig. S54. Seasonal elevational ranges of avian species in *Jalisco*. Each vertical line indicates the elevational range of a species, during the boreal summer (red) or winter (blue). Points indicates seasonal mean elevations.

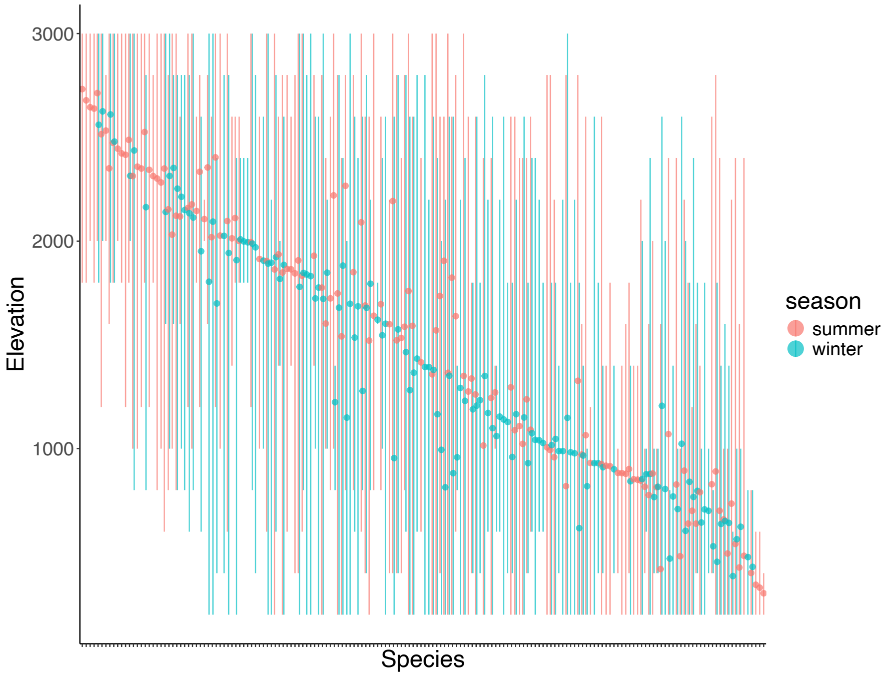

Fig. S55. Seasonal elevational ranges of avian species in *Nuevo Leon*. Each vertical line indicates the elevational range of a species, during the boreal summer (red) or winter (blue). Points indicates seasonal mean elevations.

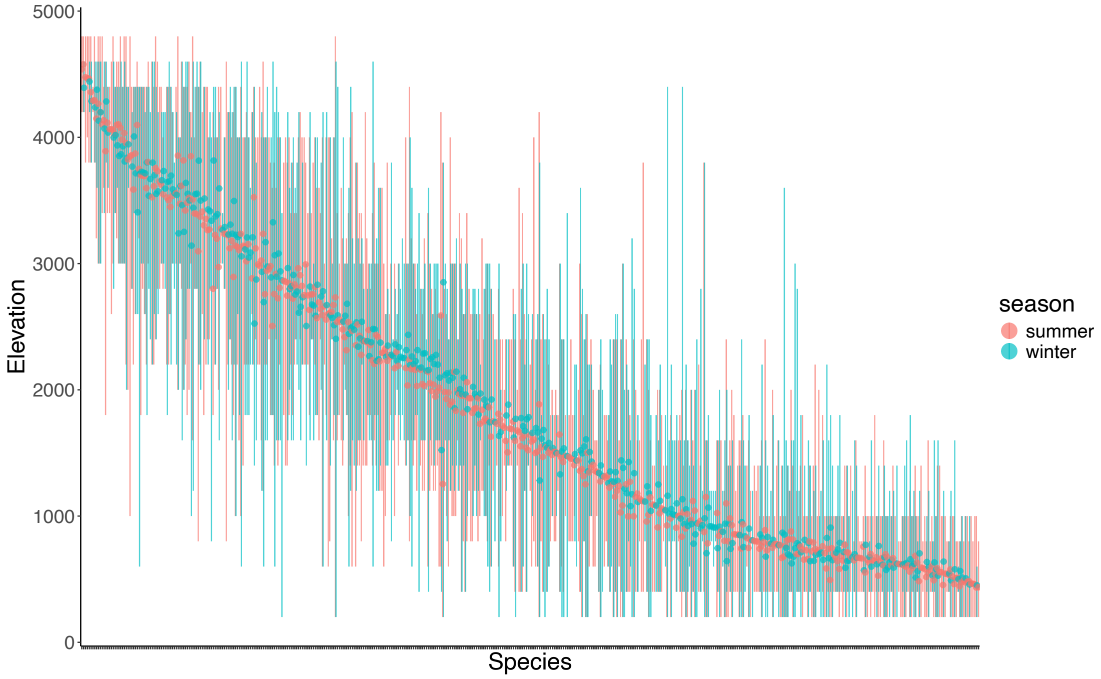

Fig. S56. Seasonal elevational ranges of avian species in *Peru Amazon*. Each vertical line indicates the elevational range of a species, during the boreal summer (red) or winter (blue). Points indicates seasonal mean elevations.

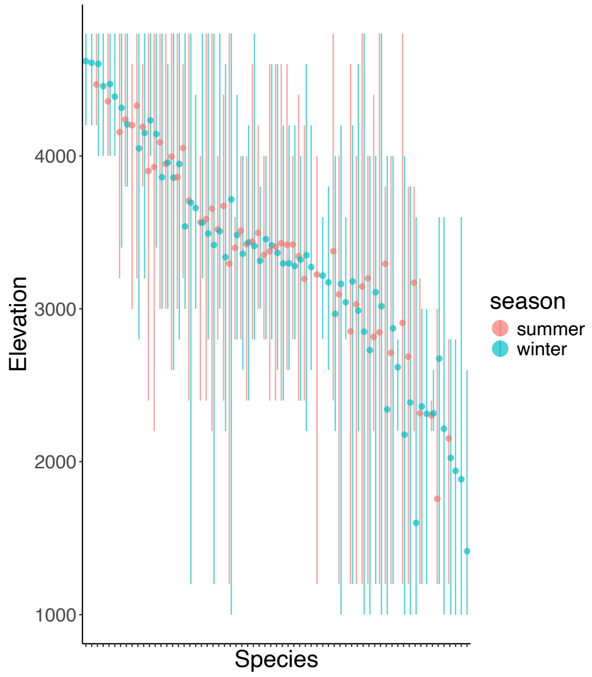

Fig. S57. Seasonal elevational ranges of avian species in *Peru Pacific*. Each vertical line indicates the elevational range of a species, during the boreal summer (red) or winter (blue). Points indicates seasonal mean elevations.

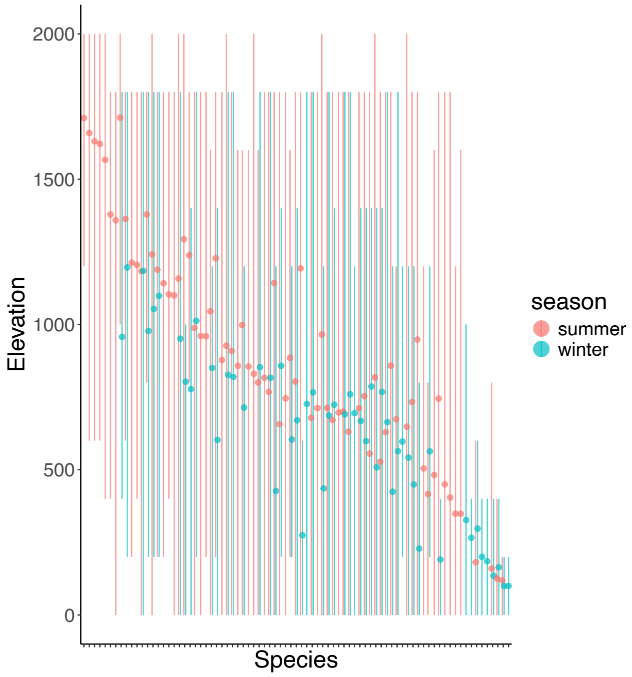

Fig. S58. Seasonal elevational ranges of avian species in *Pyrenees Atlantic*. Each vertical line indicates the elevational range of a species, during the boreal summer (red) or winter (blue). Points indicates seasonal mean elevations.

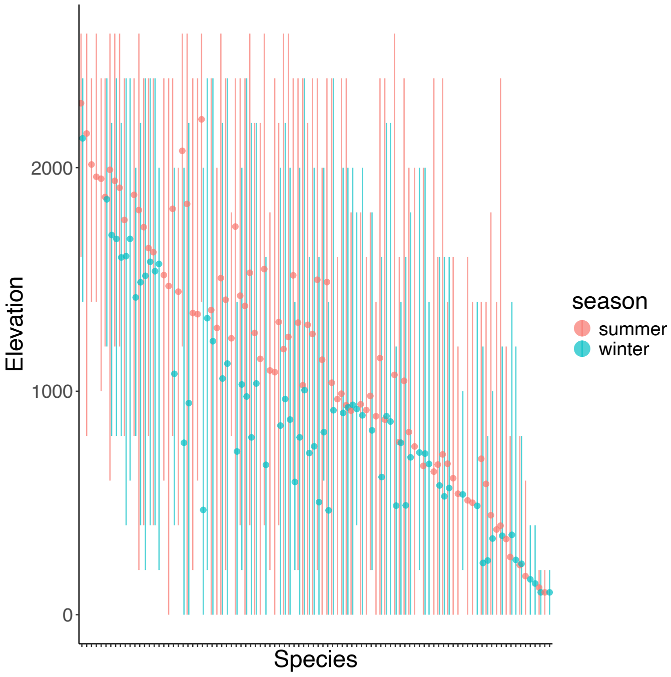

Fig. S59. Seasonal elevational ranges of avian species in *Pyrenees Catalonia*. Each vertical line indicates the elevational range of a species, during the boreal summer (red) or winter (blue). Points indicates seasonal mean elevations.

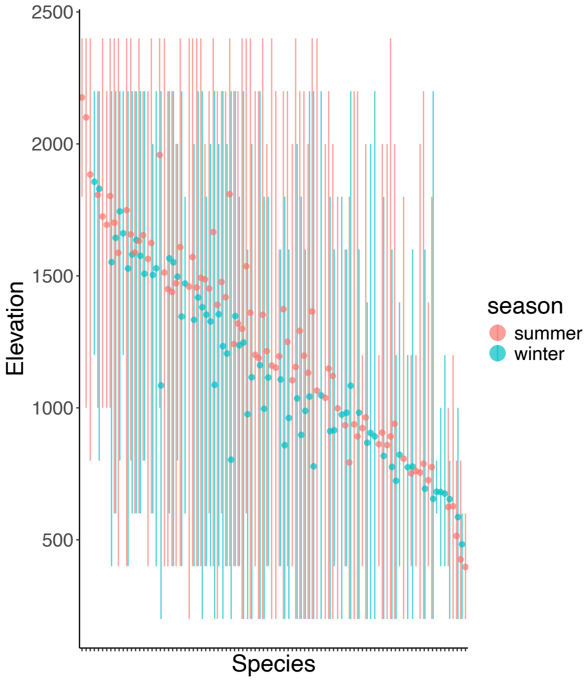

Fig. S60. Seasonal elevational ranges of avian species in *Sierra Nevada Spain*. Each vertical line indicates the elevational range of a species, during the boreal summer (red) or winter (blue). Points indicates seasonal mean elevations.

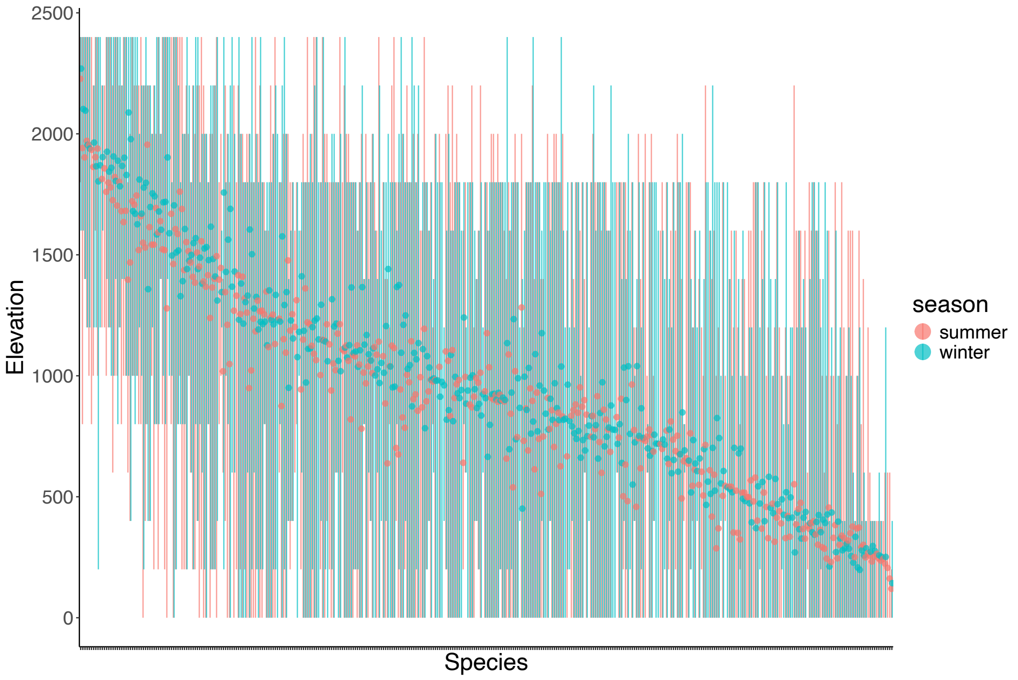

Fig. S61. Seasonal elevational ranges of avian species in *Serra de Mantiqueira*. Each vertical line indicates the elevational range of a species, during the boreal summer (red) or winter (blue). Points indicates seasonal mean elevations.

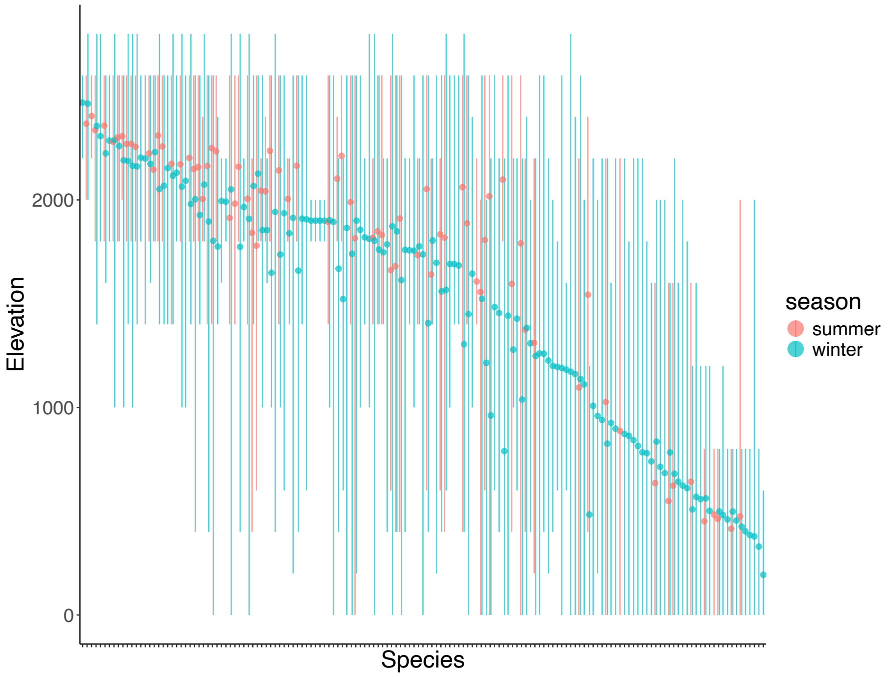

Fig. S62. Seasonal elevational ranges of avian species in *Sierra Occidental*. Each vertical line indicates the elevational range of a species, during the boreal summer (red) or winter (blue). Points indicates seasonal mean elevations.

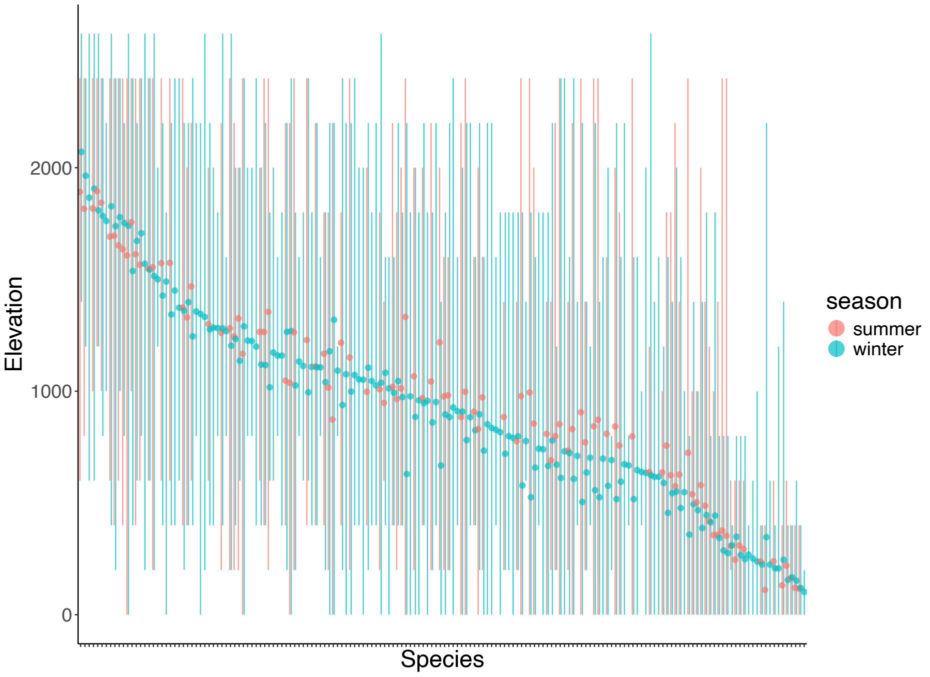

Fig. S63. Seasonal elevational ranges of avian species in *Southern Ghats East*. Each vertical line indicates the elevational range of a species, during the boreal summer (red) or winter (blue). Points indicates seasonal mean elevations.

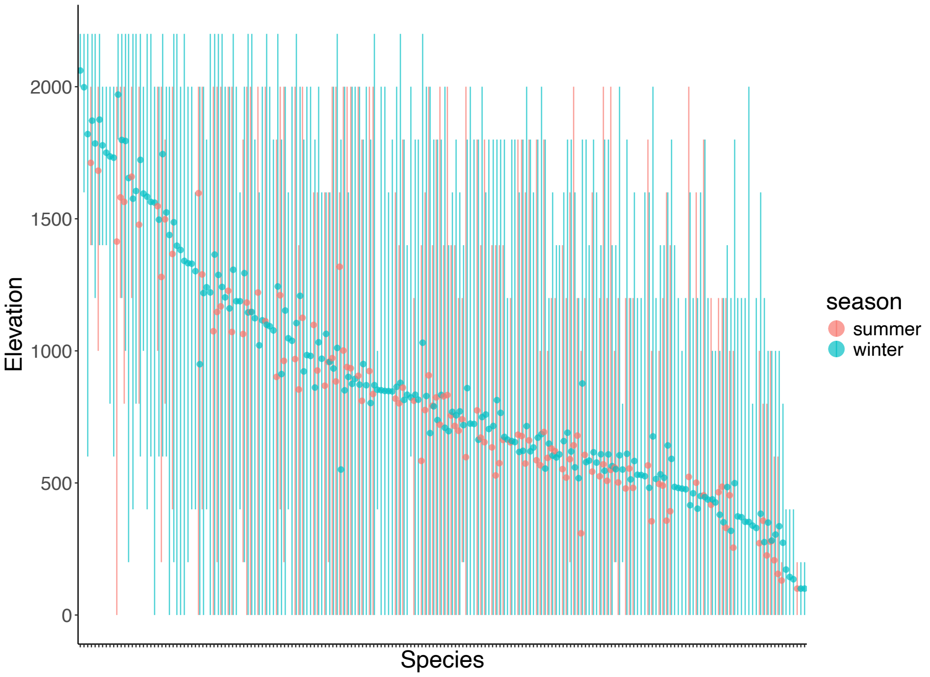

Fig. S64. Seasonal elevational ranges of avian species in *Southern Ghats West*. Each vertical line indicates the elevational range of a species, during the boreal summer (red) or winter (blue). Points indicates seasonal mean elevations.

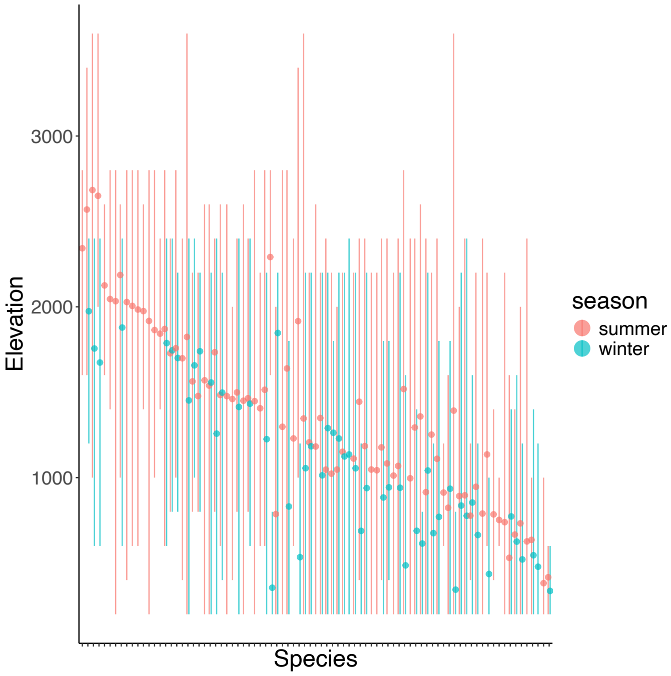

Fig. S65. Seasonal elevational ranges of avian species in *Swiss Alps*. Each vertical line indicates the elevational range of a species, during the boreal summer (red) or winter (blue). Points indicates seasonal mean elevations.

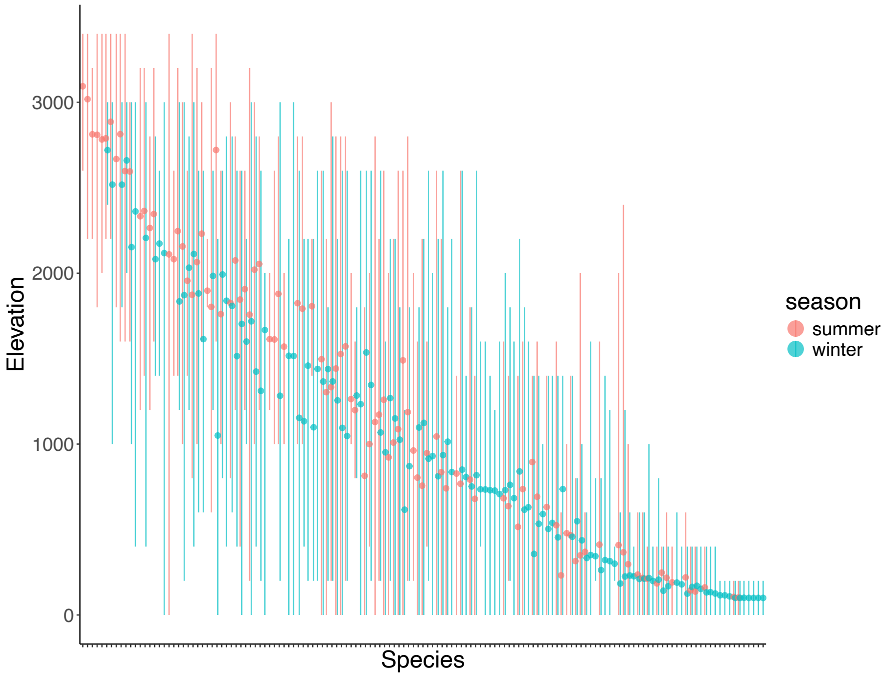

Fig. S66. Seasonal elevational ranges of avian species in *Taiwan East*. Each vertical line indicates the elevational range of a species, during the boreal summer (red) or winter (blue). Points indicates seasonal mean elevations.

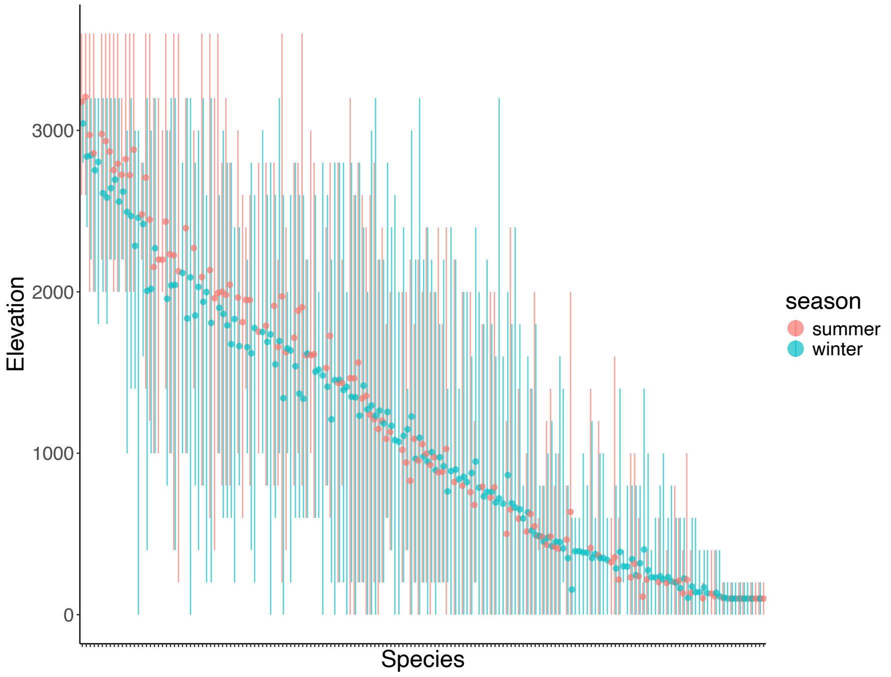

Figure S67: Seasonal elevational ranges of avian species in *Taiwan West*. Each vertical line indicates the elevational range of a species, during the boreal summer (red) or winter (blue). Points indicates seasonal mean elevations.

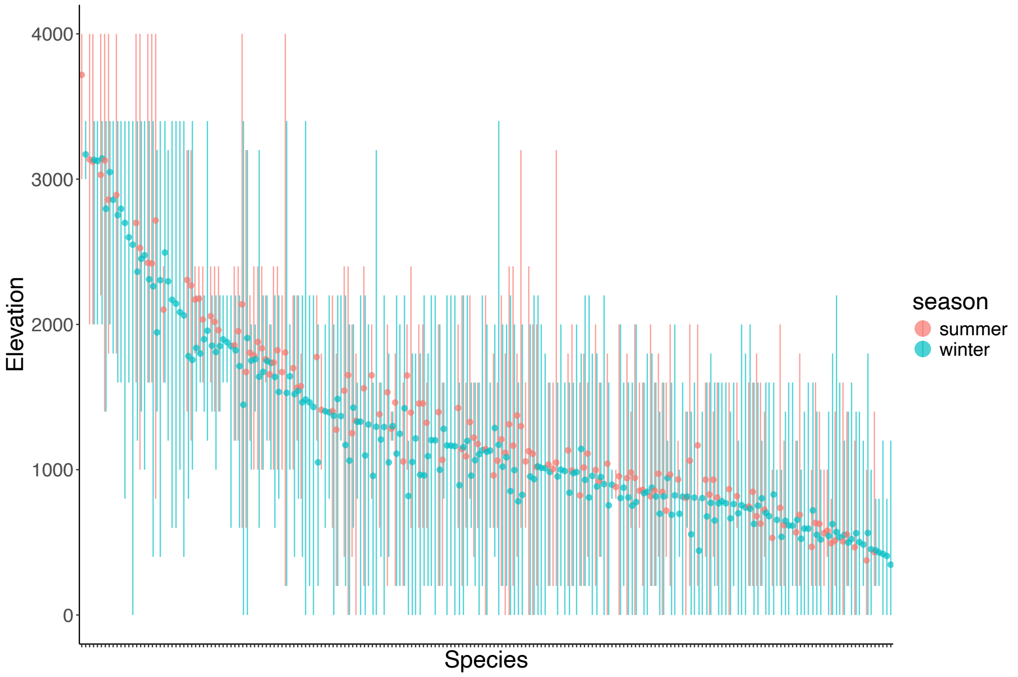

Fig. S68. Seasonal elevational ranges of avian species in *Veracruz*. Each vertical line indicates the elevational range of a species, during the boreal summer (red) or winter (blue). Points indicates seasonal mean elevations.

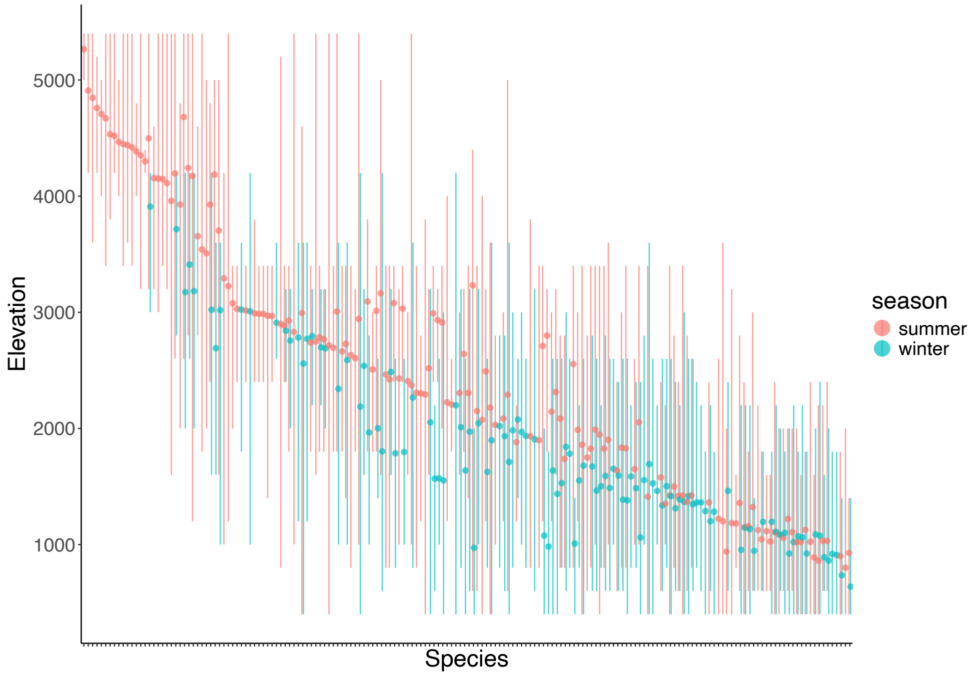

Fig. S69. Seasonal elevational ranges of avian species in *Western Himalayas*. Each vertical line indicates the elevational range of a species, during the boreal summer (red) or winter (blue). Points indicates seasonal mean elevations.

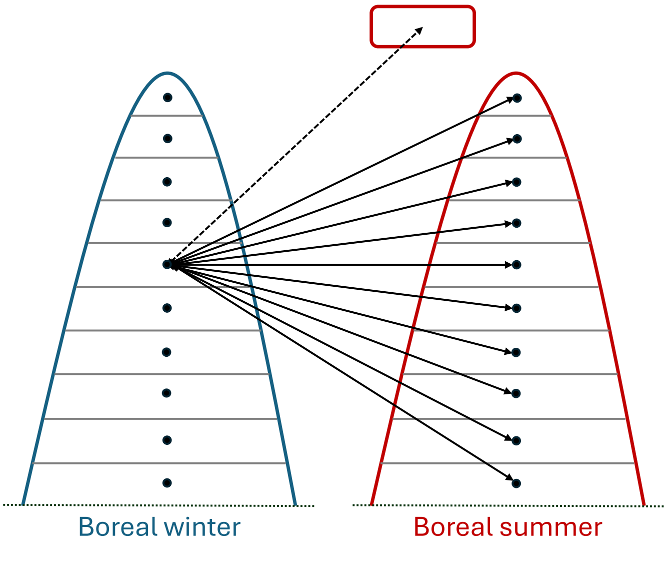

Fig. S70. Illustration of the bipartite network onto which the SEDS model was applied to simulate the seasonal distribution of avian populations along a mountain slope. The schematic shows the same mountain slope during boreal winter (in blue) and boreal summer (in red), with illustrative links between one node in boreal winter and all the possible nodes in the boreal summer. Nodes in the network represent 200m elevational bins at each season, which can be connected by bidirectional edges (links) that represent the movement of a population. A link between an elevational bin at a given season and the same elevational bin at the other season indicate a sedentary distribution for the population. There is also one additional node represented at the top of this schematic figure in red, which accommodates long-distance migrants going out of the mountain slope during the boreal summer.

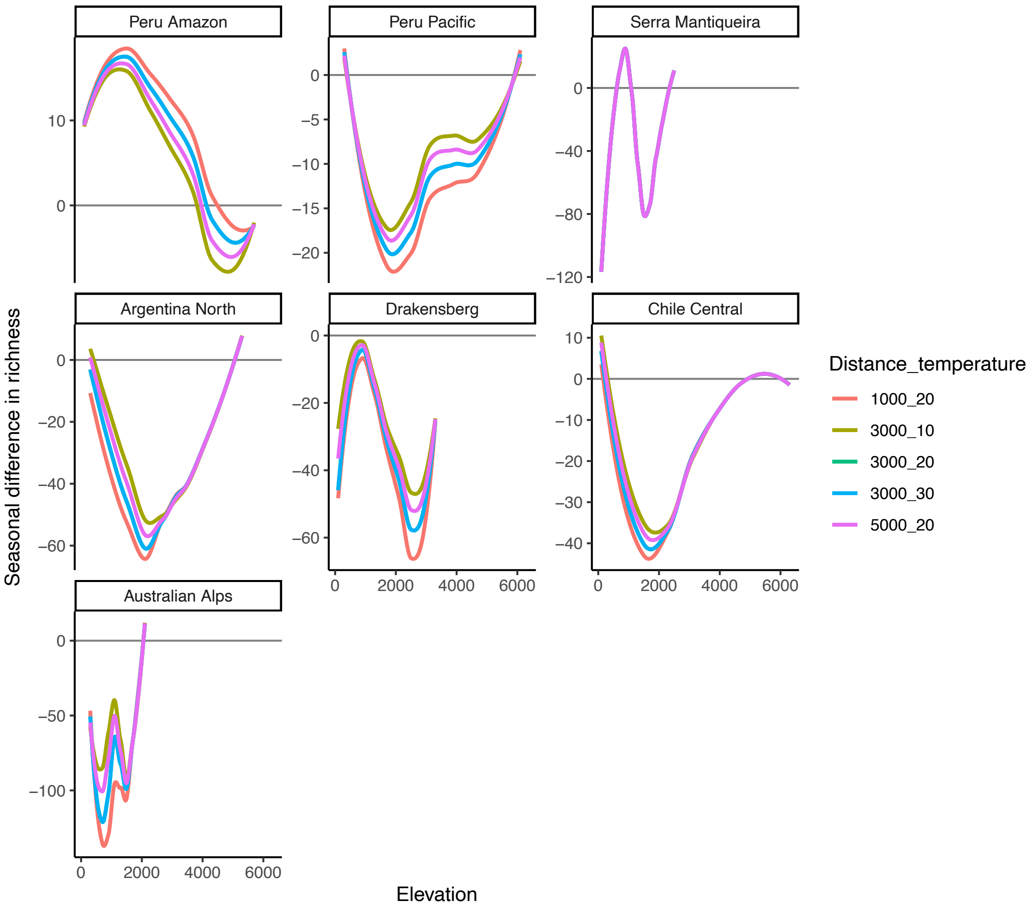

Fig. S72. Patterns of simulated seasonal difference in richness across mountain slopes in the southern hemisphere. Different values were chosen for the distance ($d = 1000$ and $d = 5000$) and temperature ($T_a = 10$ and $T_a = 30$) of the away node. The values used for the model presented in the main text are $d = 3000$ and $T_a = 20$.

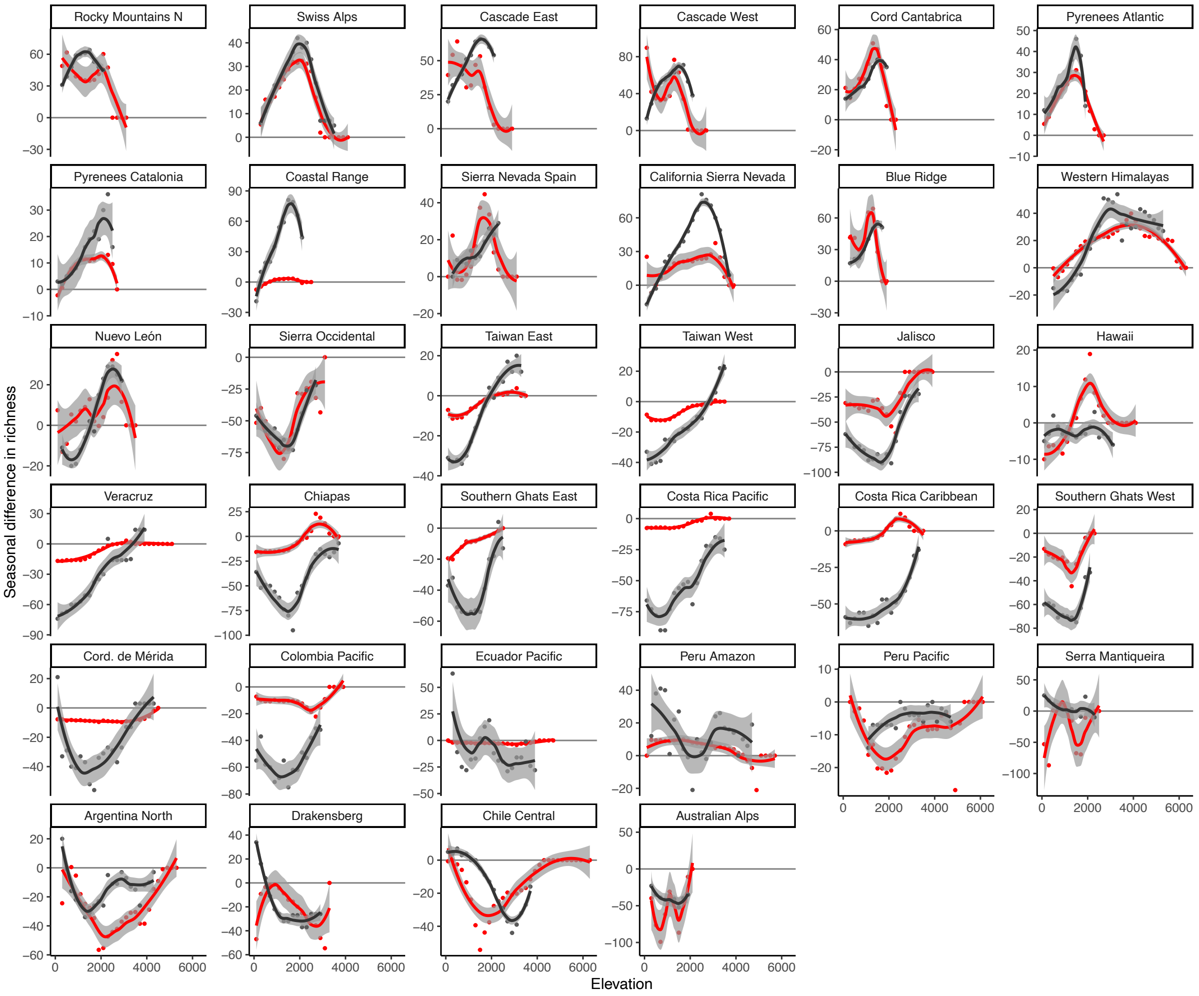

Fig. S72. Patterns of seasonal difference in richness across mountain slopes, with prediction from the SEDS model with $\theta = 50$. Each panel represent a mountain slope, ordered by decreasing latitude from top to bottom. The black lines indicate smooth curves fitted to the empirical estimates along the elevational gradients, and red lines indicate smooth curves fitted to the simulated estimates along the elevational gradients.

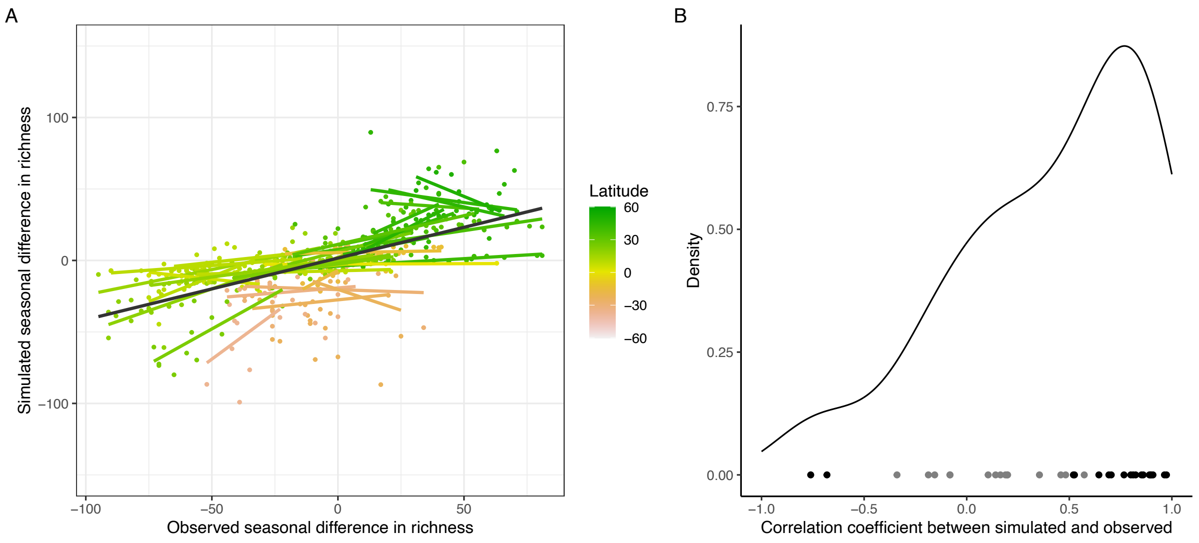

Fig. S73. Results for the SEDS model with $\theta = 50$. (A) Relationship between the seasonal difference in richness simulated by the SEDS model and estimated empirically. Each slope corresponds to a line of best fit for a mountain slope, and each point correspond to a 200m elevational bin, both colour-coded by latitude. The black line indicates the line of best fit for the whole dataset. (B) Density plot of the Pearson's correlation coefficients between model predictions and observation for the seasonal difference in richness, for all the mountain slopes in our dataset. Grey and black points indicate correlation coefficients that are non-significant and significant, respectively, based on correlation statistical tests.

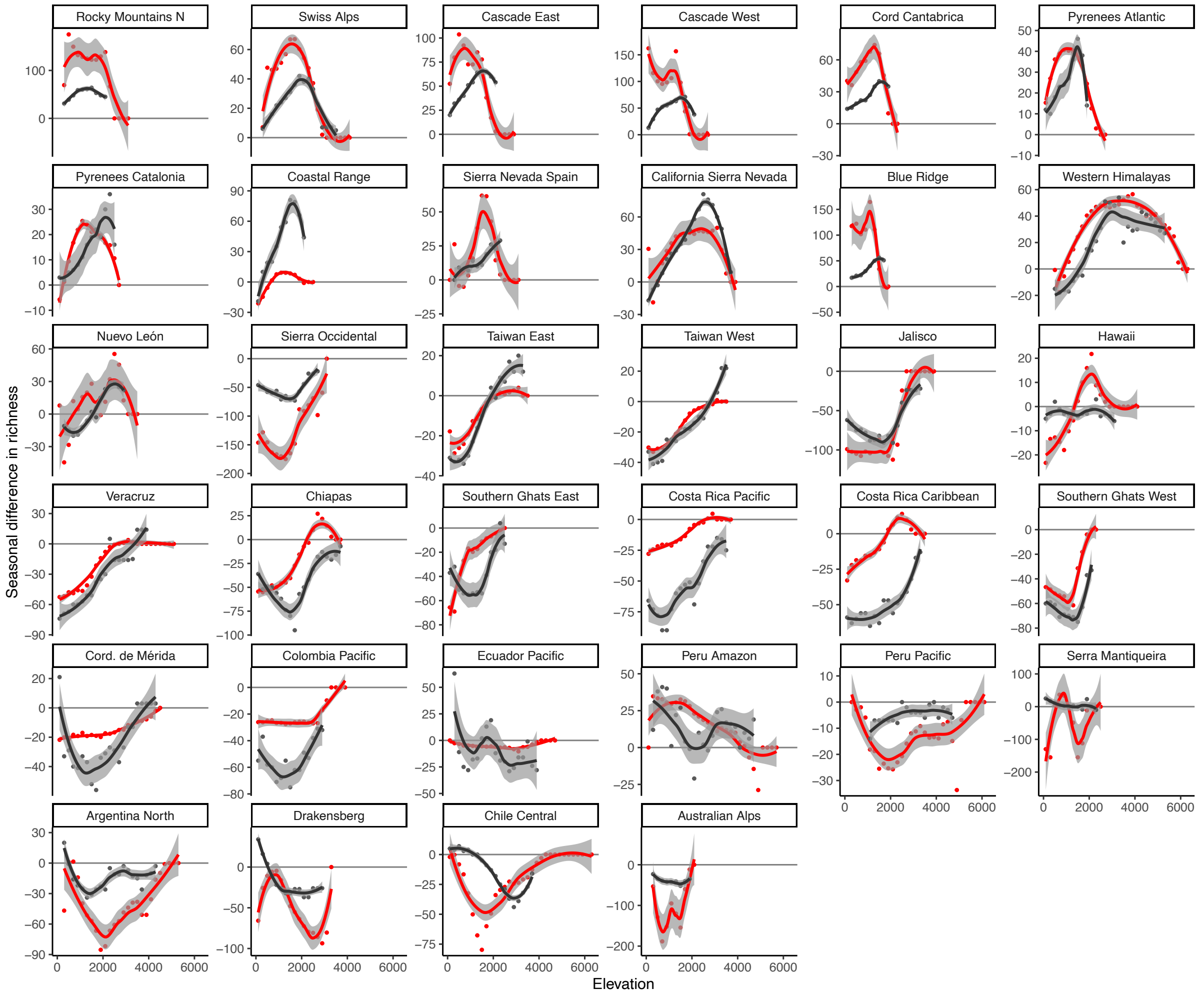

Fig. S74. Patterns of seasonal difference in richness across mountain slopes, with prediction from the SEDS model with $\theta = 200$. Each panel represent a mountain slope, ordered by decreasing latitude from top to bottom. The black lines indicate smooth curves fitted to the empirical estimates along the elevational gradients, and red lines indicate smooth curves fitted to the simulated estimates along the elevational gradients.

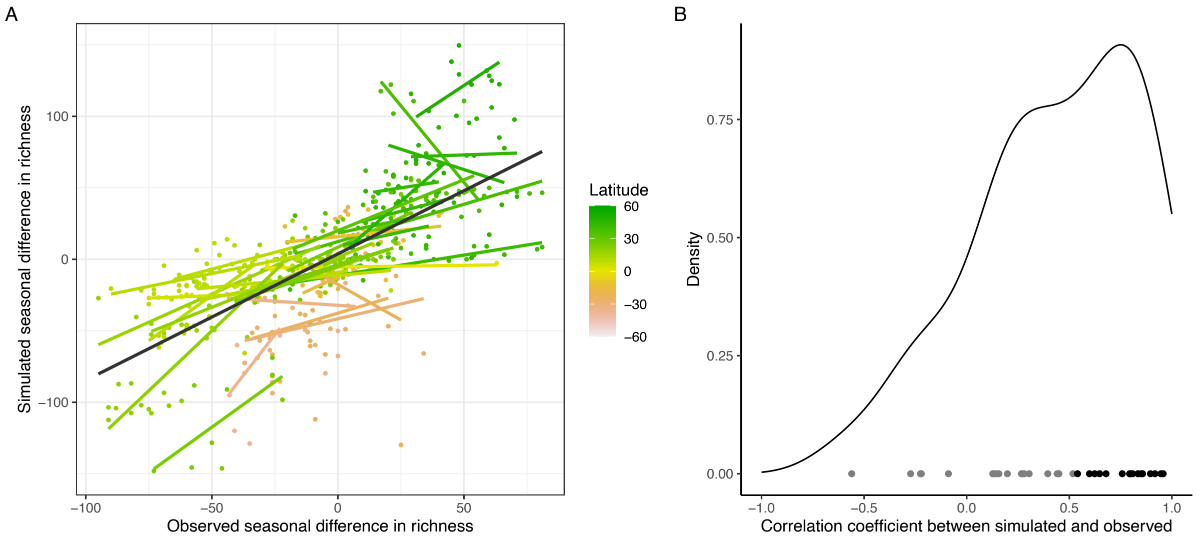

Fig. S75. Results for the SEDS model with $\theta = 200$. (A) Relationship between the seasonal difference in richness simulated by the SEDS model and estimated empirically. Each slope corresponds to a line of best fit for a mountain slope, and each point correspond to a 200m elevational bin, both colour-coded by latitude. The black line indicates the line of best fit for the whole dataset. (B) Density plot of the Pearson's correlation coefficients between model predictions and observation for the seasonal difference in richness, for all the mountain slopes in our dataset. Grey and black points indicate correlation coefficients that are non-significant and significant, respectively, based on correlation statistical tests.

Supplementary Table

Table S1. Results of a sensitivity analysis for the migration threshold. Values indicate the proportion of altitudinal migrant populations that are not tracking temperature seasonally as much as if they had simply stayed sedentary, for two thresholds determining migratory populations, 300m and 400m, which are more stringent that the one used in the main analysis (200m). Values are provided for the global dataset and for mid latitudes (defined as mountain slopes located between 15°S–35°S or 15°N–35°N).

**Migration threshold**	**Global dataset**	**Mid latitudes**
300m	33.0	22.1
400m	28.4	20.6
